# Improving Proactive Primary Health Care After Gestational Diabetes: Protocol for Implementation and Evaluation of a Quality Improvement Collaborative Program in General Practice (GooD4Mum) and Baseline Practice Characteristics

**DOI:** 10.2196/89207

**Published:** 2026-08-28

**Authors:** Rachel Canaway, Samantha Kozica-Olenski, Anusha Ramani-Chander, Wendy Shepherdley, Sharleen O’Reilly, Tesfaye R Feyissa, Vincent Versace, Siew S Lim, Emily Callander, Melinda Morrison, Mark Morgan, Jane Speight, Douglas I R Boyle, Helena Teede

**Affiliations:** 1 Department of General Practice and Primary Care, Faculty of Medicine, Dentistry and Health Sciences, The University of Melbourne, Parkville, Victoria, Australia; 2 Monash Centre for Health Research & Implementation (MCHRI), Monash University, Level 1, 43-51 Kanooka Grove, Clayton, Victoria, 3168, Australia, 61 3 8572 2644; 3 School of Agriculture and Food Science, University College Dublin, Dublin, Leinster, Ireland; 4 Deakin Rural Health, School of Medicine, Faculty of Health, Deakin University, Warrnambool, Victoria, Australia; 5 Department of Population Health, Dasman Diabetes Institute, Geohealth Lab, Kuwait, Kuwait; 6 Health Systems and Equity, Eastern Health Clinical School, Monash University, Melbourne, Victoria, Australia; 7 Diabetes Australia, Turner, Australian Capital Territory, Australia; 8 Faculty of Health Sciences & Medicine, Bond University, Gold Coast, Queensland, Australia; 9 Australian Foundation for Diabetes Research, Carlton, Victoria, Australia

**Keywords:** Australia, diabetes prevention, gestational diabetes, implementation evaluation, primary care, protocol, Quality Improvement Collaborative

## Abstract

**Background:**

Gestational diabetes mellitus (GDM) is increasingly common, with short- and long-term health risks. Building on the GooD4Mum pilot, which demonstrated quality improvements in general practice for care after GDM, this project implemented and evaluated a primary care Quality Improvement Collaborative (QIC) program to optimize identification, recall, screening, and referral of patients after GDM.

**Objective:**

This study aimed to assess the effectiveness of QIC activities relative to usual practice for improving general practice clinicians’ provision of follow-up and screening of patients with a history of GDM to ultimately support the onset of type 2 diabetes.

**Methods:**

A 21-month, prospective non–randomized controlled trial (at practice level) was conducted, matching intervention practices 1:1 with controls. The QIC intervention is compared with care-as-usual in general practice to review implementation, effectiveness, and economic outcomes. For 18 months, the intervention practices engaged with the GooD4Mum QIC program, including education, training, resources, and Plan-Do-Study-Act (PDSA) cycles to implement locally relevant improvement activities. A clinical decision support system aided in the identification, screening, and tracking of patients with a history of GDM. Control practices provided care as usual. Primary outcomes are practice-level proportions of women with recorded type 2 diabetes screening, modifiable cardiometabolic risk factors, and referral to a diabetes prevention program. Secondary outcomes examine changes in care processes, adherence to clinical standards, and fidelity of intervention delivery. Outcome analyses use clinical data derived from practices via automated extraction. Baseline comparisons use *t* tests or chi-square tests. Primary and secondary outcomes are analyzed by repeated-measures ANOVA and/or cluster-adjusted generalized estimating equations, accounting for practice-level clustering, and sensitivity analyses include a per-protocol approach. Implementation outcomes are assessed through longitudinal qualitative interviews with practice leads, support staff, and stakeholders, guided by the CFIR (Consolidated Framework for Implementation Research) and the RE-AIM (Reach, Effectiveness, Adoption, Implementation and Maintenance) framework. Cost consequence analysis uses clinical activity and practice-reported data.

**Results:**

The program ran in 9 general practices between April 2024 and September 2025, with data collection until December 2025. Practice characteristics and protocol deviation are summarized. Detailed outcome, cost consequence, and implementation process evaluations will be reported elsewhere.

**Conclusions:**

The protocol offers a novel quality improvement approach enhanced by clinical decision support and automated data extraction to optimize identification, screening, and provision of lifestyle advice toward reducing type 2 diabetes after GDM. The evaluation is designed to generate actionable insights and a scalable implementation toolkit to improve care after gestational diabetes.

## Introduction

### Background

Gestational diabetes mellitus (GDM) is a public health problem affecting 18% of pregnancies in Australia [1], between 12% and 30% in regions worldwide [2], and has both short-term pregnancy and birth complications and long-term implications for birth-parent and offspring [3]. GDM is also one of the strongest predictors of type 2 diabetes mellitus (T2DM), with risks exacerbated by higher BMI and excess gestational weight gain, which if retained postpartum further increases the maternal risk of obesity, T2DM, and heart disease [4,5]. These long-term risks also extend to offspring [3,6]. The health and socioeconomic burden of long-term population management of T2DM is high, with a 2021 global prevalence of 6.1%, expected to rise to above 10% in 89 countries by 2050 [7].

Targeting high-risk groups to slow or prevent progression to T2DM is vital to improve population health and lower health care costs [5]. People with a history of GDM are a key target group for prevention. Lifestyle interventions that include healthy eating and physical activity are known to prevent T2DM [8,9]. Diabetes prevention programs have demonstrated efficacy in the general population with significant reductions in T2DM, including in those with a GDM history [10,11]. After GDM, clinical guidelines advise blood glucose testing at 6‐12 weeks postpartum and every 1‐3 years as clinically indicated [12]. There are, however, implementation challenges related to identification of those with prior GDM, low-risk perception, and poor adherence to annual follow-up to screening [13], and significant life-stage specific barriers for women that impact postpartum engagement with lifestyle prevention activities [6,14,15].

Quality Improvement Collaborative (QIC) methods informed by the Boston Institute for Healthcare Improvement [16-18] have been used to activate behavioral changes at individual, team, or system levels [19]. The QIC methodology, often used in general practice (primary care), involves providing audit and feedback to practices together with best-practice guidance and team capacity-building advice. It is designed to encourage clinicians and support staff to change practice and deliver improvement in short time frames through implementing change in small, manageable cycles, and identifying where a change leads to an improvement [16]. QIC methods incorporate the Model for Improvement and Plan-Do-Study-Act (PDSA) cycles to encourage development of best practices around care, which has the potential to improve outcomes and reduce costs at the population level [20]. QICs have been found effective in primary and secondary care and have been able to significantly improve clinical processes and outcomes [21], including the quality of T2DM care in Australian general practice [22]. However, lack of robust application and fidelity in implementing QIC and PDSA has also limited efficacy, and capturing implementation learnings remains important [23,24]. For example, a review of 120 PDSA-based quality improvement projects noted that 98% (n=118) reported improvements, but 40% (n=48) did not sufficiently document PDSA cycles for full analysis of key features: of those who did, just 3 adhered to all 4 key PDSA features, and methodological limitations made it “difficult to draw firm conclusions about the causality of the reported improvements in quality of care.” The authors concluded need to continuously improve such quality improvement methods [24].

### Rational and Context

The GooD4Mum initiative builds on past programs and research and recognizes the need to adapt quality improvement methods in primary care to make implementation and adherence easier to maximize success. The GOAL (Good Ageing in Lahti Region) project in Finland demonstrated feasibility for T2DM prevention using a QIC-informed lifestyle intervention in primary care, showing positive outcomes sustained for up to 3 years [25,26]. This was built upon in the *Greater Green Triangle Diabetes Prevention Study* in which GooD4Mum co-designer Professor James Dunbar and colleagues demonstrated feasibility of the QIC-informed intervention in Australian primary care [27] and was subsequently scaled up across Victoria (Australian state) as the Diabetes Victoria *Life!* program, which incorporates peer support, education, and coaching [28,29]. Evaluation of *Life!* by Dunbar and other GooD4Mum investigators (Versace and O’Reilly) showed sustained T2DM and cardiovascular risk reduction at 12 months, but lack of uptake by postpartum mothers [30]. *Life!* has been adapted to support prevention after GDM in the online *Life! After Gestational Diabetes* program, with current GooD4Mum investigator Dr Siew Lim being instrumental in this new program [31-34]. *Life! After GDM* was introduced in 2023 to meet the needs of postpartum mothers after GDM; it has state-based funding, participation is free, and includes initial health assessment, individual goal setting, online group sessions, nutrition and exercise resources, support with sleep and stress management, and opportunity to connect with other mothers with similar experiences [31].

Missing from these earlier initiatives was focus on diabetes prevention after GDM in general practice. In 2014‐2015, a 12-month pilot of the *GooD4Mum* program introduced QIC methods to 15 general practices in Australia to enhance identification, screening, and prevention of those with previous GDM. The goal was to capture all patients with a diagnosis of GDM (not type 1 or type 2 diabetes), who met the Royal Australian College of General Practitioners (RACGP) criteria of an active patient (at least 3 visits in the past 2 years) [35], and ensure blood glucose screening within the 12-month study, which, in some cases, was more frequent than recommended in clinical guidelines (every 1‐3 years depending on risk factors [12]). The GooD4Mum pilot increased postpartum blood glucose screening from 43% to 60%, but barriers remained relating to implementing practice change and increasing uptake of healthy lifestyle prevention programs in this target group [14,19,36]—a control group was also recommended.

In parallel, studies related to the *Mothers After Gestational Diabetes in Australia* diabetes prevention program [32-34], which also built upon content from the *Greater Green Triangle Diabetes Prevention Study* [37,38]. Study colead Professor Boyle codeveloped The University of Melbourne’s *Data for Decisions* [39], GRHANITE clinical data extraction software [40], and clinical decision support tool *Future Health Today* (FHT) [41-43] (Textbox 1).

Textbox 1. **Data collection capabilities.**

- Data for Decisions is an ethics and legal framework for capture, curation, and use of deidentified electronic medical record (EMR) data to be made available for approved research, teaching, audit, and surveillance purposes. All researchers seeking access to this EMR data must obtain the necessary ethics and independent Patron Data Governance Committee approvals and commit to annual project reporting [39,44].
- GRHANITE is a privacy-preserving data extraction software tool that, when installed on general practice computers, deidentifies data from the EMR, generates unique patient identifiers, and securely sends the data to the secure Patron repository. GRHANITE includes consent management and person-identifying data are not extracted, ensuring robust data analysis while maintaining patient confidentiality [40,44,45].
- *Future Health Today* is a quality improvement software platform developed for general practice settings. It features an interactive dashboard to improve identification, recall, and management of patients, and integrates with existing clinical record systems to provide point-of-care decision support (pop-ups) to streamline patient management processes [43].

The current GooD4Mum program uses the QIC methodology of the GooD4Mum pilot [19,36] and seeks to address the barriers to implementing practice change and ensuring healthy lifestyle promotion for the target population. The updated GooD4Mum program is longer and therefore allows more time for supported practice change and education. It introduces the quality improvement and decision support software FHT as a tool to prioritize and manage recall of the GDM cohort. The pilot study GDM patient cohorts included only mothers who met the RACGP’s definition of an “active” patient, that is, having attended the practice or service 3 or more times in the past 2 years [35]. This study does not use this designation in acknowledgment that all women with a history of GDM are at risk of T2DM and many may not visit their general practitioner (GP) with such frequency. Another difference is that rather than practices needing to provide in-house diabetes prevention and health promotion activities, referral to Diabetes Victoria’s free *Life!* [29] or *Life! After GDM* [31] program is instead encouraged. *Life! After GDM* is an online program designed to overcome the life stage–specific barriers that mothers reported prevented attendance to the regular *Life!* program, which includes face-to-face meetings and tends to attract older people.

The Methods section describes the protocol that was implemented, and the planned statistical analysis, implementation, and cost consequence evaluation. The Results section summarizes the intervention and control practice characteristics and where there was deviation of protocol. Activities commenced in general practices in April 2024 and ceased on September 30, 2025, with collection of electronic medical record (EMR) data for outcome analysis until December 31, 2025.

### Hypotheses

Compared with care as usual practices, general practices participating in the GooD4Mum program will show a significantly greater change in the rate of glycemic screening aligned to clinical guidelines among the target population compared with baseline, increased referral to T2DM prevention programs (eg, the *Life!* program), and significantly increased capture of modifiable cardiometabolic risk factors and clinical measurements among the target population.

### Aim and Objectives

#### Aims

The study aims to assess the effectiveness of audit and QIC activities (the implementation strategy), relative to usual practice (implementation comparison) for improving general practice clinicians’ (implementation population) provision (implementation outcome and target of the implementation strategy) of follow-up, screening, and risk reduction (clinical care) of patients with a history of gestational diabetes to ultimately support prevention of onset of type 2 diabetes.

#### Objectives

The primary and secondary objectives are shown in Textbox 2.

Textbox 2. **Primary and secondary objectives.**

Primary objectives:

- Primary objective 1: Determining whether access to a practice-based gestational diabetes register facilitates patient identification and improved recall rates of women with prior gestational diabetes mellitus (GDM).
- Primary objective 2: Determining whether engagement with Quality Improvement Collaborative (QIC) activities can improve rates of blood glucose screening, recording of metabolic risk factors and clinical measures, and referral to a diabetes prevention program, and so promote chronic illness risk reduction.

Secondary objectives:

- Secondary objective 1: Building capacity for QIC methods in general practice.
- Secondary objective 2: Assessing program reach, effectiveness, adoption, implementation, and maintenance using the Reach, Effectiveness, Adoption, Implementation and Maintenance (RE-AIM) evaluation framework [46].
- Secondary objective 3: Identifying program implementation enablers and barriers (for patients, general practitioners, practice nurses, other clinic staff, and supporting organizations) and successful components of care delivery to inform development of a toolkit for broader implementation.
- Secondary objective 4: Establishing the costs of implementing the program and the most efficient way to provide it across other sites.
- Secondary objective 5: Determining guideline-practice gaps for early detection and prevention of T2DM among people with prior GDM.

By the end of the project, the research team seeks to:

- generate real-world implementation knowledge to inform scale-up of the GooD4Mum quality improvement approach for diabetes prevention focused on practice level,
- develop a co-designed GooD4Mum implementation toolkit to support program scale-up, and
- determine guideline-practice gaps for early detection and prevention of T2DM among women with previous gestational diabetes and develop resources to address these gaps (eg, revised Royal Australian College of General Practitioners and Diabetes Australia clinical guidelines).

This protocol is reported alongside the SPIRIT (Standard Protocol Items: Recommendations for Interventional Trials—Checklist 1) and the SQUIRE 2.0 (Standards for Quality Improvement Reporting Excellence) guideline (Checklist 2).

## Methods

### Study Design

A 21-month, prospective non–randomized controlled trial in primary care was conducted, comparing a QIC intervention with care-as-usual passive controls at the practice level studying implementation, effectiveness, and economic outcomes. Ten to twelve intervention sites were planned, matched 1:1 with *Data for Decisions* participating control practices (using data fields shown in Multimedia Appendix 1). Using data-only controls reduces recruitment time and allows for efficient data collection. By matching control practices primarily on location [47,48] and Socio-Economic Index for Areas [49], impacts of any local health improving programs potentially including GDM populations will be similar between intervention and control practices. Changes in practice behaviors and effectiveness of the intervention at meeting the study aims and objectives will be determined by process and implementation evaluation framed by the CFIR (Consolidated Framework for Implementation Research) [50-52] and the RE-AIM (Reach, Effectiveness, Adoption, Implementation and Maintenance) framework [46,53-55], cost consequence economic analysis [56], and statistical analysis of outcome measures. Stakeholder interviews (Multimedia Appendix 2) occur throughout the trial as part of the implementation, process, and economic evaluation, including with representatives from 5 to 10 control practices invited 12 months after commencement, and with recalled patients from intervention practices who consent after responding to an optional survey (Multimedia Appendix 3). The breadth of evaluation acknowledges that implementation processes are complex and dynamic, reflecting the translation of strategic intentions into everyday practices. The evaluation plans are further described in this manuscript.

### Study Setting

General practices in the Australian state of Victoria and towns bordering Victoria (see Inclusion and Exclusion criteria), over an 18-month period, divided into 6 3-month-long activity periods.

### Description of the Intervention

**Figure 1.**
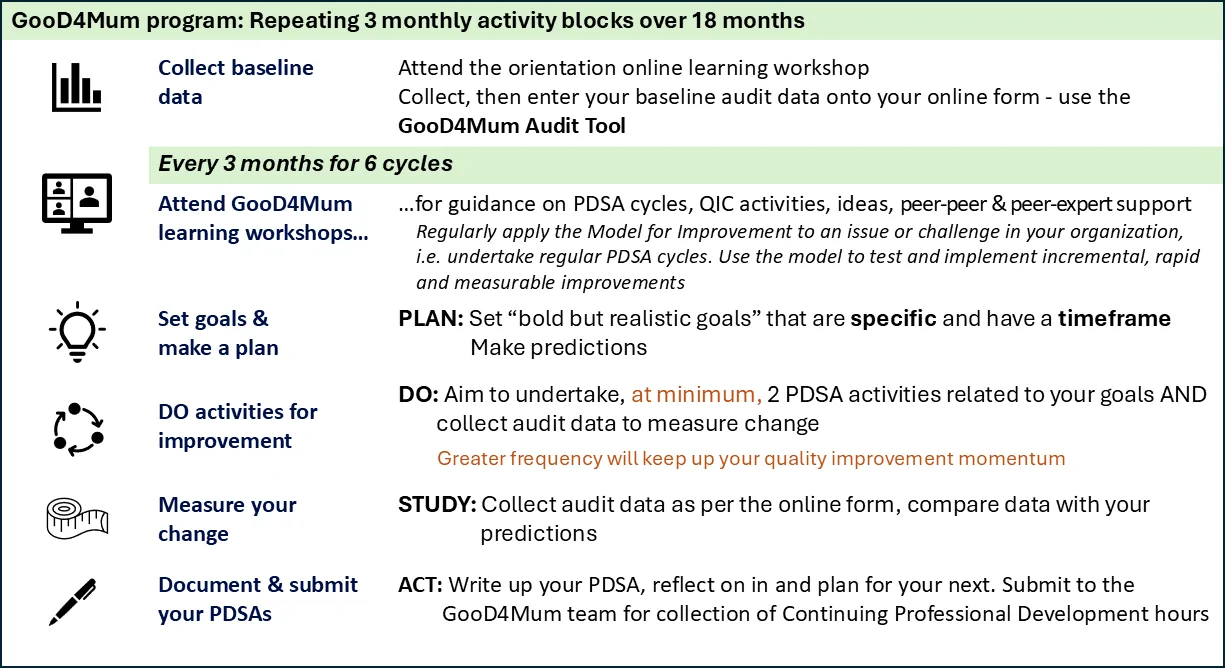
Summary of GooD4Mum activities for intervention practices. PDSA: Plan-Do-Study-Act; QIC: Quality Improvement Collaborative.

Each intervention practice designated a practice champion [57]—a GP, practice nurse (PN), or diabetes educator. Activities for intervention practices are summarized in Figure 1, characterized by 7 *interactive online learning workshops* (orientation plus 1 for each 3-month activity period) and six 3-monthly PDSA cycles. The learning workshops were led by experts, providing evidence-based information on management of patients after GDM, resources for optimal care concordant with clinical guidelines, and instruction on undertaking PDSA cycles for quality improvement. The workshops facilitated discussions to foster a collaborative community of practice where attendees shared experiences about success and challenges related to practice improvements.

Practice Champions or their delegates lead *PDSA cycles*, central to the QIC methodology, tailoring activities to align with their specific practice goals and workflow. The Model for Improvement, incorporating GooD4Mum Change Principles and Ideas (Multimedia Appendix 4), provided a framework for structuring activity cycles. During each cycle, practices should complete a minimum of 2 PDSA activities, including the collection of prescribed audit data (Table 1) submitted via an online form hosted on REDCap [58,59].

**Table 1.** GooD4Mum Audit Measures for submission by intervention practice at baseline and the end of each 3-monthly activity period^a^.

Measure	Description: counts of women on your primary GDM^b^ Register
Date	Date of data collection
Primary GDM Register	The number of women on the primary GDM register in FHT^c^ (ie, with a recorded diagnosis of GDM)
Secondary GDM Register	The number of women on the secondary GDM register in FHT (with possible GDM)
The number of women on the primary GDM register who...	
Recalled	...are awaiting consultation (appointment booked)
T2DM^d^ conversion	...now have a new diagnosis of type 2 diabetes
T2DM screening	...have had an OGTT^e^, FBG^f^, or HbA_1c_^g^ measurement recorded in the last 12 months
T2DM prevention care	...received a referral (eg, to Life! prevention program)
Postnatal follow-up of gestational diabetes and new pregnancy	...gave birth in the last 12 months ...gave birth in the last 12 months *and *have an OGTT or HbA_1c_ measurement recorded within 3 months of delivery
Capture of BMI	...have a recorded BMI ...have a BMI less than 25
Capture of risk factors	...have recorded smoker/ex-smoker/not recorded ...have hypertension ...have recorded cholesterol ...have recorded waist circumference ...have recorded physical activity

a Staff efforts include the number of people involved in gathering measures for this audit, and the approximate time in minutes spent gathering and recording data for this audit.

b GDM: gestational diabetes mellitus.

c FHT: *Future Health Today.*

d T2DM: type 2 diabetes mellitus.

e OGTT: oral glucose tolerance test.

f FBG: fasting blood glucose.

g HbA_1c_: hemoglobin A_1c_ (glycated hemoglobin).

Each practice was provided with a unique REDCap portal for submission of audit data and access to a summary of previously submitted audit data to track their progress. On commencement, each intervention practice used their REDCap portal to submit self-reported clinic information including number and full-time-equivalent work fractions of GPs, nurses, allied health, administrative, and other personnel at the practice, and number of active patients. Automated reminders prompted for baseline and 3-monthly submissions of audit data.

Intervention practices received support from the research team through in-person site visits, emails, phone calls, the online learning workshops, the GooD4Mum Handbook, newsletters, and other resources on a dedicated website for implementation practices. The GooD4Mum Handbook, an updated and enhanced version of the GooD4Mum pilot resource, provided a comprehensive guide featuring a participation checklist, activity summary (Figure 1), GDM information, an overview of the Model for Improvement (including PDSA cycles), clinical and quality improvement resources, suggestions for PDSA activities, and explanation of the quality improvement Change Principles that were collaboratively workshopped and tested (Multimedia Appendix 4). If not already installed, intervention practices enabled installation of FHT [43] and GRHANITE [45] and were provided with access to monthly online FHT support sessions.

### Key Technologies

With permission from the intervention and control practices, GRHANITE software deidentified clinical EMR data, which were then accessed by approved members of the research team to analyze study outcomes and compare with audit data submitted by participating intervention practices. FHT provided a technological solution for achieving secondary objective 1 (building capacity for QIC methods in general practice) and primary objective 1 (providing intervention practices with access to patient cohorts or GDM patient “registers”). The first register included patients with confirmed GDM diagnoses; the second listed patients with suspected GDM (no diagnosis of GDM but with oral glucose tolerance test [OGTT] results during pregnancy indicative of GDM). The bespoke FHT GDM module was developed during program development (Table 2). In addition to the registers, FHT provided 3 point-of-care pop-up alerts, each offering concise recommendations for the target population of patients with a history of GDM who have not been diagnosed with type 1 or type 2 diabetes (Table 2). All alerts linked to current evidence-based clinical guidelines, enabling point-of-care access to up-to-date information [41]. The FHT “dashboard” enabled practices to (1) review patients’ clinical measures and risk factors, (2) prioritize patient recall, and (3) manage PDSA quality improvement activities. Aggregated summaries of cohort counts were provided by the FHT team to the researchers for analysis and triangulation with Patron data. Individual patient information was never accessible to researchers from FHT reports.

**Table 2.** GooD4Mum: point-of-care alerts generated by Future Health Today.

Clinical indicator or FHT^a^ algorithm (patient cohorts in FHT)	Point-of-care pop-up (alert) recommendation	GooD4Mum primary outcome measure
FHT cohort 1: diagnosed GDM^b^ (*has a coded, current or previous diagnosis of gestational diabetes mellitus*).	*High risk of type 2 diabetes. Consider referral to Life! Program*^c^.	Proportion of patients with a history of GDM referred to the *Life!* diabetes prevention program.
FHT cohort 2: possible GDM (*has OGTT*^d^ *since 2015*^e^ *consistent with GDM but no recorded diagnosis*).	*Possible historical GDM diagnosis. Review historical OGTT results and ask patient about GDM history*.	Aligned with primary objective 1, facilitating identification of patients who may have GDM adding to GDM register.
FHT cohort 3: glucose screening (*history of GDM and does not have blood glucose screening results recorded in the last 12 months*).	*High risk of type 2 diabetes. Recommend fasting blood glucose level or HbA_1c_ testing*.	Proportion of patients with a history of GDM who have completed blood glucose screening as per clinical guidelines.

a FHT: *Future Health Today.*

b GDM: gestational diabetes mellitus.

c Participants were educated about the availability of the newer *Life! After GDM* [31] and the longer established *Life!* [29] programs, both run by Diabetes Victoria, and encouraged to use clinical judgment on which to refer.

d OGTT: oral glucose tolerance test.

e In 2015, new diagnostic criteria were introduced that reduce the thresholds at which GDM is diagnosed. Recommendations incorporated into FHT must align with current therapeutic guidelines.

### Sample Size

The study was powered to detect a minimum difference of 10% in screening rates between the control and intervention practices achieved by including as few as 16‐18 general practices across intervention and control arms, with an average cluster size of 15 women per practice per annum (total of 270 eligible women). By recruiting 10‐12 general practices, we allowed for the potential loss of 2‐3 practices by 18 months (eg, practice closure and practice withdrawals).

The sample size was based upon an SD of 25% of the number of eligible women who were in each of the GooD4Mum pilot practices [19], 5% significance level, and 90% power, and accounted for the general practice clustering effect (intraclass correlation coefficient set at 0.001). This number of practices was greater than required to meet the minimum requirement for reliable analysis of a cluster design. Calculations were made using Stata menu-driven command “clustersampsi” [60,61]. This sample size with a 10% difference was selected to (1) enable the detection of a difference between intervention practices and control practices, (2) meet the optimal rules for cluster size and analysis, (3) detect anything over a doubling of the 5% who currently participate in a diabetes prevention program in this population [62], and (4) align with the percentage involved in prevention assessment in the GooD4Mum pilot.

(1) enable detection of a difference between intervention practices and control practices, (2) meet the optimal rules for cluster size and analysis, (3) detect anything over a doubling of the 5% who currently participate in a diabetes prevention program in this population [62], and (4) align with the percentage involved in prevention assessment in the GooD4Mum pilot.

(1) enable detection of a difference between intervention practices and control practices;, b) (2) meet the optimal rules for cluster size and analysis;, c) (3) detect anything over a doubling of the 5% who currently participate in a diabetes prevention program in this population [62];, and (4) align with the percentage involved in prevention assessment in the GooD4Mum pilot.

(1) enable detection of a difference between intervention practices and control practices, (2) meet the optimal rules for cluster size and analysis, (3) detect anything over a doubling of the 5% who currently participate in a diabetes prevention program in this population [62], and (4) align with the percentage involved in prevention assessment in the GooD4Mum pilot.

### Inclusion and Exclusion Criteria

#### Intervention General Practice Eligibility

The criteria are listed as follows:

- Location: Victoria, Australia, or towns bordering the state of Victoria that can access Victorian health services including Diabetes Victoria’s *Life!* programs (which at the time of implementation accepted participants from 49 non-Victorian, border-town postcodes).
- Clinical software: Best Practice or Medical Director (compatible with FHT and GRHANITE).
- Participation: At least 1 GP actively involved in GooD4Mum. One team member willing to be the “GooD4Mum Practice Champion.”
- Technical requirements: Installation of GRHANITE and FHT on Windows 10 (minimum) computers and allow access to deidentified EMR and FHT usage data. If applicable, all practices sharing a server must participate (because of the complexity of isolating data from a single practice when a server is shared across multiple practices).
- Quality improvement: Staff willingness to participate in program-related activities.
- Exclusion criteria: Concurrent involvement in similar GDM care improvement programs. Exclusive use of Apple Mac workstations.
- Additional requirement: Legal agreement for participation and acceptance of software end user license agreements. Agreement includes consent to allow administrators of the *Life!* program to share aggregated information about referrals received from the practice over the course of the trial.

#### “Data Control” General Practice Eligibility

The eligibility criteria are listed as follows:

- Characteristics: General practices that contribute deidentified EMR data to the Patron primary care data repository [45] as part of the *Data for Decisions* program [39] via GRHANITE software.
- Selection process: Control practices matched 1:1 with intervention practices from the pool of Victorian (and border towns) practices contributing to the Patron primary care data repository [45]. A blinded statistician makes the final selection based on location, practice size, and demographic characteristics outlined in Multimedia Appendix 1 (not randomized).
- Participation: The *Patron Data Governance Committee* decides on behalf of the practices it represents whether to allow use of deidentified EMR data for comparative “control”; approval is given with adherence to the *Patron Data Governance Framework* [63]. Control practices provide “care-as-usual” and are not initially notified of their selection.
- Data provision: Deidentified EMR data are curated and blinded by the data manager and made available to ethics and data governance committee–approved researchers within a Secure Research Environment (SRE) established and managed by The University of Melbourne. Although deidentified, patient data are considered sensitive. Before gaining access to the SRE, researchers complete mandatory training to demonstrate knowledge of compliance around management and use of the data.

#### Control Practice Representative Eligibility (Evaluative Interviews)

The eligibility details are listed as follows:

- Characteristics: Staff at allocated control general practices.
- Selection process: Staff self-nominate by responding to an Expression of Interest.
- Participation: A GP, PN, or diabetes educator from control practices to provide perspectives on “care-as-usual” management of patients with GDM.
- Exclusion criteria: Younger than 18 years.

#### Patients

The criteria are listed as follows:

- Characteristics: Patients with a history of GDM at intervention general practices who do not have type 1 or type 2 diabetes.
- Selection process: Staff or clinicians at intervention practices can invite target population patients to participate in an anonymous survey after they have been recalled for diabetes prevention, including blood glucose screening.
- Participation: Completion of the brief practice unique online survey. Within the survey was provision to provide contact details if the participant wished to provide additional information about their recall and consultation via a confidential interview with the researchers.
- Exclusion criteria: Younger than 18 years. No history of GDM. Has type 1 or type 2 diabetes. Has not been recalled by the practice related to GDM management.

### Recruitment

Recruitment of intervention practices was via Expression of Interest managed by The University of Melbourne–based researchers, with access to the Victorian primary care practice–based Research and Education Network [64] client management database of general practices. Participating intervention practices receive Aus $500 upon completion of onboarding (return of consent forms, legal agreement, software end user license agreements, and installation of GRHANITE and FHT), and $500 at completion of the study if all deliverables and other requirements are met to the researchers’ reasonable satisfaction (Aus $1=US $0.65 as of April 1, 2024, and US $0.66 as of September 31, 2025). Practice Champions received an Aus $200 gift card as a thank you for driving implementation at the practice–paid upon completion. Practice staff who complete interviews at intervention or care-as-usual control practices receive gift cards for each interview. Participating clinicians could claim continuing professional development certificates including accredited RACGP or Australian College of Rural and Remote Medicine continuing professional development hours for GP members.

### Outcome Measures

#### Primary Outcomes

The primary outcome measures aligned with primary objective 2: proportions of the target patient population who have completed blood glucose screening (OGTT and/or fasting blood glucose and/or hemoglobin A_1c_ [HbA_1c_]) as per clinical guidelines, have BMI captured in the EMR, and have been referred to Diabetes Victoria’s *Life!* or *Life! After GDM* healthy lifestyle programs [29,31]. Noting that assessment of glycemic status and BMI have been identified as key variables in the post-GDM Core Outcome Set [65]. Referral to *Life!* or *Life! After GDM* is a quantifiable proxy for the provision of health-promoting information to patients after GDM.

#### Key Secondary Outcomes

Secondary outcome measures are changes among the target population in the following: (1) Requests for blood glucose screening (OGTT and/or fasting blood glucose and/or HbA_1c_) to determine the ratio of requests issued versus completed screens. (2) The number of patients newly diagnosed with prediabetes and the number of patients newly diagnosed with T2DM. (Earlier detection due to increased rates of screening can lead to better outcomes.) (3) Rate of recording the following risk factors in the clinical record: blood pressure, cholesterol, BMI, waist circumference, smoking, and physical activity. Routine recording of risk factors during general practice consultations instills in patients a sense of their importance and can be used as a conversation starter in health coaching or diabetes prevention. (4) The number of patients who gave birth in the previous year *and* had appropriate glucose testing recorded within 3 months of delivery.

All measures in Table 1 will be reported as outcome measures with triangulation between self-collected audit measures and the same measures extracted by the researchers from the deidentified EMR (Patron) data. Other measures are described in the SPIRIT checklist (Checklist 1) and include reviewing outcome measures using the cohort of patients with GDM who meet the RACGP’s definition of an active patient (ie, attended the practice 3 or more times in the past 2 years [35]).

### Data Sources

Data sources to measure trial outcomes, evaluate the trial (based on the CFIR and RE-AIM frameworks), and perform the economic evaluation are provided in Table 3. The EMR data fields accessed via the Patron primary care data repository are summarized in Multimedia Appendix 1. An example online learning workshop evaluation survey is shown in Multimedia Appendix 5. Evaluative data collection extended for 3 months after cessation of the 18-month trial and allowed for appointment completion among patients recalled within the program period.

**Table 3.** GooD4Mum data types, source, and purpose.

Data type	Source	Primary data custodian	Purpose
Electronic medical record clinical and administrative data (nonperson identifying)	Intervention and control practices	The University of Melbourne Patron primary care data custodian [39,45]	Primary and secondary outcome measures, and contributing to economic evaluation
Electronic medical record clinical data (nonperson identifying)	Intervention practices	The University of Melbourne Patron primary care data custodian [39,45]	Generation of audit reports for the participating practices based on primary and secondary measures
Audit data: primary and secondary outcome measures, and time spent on activities	Intervention practices	Collected and submitted by practice staff as part of PDSA^a^ cycles	Triangulation with Patron EMR^b^ data and contribute to the implementation evaluation. Economic evaluation
Written evidence of quality improvement activities (eg, PDSAs activities)	Intervention practices	Written and submitted by practice staff as part of PDSA	Implementation process evaluation: RE-AIM^c^ framework
Activity reports	Intervention practices	Completed and submitted via REDCap form by practice staff	An estimation of staff time spent doing GooD4Mum program–related activities. Economic evaluation
Referrals to *Life!* programs (aggregated)	Intervention practices	Diabetes Victoria^d^	To triangulate with *Life!* referrals noted in the EMR
FHT^e^ usage data	Intervention practices	The University of Melbourne FHT administrators	Implementation evaluation: Usage of the FHT platform
*Qualitative interview transcripts* with Practice Champions (required) and patients (optional)	Intervention practices	Monash University GooD4Mum researcher interviewees	Implementation process evaluation: As per CFIR^f^ and RE-AIM frameworks [53]. Economic evaluation
*Qualitative interview transcripts* with general practice representatives and external stakeholders	Care-as-usual practices, RACGP^g^, Primary Health Networks, implementation staff	Monash University GooD4Mum researcher interviewees	Implementation process evaluation: As per CFIR and RE-AIM frameworks. Economic evaluation
Online learning workshop recordings and evaluation surveys	Intervention practice staff	The University of Melbourne recordings (Zoom [Zoom Communications, Inc] and Qualtrics survey software [Qualtrics LLC])	Implementation process evaluation
Contact data and field notes from practice visits	Intervention practices	The University of Melbourne Client Management Software	Implementation process evaluation

a PDSA: Plan-Do-Study-Act.

b EMR: electronic medical record.

c RE-AIM: Reach, Effectiveness, Adoption, Implementation and Maintenance.

d With permission granted from the Victorian Department of Health, Diabetes Victoria, and The University of Melbourne Human Research Ethics Committee.

e FHT: Future Health Today.

f CFIR: Consolidated Framework for Implementation Research.

g RACGP: Royal Australian College of General Practitioners.

### Analysis and Evaluation

The statistical outcome analysis, qualitative evaluation, and economic evaluation plans are described in the following text. Also refer to Checklist 1.

#### Analysis of Primary and Secondary Outcomes Measures: Analysis of Baseline Variance, Primary and Secondary Outcomes, and Audit Data

##### Analysis of baseline variance, primary and secondary outcomes, and audit data

Baseline demographic characteristics of intervention and care-as-usual control practices will be assessed using *t* tests and chi-square tests as appropriate, with the practice audit run charts and EMR data used to report on primary and secondary outcome measures, including changes in screening rates and other measures. Repeated-measures ANOVA will be used to analyze primary and secondary end points. Cluster-specific parametric analysis of summary measures estimated for each practice and individual-level parametric analysis adjusted for clustering at practice level will be performed [66]. Multivariable analyses of follow-up in the study arms will be adjusted for practice-level clustering, with relevant covariates using generalized estimating equations (via Poisson or negative binomial family) for counts (eg, number of participants who completed blood glucose testing). A per-protocol set will be used for a sensitivity analysis. Run charts based on Patron EMR data will report the change in measures over the 21 months of data collection and ANOVA will explore change over time.

### Implementation Evaluation and Qualitative Analysis

The CFIR [51,52,67] and the RE-AIM framework [46,53-55,68,69] guide the implementation evaluation. It is through this evaluation that secondary objectives 2, 3, and 5 are met. CFIR is a determinant framework that supports systematic examination of contextual influences on implementation across five domains: (1) Intervention or Innovation characteristics, (2) Outer settings (eg, Primary Health Networks [PHNs] [48], GP Colleges, policies and reporting, and partner or other stakeholders), (3) Inner settings (general practices), (4) End users (clinicians, practice staff, and patients’ knowledge and beliefs about the intervention), and (5) Implementation process (including IT support staff, clinical liaison, and those involved in planning, executing, reflecting, and evaluating). It provides a framework for systematically identifying and understanding system-level factors that influence the uptake of innovation in primary health care settings.

**Figure 2.**
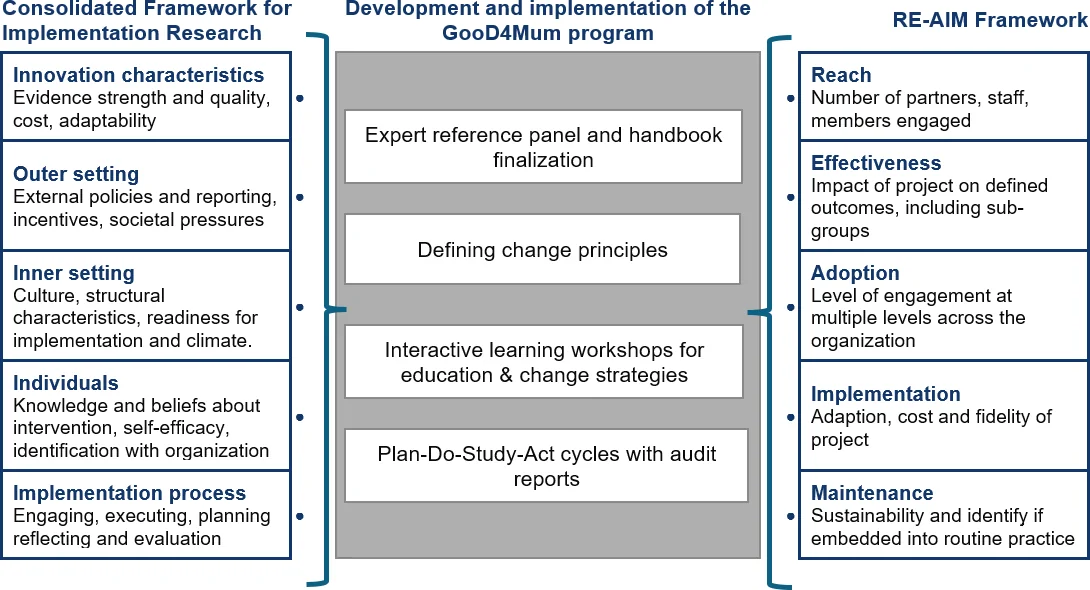
The Consolidated Framework for Implementation Research and the RE-AIM evaluation framework, applied to the evaluation of “GooD4Mum.” RE-AIM: Reach, Effectiveness, Adoption, Implementation and Maintenance.

RE-AIM provides a framework to assess the reach, effectiveness, adoption, implementation, and maintenance of GooD4Mum in the clinical setting. These pragmatic implementation science frameworks enable understanding of factors that influence implementation in complex settings (Figure 2). Together, they provide a theory-driven approach to evaluating dynamic change that helps to describe and explain key mechanisms that either facilitate or inhibit the implementation of new or existing processes. See Multimedia Appendix 6 for more on the outcome measures and data related to the application of the RE-AIM framework and how both qualitative and quantitative data sources (Table 3) will integrate to provide rich data on implementation and the RE-AIM dimensions.

Aligned with the CFIR and RE-AIM frameworks, semistructured 15‐ to 30-minute interviews with representatives from the intervention practices and other settings are conducted by experienced qualitative interviewers at the commencement, midpoint, and conclusion of the 18-month trial (Multimedia Appendix 2). Clinician interviews at care-as-usual control practices occur near the conclusion of the intervention to provide comparative data. Additionally, interviews with other relevant stakeholders, including members of the program implementation and IT support team, and organizational decision makers (eg, from PHNs, general practice colleges, and leaders in GDM management and diabetes prevention) enable a broader perspective on the trial’s execution and outcomes. The short interview duration is in respect for participants’ time constraints. Interviews are audiotaped and transcribed by the research team.

The qualitative analysis was designed to directly complement the study aim and objectives by exploring factors influencing the implementation of GooD4Mum, with a particular focus on the experiences and perspectives of Practice Champions. This included identifying organizational, contextual, and individual-level barriers and facilitators that may influence the primary and secondary outcomes, such as screening completion, BMI recording, referrals, and risk factor documentation in routine care. As summarized in Table 3, multiple sources of data are used in the evaluation, including practice visit field notes made by the GooD4Mum Clinical Liaison, evaluation surveys gathered after each online learning workshop, FHT usage data, and brief reports on PDSAs provided by intervention practice staff.

Deidentified transcripts and other data (Table 3) will be thematically and deductively mapped to relevant CFIR domains and constructs through iterative team discussion. Coding of data and development of models will be assisted by NVivo (Lumivero) analytical software [70]. Coding decisions will be reviewed collaboratively to ensure consistency, with refinement of the codebook as required. Analytic memos will document emerging insights and analytic decisions.

These qualitative findings will provide explanatory context for the quantitative analyses, which assess changes in the proportions of patients completing recommended screening, having key clinical measures recorded, and receiving appropriate referrals, as well as secondary outcomes related to screening requests, new diagnoses, and risk factor recording. Reflexivity will be addressed through regular team-based reflection on researchers’ disciplinary backgrounds, assumptions, and experiences, supported by reflexive notes throughout data collection and analysis [71]. In keeping with qualitative research principles, trustworthiness will be enhanced through data triangulation (credibility), maintenance of an audit trail documenting analytic decisions (dependability and confirmability), and detailed description of the study context and participants to support assessment of transferability [72].

### Cost Consequence Analysis

Cost consequence analysis will be used to compare the implementation and intervention costs and outcomes of the GooD4Mum program with care-as-usual [56]. This evaluation will enable secondary objective 4 to be met and determine the most efficient way to provide the program if implemented at other sites across Australia. A cost consequence analysis approach was selected in preference to a conventional cost-effectiveness analysis because the primary outcomes of this study do not comprise a single summary health end point (such as diabetes incidence). As such, it is not appropriate to aggregate results into a single cost-effectiveness ratio. Instead, the cost consequence analysis will present disaggregated costs alongside a range of relevant outcomes, providing a comprehensive assessment of the resources required and the effects achieved by the intervention compared with care-as-usual. It will identify the costs and outcomes of the implementation process (ie, all costs of implementation during the trial time period and outcomes related to the implementation) and determine the costs and outcomes of the intervention to general practice services in the real-world (ie, costs to general practices associated with the GooD4Mum program during the trial time period and outcomes related to the intervention).

The costs incurred by intervention practices, gathered from EMR data, activity reports, and interviews, will be compared with the real-world comparator “control” practice data and interview data from the care-as-usual practices. For the *implementation process*, outcomes are aligned with the RE-AIM framework [53]. Direct and indirect costs will include implementation staff and practice staff time participating in GooD4Mum activities and associated consumable costs. Outcomes will be presented as total cost to achieve implementation. For the *intervention*, outcomes will relate to the primary and secondary outcome measures of the trial and professional practice improvements assessed via changes in processes of care, adherence to clinical standards, and quality of intervention delivery. Costs will include the following: (1) cost of providing health services to patients in the target population (eg, health service provision and utilization), (2) maintenance of practice-based GDM patient recall register or other direct costs of involvement in GooD4Mum activities, and (3) staff time participating in GooD4Mum activities including identifying, contacting, and following up patients in the recall register (eg, opportunity cost associated with work loss time).

For the control comparator practices, we use GP, PN, and other practice staff time providing health services to the target population and any other costs associated with their identification and recall.

### Ethical Considerations

Ethics approval was gained from The University of Melbourne Human Research Ethics Committee ID 24863 (October 30, 2023). A human research ethics committee–approved waiver of patient consent was in place for the researchers to access deidentified EMR data from intervention and control practices, in accordance with the National Health and Medical Research Council Statement on Ethical Conduct in Human Research [73]. The conduct of the study was compliant with the updated Statement on Ethical Conduct in Human Research 2023 that came into effect on January 1, 2024 [74]. Each practice, control and intervention, consented to data extraction, storage, and use to occur; legal agreements were in place. Both the GRHANITE software and the storage of EMR data incorporated privacy-protecting and data security measures to protect the general practices, personnel, and patients. Patients at *Data for Decisions* partner practices have the option to withdraw their data from being extracted and shared. All EMR data accessed by researchers are deidentified and can be accessed only by the Ethics and Data Governance Committees–approved researchers after they have undertaken mandatory data management and security training. All data access is secured with multifactor authentication; internet access, links to external data disks, and cut-and-paste functions are disabled within the SRE. No raw data can be exported out of the SRE, and only aggregated results can be exported by the external data manager. If the researchers accessing the EMR data become aware of a suspected or actual data breach, they are obliged to notify the Patron Data Steward immediately. This obligation is explicit in Researchers’ *Data Access Acknowledgements* and mandatory compliance training.

For the researchers and participants, we do not anticipate risks or harms more than encountered in everyday life. Participants giving evaluative feedback via survey or interview may feel uncomfortable or frustrated if providing negative details about the intervention or their relationship with clinicians. To reduce the potential for distress, the researchers provided clear explanations about why the activities were being conducted, how the information will be used, and the kinds of evaluative questions that would be asked. Responding to questions is voluntary, and evaluative interviews can be stopped at any point if required.

There is potential that patients without a diagnosis of GDM recorded in the general practice EMR, but with past pathology results that suggest previous gestational diabetes (eg, OGTT levels recorded during pregnancy), might be recalled by their general practice. Some patients may not have informed their GP about their GDM, or not remember they had it, and may feel concerned about the recall. This situation could arise independent of the GooD4Mum program because it is best practice to ensure that women with a history of GDM have blood glucose testing every 1-3 years (as clinically indicated), and it is a recognized role of primary care to enhance disease prevention in a proactive and planned way [75]. So while this situation might produce some concern among patients, managing it is within the scope of practice for primary care clinicians.

On consultation with The University of Melbourne trials unit, this study did not meet the World Health Organization/International Committee of Medical Journal Editors 2008 definition of a clinical trial and was not registered. This study focuses on influencing clinician knowledge and activity rather than prospectively assigning participants to health-related interventions to evaluate patient health outcomes.

## Results

### Overview

**Figure 3.**
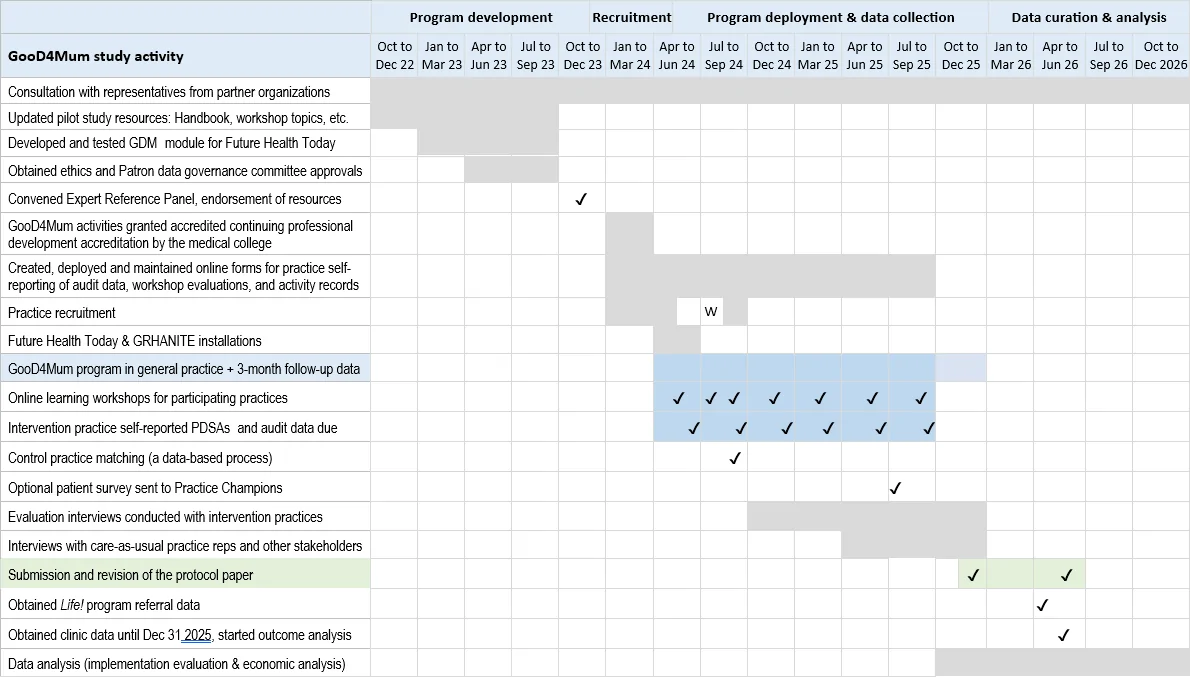
Key stages in GooD4Mum program development, implementation, outcome measurement, and evaluation 2022‐2026 (actual timeline). GDM: gestational diabetes mellitus; PDSA: Plan-Do-Study-Act; W: withdrawal of an intervention practice.

A timeline of key stages of program co-design, development, implementation outcome measurement, and evaluation is outlined in Figure 3. The results of the outcome and cost consequence analysis, and implementation process evaluation will be reported elsewhere. Following are observations on practice recruitment and characteristics and explanation of deviation from the protocol related to practices’ self-report of audit data and researcher contact with staff at allocated control practices. The final tranche of Patron EMR data for outcome evaluation became available in May 2026—results will be published elsewhere.

### Recruitment and Control Practice Matching

Recruitment commenced in November 2023 when ethics committee approval. Potential intervention practices were directly invited by email and telephone; recruitment materials were also sent to Victorian PHNs, GP member organizations, and other relevant health care organizations for broad dissemination in their newsletters. The University of Melbourne database of general practices was used to manage direct contact. Before contact, 42 practices known to have clinical software or server ineligibility were excluded. Fifty-nine practices were emailed invitations and 43 received follow-up phone calls. Practices that did not participate were contacted a mean of 5.0 times (median 3, range 1‐29 emails and phone calls), whereas practices that were onboarded were contacted a mean of 12.7 times (median 13, range 3‐20). In total, 12 practices signed ethics and legal agreements to participate; 3 withdrew prior to commencement citing insufficient resources. Nine practices were inducted to the GooD4Mum program for the April 2024 start. One of the 9 withdrew in August 2024 citing insufficient time for GooD4Mum activities and was replaced in September 2024 by a practice responding to a new broadly disseminated recruitment notice. The final 9 general practices engaged with the program until completion on September 30, 2025.

Controls practices were matched in August or September 2024 according to the protocol; matched pairs are summarized in Table 4. Six pairs were located in 2 major cities (Modified Monash Model measure of remoteness MM1 [47]): Geelong and Melbourne’s inner, west, north-west, north-east, and outer-east regions [76]. The remaining 3 intervention practices were in medium-sized rural towns (MM4), one of which was paired with a control in a large town (MM3). Each pair was located within the same PHN area; 5 of 6 Victorian PHN regions were represented. One rural pair was separated by 41 km—the remaining 8 were a mean distance of 5.4 km apart (range 0.2‐14.4 km).

**Table 4.** GooD4Mum intervention and control practice comparison at baseline (using deidentified electronic medical record data from the Patron primary care data repository)^a^^,^^b^.

	Modified Monash model^c^ (MM1-7)	SEIFA^d^ decile	RACGP’s^e^ ‘active patient’ count	RACGP’s active patient count, female	GP^f^ count (Patron)	*Practice self-reported.* No. GPs and (FTE^g^)	FHT^h^ cohort 1: diagnosed GDM^i^	FHT cohort 2: possible GDM	FHT cohort 3: blood glucose testing >12 months
Intervention 1	1	8	7779	4645	20	15 (9.5)	129	12	94
Control 1	1	1	8355	4465	19	Unavailable	69	10	40
Intervention 2	1	9	1718	1009	4	3 (2.1)	41	5	27
Control 2	1	8	3269	1938	9	Unavailable	48	3	28
Intervention 3	4	2	7666	1938	21	15 (6.8)	108	11	48
Control 3	4	2	2831	4274	10	Unavailable	42	0	22
Intervention 4	1	9	7254	1470	11	9 (6.0)	202	5	147
Control 4	1	9	7955	3718	10	Unavailable	48	6	40
Intervention 5	1	6	2887	4388	6	5 (2.5)	31	6	13
Control 5	1	6	3888	1782	11	Unavailable	58	3	30
Intervention 6	4	4	7451	2163	16	14 (5.5)	78	20	55
Control 6	4	4	4302	4070	10	Unavailable	34	5	27
Intervention 7	4	3	9093	2259	21	11 (9)	93	1	61
Control 7	3	3	7004	4877	10	Unavailable	35	18	26
Intervention 8	1	7	9833	3747	11	11 (4)	25	19	14
Control 8	1	5	7983	5589	11	Unavailable	70	11	29
Intervention 9	1	5	3831	4581	12	9 (5)	119	1	77
Control 9	*1*	*5*	5011	2831	12	Unavailable	81	4	56
Intervention 10	1	10	2989	1707	5	3 (2.6)	N/A^j^, withdrawn	N/A, withdrawn	N/A, withdrawn
Control 10	1	8	4978	2542	6	Unavailable	N/A, withdrawn	N/A, withdrawn	N/A, withdrawn

a From this table, only MMM, SEIFA, and patient and GP counts were reviewed when matching control practices with intervention sites. These data were extracted from the clinical record data (Patron). Patron enables retrospective data with matched dates. Note that FHT cohort numbers are based on all patients marked in the EMR as “active,” that is, the RACGP definition of an active patient was not used (see below). Self-reported numbers of GPs and their FTE were submitted by intervention practices in May and June 2024.

b PHN code: Each pair of intervention and control practice share the same PHN area. To protect practice anonymity, the PHN codes are not provided. There are 6 PHNs in the state of Victoria; 5 are represented in GooD4Mum. The practice that withdrew in August 2024 (Intervention 10) was in the sixth PHN area.

c MMM: Modified Monash Model is a geographical classification system used by the Australian Government to categorize locations by remoteness and town size. 1=major city, 2=regional center, 3=large rural town, 4=medium rural town...7=very remote community [47].

d SEIFA: Socio-Economic Indexes for Areas. Decile 1=most disadvantaged, Decile 10=most advantaged [49].

e RACGP's definition of an active patient: A patient who has attended the practice or service 3 or more times in the past 2 years [35].

f GP: general practitioner.

g FTE: full-time-equivalent.

h FHT: *Future Health Today*.

i GDM: gestational diabetes mellitus.

j N/A: not applicable.

The self-reported number of GPs was generally lower than the administrative count obtained from practice records and equal in 1 instance (GP counts in the EMR are known to be inaccurate when administrative data are not updated regularly). The mean self-reported full-time-equivalent across the 9 intervention practices was 5.6 GPs.

Aligning with the FHT cohorts and outcome measures (Table 2), Table 4 also shows baseline counts of patients with diagnosed GDM, possible GDM, and diagnosed GDM but no record of blood glucose testing in the last 12 months. The cohort counts represent patients flagged as “active” by the practice and, unlike the RACGP’s definition of an “active patient” (attended the practice 3 or more times in the past 2 years [35]), can include people who have not visited the practice for years.

### Self-Submission of Data and Protocol Deviation

As per the protocol, all intervention practices self-submitted clinic information soon after commencement. In May-June 2024, 6 of 9 Practice Champions submitted baseline audit data (Table 1)—the time taken and number to collect the data are summarized in Table 5: a mean of 1.5 people per practice took a mean of 118 minutes to compile and submit the data (range 60-240 minutes).

**Table 5.** Number of people and approximate time taken to gather audit measures at baseline and end of first GooD4Mum activity period.

Intervention practice, n	Baseline		End of first activity period	
	People	Minutes	People	Minutes
1	2	50	Nil^a^	Nil
2	Nil	Nil	Nil	Nil
3	1	240	1	90
4	2	120	Nil	Nil
5	1	60	1	15
6	2	120	Nil	Nil
7	Nil	Nil	Nil	Nil
8	1	120	1	40
9	Nil	Nil	Nil	Nil

a Did not complete the activity.

Intervention site 10 withdrew in August 2024 citing participation being too great a time burden. In response and noting disinclination from other practices to capture and submit audit data, the chief investigators amended the protocol making submission of end-of-activity period audit data optional. Intervention site 10 was offered to remain in the program using the FHT GDM module without requirement to complete any further formal PDSA activities, but they declined.

Participants’ review of audit data is integral to the “study” component of PDSA cycles. As shown in Table 5, time to collect audit data after the first activity cycle fell to between one-quarter and one-third of the time taken at baseline, reflecting an initial learning curve in locating the requested information. Once submission of audit data was optional, no further submissions were made by any practices. This meant that the summaries of previously submitted audit data that were accessible to practices via their REDCap portal were not populated. Reports based on Patron data were instead provided to the practices for their review of audit measures benchmarked alongside the intervention practice average, but due to the cost of accessing Patron data, just 2 reports benchmarked against the average outcome measure across the 9 practices were provided to Practice Champions (May and September 2025).

In March 2025, Patron EMR data (intervention and control sites) for the period April to December 2024 were provided by the Patron data custodian to approved researchers. In April 2025, individual reports were sent to practices with monthly audit measure summaries and graphs alongside a median measure of all intervention practices for benchmarking. The trends were discussed at the next online learning workshop. In August 2025, the second tranche of Patron data was obtained (April 2024 to June 2025), and a second report was circulated to practices in September 2025, with results presented at the final online learning workshop. The final tranche of Patron EMR data for outcome analysis, to December 31, 2025, was made available in the GooD4Mum SRE in May 2026, delayed due to technical issues in the data processing pipeline (independent to the GooD4Mum research team).

### Contacting Control Group Practices and Protocol Deviation

In August 2025, the lead of the *Data for Decisions* program (not part of GooD4Mum) withdrew the approval granted by the research ethics committee and the independent *Patron Data Governance Committee* for members of the research team to contact control practices to request participation in comparative interviews and access to aggregated referral data from the *Life!* program administrators. Although using and contacting data-only controls is novel, it falls within the scope of *Data for Decisions* partner practices’ data-sharing arrangements, which specify that the independent *Patron Data Governance Committee* reviews all requests to use Patron data and that being a *Data for Decisions* partner includes invitations to take part in research.

With subsequent ethics committee approval, the protocol was amended to recruit clinicians from any practice in Victoria providing care-as-usual by circulating a request for Expressions of Interest through channels used to recruit participating intervention practices. The thank you incentive was increased from Aus $50 to Aus $200 to encourage participation and 5 interviews were conducted in November 2025. Analysis of stakeholder and participant interviews will be published with the results of the process and implementation evaluation.

## Discussion

### Preliminary Findings

This protocol describes a pragmatic, clinical practice–level QIC-based program to optimize post-GDM care in general practice, augmented by point-of-care clinical decision support (FHT [41,42]) and privacy-preserving EMR extraction (GRHANITE [40,45]), to improve systematic identification, recall, screening, and referral. Detailed outcome, economic, and implementation results will be reported separately; however, implementation observations that affect their interpretation are already apparent. For example, manual audit data submission by practice staff (a component of the PDSA cycles) proved burdensome (and once optional was not completed), use of matched data-only controls was feasible and provides rich quantitative data, and access restrictions and limited interest from control or care-as-usual practices will affect the qualitative practice comparison.

GooD4Mum builds on prior primary care prevention initiatives that demonstrated feasibility and sustained cardiometabolic risk reductions using QIC methods. The GOAL (Lahti [26]) and Greater Green Triangle [27] studies showed that QIC-informed approaches can produce sustained reductions in risk factors associated with T2DM, and Diabetes Victoria’s *Life!* [30] program has demonstrated efficacy of structured lifestyle support, with online delivery in *Life! After GDM* intended to overcome postpartum barriers [31]. The original GooD4Mum pilot increased postpartum glycemic screening but highlighted persistent barriers to practice change and maternal uptake of prevention programs [14,19,36].

Unlike earlier approaches that relied on manual case finding or bespoke in-practice delivery of behavioral change programs, the current GooD4Mum program with automated point-of-care decision support and centralized deidentified EMR extraction reduces clinician cognitive and administrative burden through automated recall lists, point-of-care reminders about GDM history and blood glucose testing, and direct links to clinical guidelines, and could increase opportunistic screening. This automation may address limitations noted in the QIC and PDSA literature around practice fidelity and assessor access to outcomes data or documentation and limited scalability of fully manual programs [23,24]. Referral to *Life! After GDM* offers a scalable pathway for prevention support outside the practice, recognizing that general practices may not have capacity for in-house program delivery. The combined CFIR and RE-AIM evaluation approach is designed to produce rich explanatory data about contextual enablers and barriers; this addresses the frequent absence of robust implementation reporting in previous PDSA or QIC initiatives and will inform a more usable implementation toolkit.

### Strengths and Limitations

Key strengths of this study include its pragmatic approach designed for real-world general practice settings and the novel use of matched “data-only” control practices to increase recruitment and evaluation efficiency. The mixed methods (CFIR and RE-AIM) evaluation with cost consequence analysis will provide comprehensive insight into feasibility, acceptability, costs, and mechanisms of effect. The trial was in development during the COVID-19 pandemic when recruitment of general practices was particularly difficult. Using data-only control practices already consented for their EMR data to be used for research purposes meant that only intervention practices needed to be recruited, saving time and enabling limited research funds to be used in the development of the FHT GDM module. This novel approach will be a test case for future studies.

This study is subject to several methodological limitations. The non–randomized design and self-selection of intervention practices introduce potential selection bias. The allocation of controls matched on geographic characteristics means that both intervention and control populations are more likely to be affected by the potentially confounding effects of other local programs that may target diabetes prevention. For the updated program, referral to *Life!* is recommended rather than providing bespoke information in general practice. While referral is quantifiable, its measurement presents problems. *Life*! program referral counts captured in extracted EMR data cannot be reliably linked to *Life!* administrative data at the individual level.

### Implications for Practice, Policy, and Future Research

If the forthcoming outcome analyses demonstrate improved screening, risk recording, and referrals, GooD4Mum could support a scalable model in which general practice uses automated identification and point-of-care decision support to manage post-GDM risk, complemented by referral to accessible diabetes and chronic disease prevention programs. For policymakers and funders, the economic findings will be important when deciding whether investment in enabling infrastructure (software licenses, data governance, implementation coaching, and support) offers sufficient value for wider rollout.

### Conclusions

GooD4Mum proposes a novel quality improvement approach in primary care to address known barriers and optimize primary care for people after GDM. The implementation and economic and outcome evaluations alongside the trial are designed to generate insights into quality improvement processes and strategies to generate evidence to develop a scalable implementation toolkit. Pending these analyses, GooD4Mum has the potential to strengthen primary care contributions to prevention of type 2 diabetes and other chronic diseases after GDM by improving automated identification and systematic processes and pathways for recall, screening, and referral to improve health.

## Supplementary material

10.2196/89207 Multimedia Appendix 1 Data fields from Patron primary care data repository.

10.2196/89207 Multimedia Appendix 2 GooD4Mum interview guideline—evaluation.

10.2196/89207 Multimedia Appendix 3 GooD4Mum patient survey.

10.2196/89207 Multimedia Appendix 4 Summary of GooD4Mum change principles and change ideas.

10.2196/89207 Multimedia Appendix 5 GooD4Mum learning workshop example evaluation survey.

10.2196/89207 Multimedia Appendix 6 GooD4Mum—evaluation outcome measures: RE-AIM (Reach, Effectiveness, Adoption, Implementation and Maintenance) framework.

10.2196/89207 Checklist 1 SPIRIT checklist.

10.2196/89207 Checklist 2 SQUIRE 2.0 checklist.

## Abbreviations

CFIR: Consolidated Framework for Implementation Research
EMR: electronic medical record
FHT: Future Health Today
GDM: gestational diabetes mellitus
GOAL: Good Ageing in Lahti Region
GP: general practitioner
HbA1c: hemoglobin A_1c_ (glycated hemoglobin)
OGTT: oral glucose tolerance test
PDSA: Plan-Do-Study-Act
PHN: Primary Health Network
PN: practice nurse
QIC: Quality Improvement Collaborative
RACGP: Royal Australian College of General Practitioners
RE-AIM: Reach, Effectiveness, Adoption, Implementation and Maintenance
SPIRIT: Standard Protocol Items Recommendations for Interventional Trials
SQUIRE: Standards for Quality Improvement Reporting Excellence
SRE: Secure Research Environment
T2DM: type 2 diabetes mellitus
