# Supplementary material for: Improving Proactive Primary Health Care After Gestational Diabetes: Protocol for Implementation and Evaluation of a Quality Improvement Collaborative Program in General Practice (GooD4Mum) and Baseline Practice Characteristics
Source: JMIR Res Protoc. 2026 Aug 28;15:e89207. doi: 10.2196/89207 (PMC13524220; doi:10.2196/89207)
Supplement: Supplementary file 1 [file resprot-v15-e89207-s001.pdf]

Multimedia Appendix 1

GooD4Mum study: De-identified EMR data fields for analysis and control practice matching

Ethics and Data Governance Committee approved data fields from the primary care data repository, EMR data from intervention and control practices

- **Practice unique identifier**
- **Patient ID; Patient details** (Year and month of birth, Gender, ethnicity, Aboriginal or Torres Strait Islander (ATSI)
- **Patient clinical information** (Observations and measurements – e.g. weight, height, body mass index (BMI), blood pressure, waist measurement)
- **Family History** (e.g. information about family history of diabetes)
- **Medical History** (Condition name / description - diagnosis (looking for indicators of gestational or Type 2 diabetes, pregnancy) flag of active or inactive condition, Age at diagnosis, Diagnosis date)
- **Pregnancies**
- **Encounter characteristics** (Visit date, Visit type, Reason code, Reason for visit, visit start time, visit end time)
- **Medicare Benefits Schedule (MBS) items numbers** including any specifically related to diabetes, glycaemic screening, antenatal and postnatal visits, 40-49 and 45-49 year old health checks, etc [1]
- **Medications** (looking for indications of gestational or Type 2 diabetes – current and past medications) - Date first prescribed, Date last prescribed, Medication prescribed details, Medication name, strength, quantity, dose, frequency, Reason for prescription, Long-term or short-term prescription, Age at prescription, Pharmaceutical Benefits Scheme (PBS) item number [2]
- **Investigations / pathology results** (looking for indicators of gestational or Type 2 diabetes, pregnancy); Test name (looking especially for all blood glucose testing including HbA1c, Oral Glucose Tolerance Test, Glucose Challenge Test, cholesterol) - Request date, Collected date, Report date, Result value
- **Modifiable risk factors** (smoking status, exercise, cholesterol)
- **Documents out** (referrals) – noting that contents of documents are not normally visible in the Patron dataset, but subject is (e.g. Referral to Life! [3,4])
- Any **additional flags or fields** identified by the Patron data administrator needed to provide context for the above listed fields

Fields also used for 'data control' practice matching

- Postcode of practice
- MMM classification (Modified Monash Model for measuring rurality [5])
- SEIFA code for the practice (Socio-Economic Indexes for Areas [6])
- Information on whether a practice crosses a state or territory boundary
- Active patient count (flagged as active and using RACGP active patient definition [7])*
- Active Gender Female count (as previous)
- Active Gender Not Recorded (for quality control – checking)
- Active Worker Type (multiple fields – PMs, PNs, GPs, diabetes educators, others)
- Primary Health Network (PHN) area code [8]

*Practices have different administrative capacity to archive patients and clinicians which can lead to inactive patients and clinicians being counted as active. Comparing 'RACGP active' patients (minimum of three clinic visits recorded in two years [7]) is a more robust comparator than using the EMR embedded flag for active vs inactive/archived patients.

See references next page

References

1. Australian Government Department of Health, Diability and Ageing,. MBS Online: Medicare Benefits Schedule. Canberra: Commonwealth of Australia; 2026 [cited 2026 18 May]; Available from: https://www.mbsonline.gov.au/.

2. Australian Government Department of Health, Diability and Ageing,. Pharmaceutical Benefits Scheme (PBS). Canberra: Commonwealth of Australia; 2026 [cited 2026 18 May]; Available from: https://www.pbs.gov.au/pbs/home.

3. Diabetes Victoria. The Life! program. Diabetes Victoria; 2024 [cited 2024 26 November]; Available from: https://lifeprogram.org.au/.

4. Diabetes Victoria. Life! After Gestational Diabetes. Diabetes Victoria; 2023 [cited 2024 22 November]; Available from: https://lifeprogram.org.au/life-program-gestational-diabetes/.

5. Australian Government Department of Health, Diability and Ageing,. Modified Monash Model. Canberra: Australian Government Department of Health, Diability and Ageing; 2026 [cited 2026 18 May]; Available from: https://www.health.gov.au/topics/rural-health-workforce/classifications/mmm.

6. Australian Bureau of Statistics. Socio-Economic Indexes for Areas. Canberra2025 [cited 2025 23 May]; Available from: https://www.abs.gov.au/websitedbs/censushome.nsf/home/seifa.

7. RACGP. Standards for general practices, 5th edition. East Melbourne: The Royal Australian College of General Practitioners; 2023 [cited 27 November 2024]. Available from: https://www.racgp.org.au/running-a-practice/practice-standards/standards-5th-edition/.

8. Australian Government Department of Health, Diability and Ageing,. Primary Health Networks. Canberra: Commonwealth of Australia; 2026 [cited 2026 18 May]; Available from: https://www.health.gov.au/our-work/phn.
