# Supplementary material for: Improving Proactive Primary Health Care After Gestational Diabetes: Protocol for Implementation and Evaluation of a Quality Improvement Collaborative Program in General Practice (GooD4Mum) and Baseline Practice Characteristics
Source: JMIR Res Protoc. 2026 Aug 28;15:e89207. doi: 10.2196/89207 (PMC13524220; doi:10.2196/89207)
Supplement: Supplementary file 2 [file resprot-v15-e89207-s002.pdf]

Interview Guide for GooD4Mum practice champions

Purpose: To explore organizational context, implementation progress, and perceptions across different stages of the study (Commencement, Mid-point, Completion).

Section 1: Background & Context

1. Why and who decided to implement GooD4Mums in your clinic?
2. What are your clinic's key priorities? Where does diabetes prevention fit?
3. Are there other diabetes prevention programs? How do they compare to GooD4Mums?

Section 2: Implementation Experience

4. How is your practice progressing with GooD4Mums implementation?
5. How confident are you in: Utilizing software (Future Health Today), Care coordination (prompts, reminders, bookings), Completing Plan-Act-Do cycles (PDSA)

Section 3: Organizational Readiness

6. Does your infrastructure (size, staff, space) support implementation?
7. What resources are essential for success (funding, supplies)?
8. How well does GooD4Mums fit with existing workflows?

Section 4: Culture & Engagement

9. How does organizational culture influence implementation?
10. Is there a strong need for GooD4Mums in your setting?
11. What are staff attitudes toward PDSA cycles and the online workshops?

Section 5: Support & Communication

12. Beyond the handbook/workshops, what other resources do staff receive?
13. How do you rate GooD4Mums support materials and training?
14. How do internal communication processes help or hinder implementation?

Section 6: Roles & Goals

15. Who is involved in implementation and what are their roles?
16. What goals have been set for GooD4Mums implementation?

Section 7: Challenges & Adaptations

17. What challenges have you faced during implementation?
18. Have you adapted GooD4Mums for your setting? What changes were made and why?

Section 8: Final Reflections

19. Any additional feedback or comments?

Usage Across Timepoints:

- Commencement: Focus on Sections 1–4 (context, readiness, expectations).
- Mid-point: Emphasize Sections 2, 5, 6, and emerging challenges.
- Completion: Prioritize Sections 6–8 (adaptations, outcomes, reflections).

Interview Questions: Care as usual practices / Practice Staff

1. **Current Practice**
   - How does your practice support women with a history of gestational diabetes (GDM)?
   - Approximate numbers: women screened for GDM; women completing postpartum OGTT; women with prior GDM seen in practice.
2. **Guideline Awareness and Use**
   - What is your understanding of diabetes prevention guidelines for women with GDM?
   - How does current practice align with these (e.g., RACGP Red Book use)?
3. **Perceived Importance**
   - How important are these guidelines/issues to your practice?
4. **Training and Resources**
   - What training, resources, or preparation do staff receive?
5. **Care delivery and coordination**
   - How is diabetes prevention care organised in your practice?
     - Identifying at-risk women
     - Use of NDSS register (effectiveness)
     - Recall and booking systems
     - Follow-up screening
     - Lifestyle support/referrals (e.g., Life Program; frequency and effectiveness)
   - How is care coordination managed (e.g., electronic reminders, systems)?
6. **Process Improvement**
   - Are changes needed to improve management and outcomes?
7. **Enablers and barriers to improved care**
   - What are key enablers (e.g., infrastructure, staffing, resources)?
   - What are key barriers?
8. **Program Interest**
   - Would your practice be interested in a program to reduce type 2 diabetes risk in women with prior GDM?
9. **Additional Comments**
   - Any other observations?

Interview Questions: Project Team / Stakeholders

1. Innovation Domain

- What is your understanding of the GooD4Mum intervention?

2. Individual Domain (Role)

- What is your role in quality improvement and/or diabetes/chronic disease prevention?
- How does GooD4Mum align with your responsibilities or organisational policies?
- Who in your organisation is best placed to engage with this type of QI activity?

3. Outer Setting (Alignment, Policy Context)

- How does GooD4Mum align with regional priorities and current initiatives?
- What external policies, supports, or barriers may influence implementation?

4. Implementation (Barriers and Enablers)

- What factors may enable or hinder practice/staff engagement?
- Implementation Process (Communication/Teaming)
- How would you prefer to receive updates and outcomes from this work?

5. Adoption and Sustainment

    - Is GooD4Mum sustainable long-term and scalable to other settings?

6. Any additional observations?
