# Supplementary material for: Improving Proactive Primary Health Care After Gestational Diabetes: Protocol for Implementation and Evaluation of a Quality Improvement Collaborative Program in General Practice (GooD4Mum) and Baseline Practice Characteristics
Source: JMIR Res Protoc. 2026 Aug 28;15:e89207. doi: 10.2196/89207 (PMC13524220; doi:10.2196/89207)
Supplement: Supplementary file 3 [file resprot-v15-e89207-s003.pdf]

Patient Questionnaire - Test

We invite you to complete this survey which will help to assess a program at Medical Centre that aims to support prevention of type 2 diabetes among women who have had gestational diabetes (diabetes during pregnancy).

At the end of the survey, you will be asked if you want to speak to a researcher to give more information (a research interview). Interview places are limited, and people who are interviewed will receive a $30 gift voucher as a thank you.

The trial is being led by researchers at the University of Melbourne and Monash University.

The Project Information Statement has more information. Please download and read it. You can keep it for your records.

[Attachment: "G4M_InformationStatement-Consumers.pdf"]

Please note that by submitting this questionnaire you agree to the following...

I understand that:

this research is funded by the National Health and Medical Research Council. The details of the project are on the information statement that I can download by clicking the link above. this survey is to help evaluate a general practice program that aims to limit the development of type 2 diabetes among people who have had gestational diabetes. no information about me has been given to the researchers by the clinic. my responses will be anonymous and combined with anonymous responses from other women from the GP clinic, then the combined, non-identifiable statistical data will be used for research purposes and may be used for quality improvement purposes by the GP clinic staff. when I submit the survey, it will go to researchers at the Department of General Practice & Primary Care at the University of Melbourne. completing this survey is voluntary and because my responses are anonymous, I cannot withdraw my responses once I have submitted them. the data from this research will be stored at the University of Melbourne and Monash University and will be kept for a period of 15 years after last publication (with personal details deleted if I choose to provide any).

I confirm that I am at least 18 years old and consent to the terms above.

◯ Yes
◯ No

Do you know or have you ever suspected that you had gestational diabetes (high blood sugar during pregnancy)?

◯ Yes
◯ No
◯ Not sure

When were you diagnosed or was it suspected that you had gestational diabetes (high blood sugar during pregnancy)?

◯ 1-2 years ago
◯ 3-5 years ago
◯ More than 5 years ago

Think about any recent visits to your GP clinic:

Did your GP or nurse give you suggestions on how to prevent type 2 diabetes? ◯ Yes ◯ No ◯ Not sure

Did you receive a referral to help with diabetes or chronic disease prevention?	◯ Yes ◯ No ◯ Not sure
Were you referred to the Life! program run by Diabetes Victoria?	◯ Yes ◯ No ◯ Not sure
Have you previously done the Life! program?	◯ Yes ◯ No ◯ Not sure

Did your doctor or nurse check or talk to you about your... (tick all that apply)

☐ Weight or BMI?
☐ Blood pressure?
☐ Waist measurement?
☐ Physical activity?
☐ Diet?
☐ Smoking?
☐ None of the above

Did your doctor order blood tests for checking... (tick all that apply)

☐ Blood glucose (blood sugar) - includes HbA1c, fasting or random blood glucose or oral glucose tolerance test
☐ Cholesterol?
☐ I was sent for other blood tests, not blood glucose or cholesterol
☐ I was sent for blood tests, but I don't know what for
☐ No blood tests were ordered

When you had your appointment were your needs met?

◯ Entirely met
◯ Partially met
◯ Not met

Do you recall receiving a message from your GP in the past 18 months asking you to book a consultation for a health assessment, blood glucose / sugar testing, or diabetes prevention?

◯ Yes
◯ No
◯ Not sure

Were you satisfied with the way you were asked by the GP practice to come in for the appointment?

◯ Satisfied
◯ Neither satisfied nor dissatisfied (neutral)
◯ Dissatisfied

When I got the message I... (please tick one answer)

◯ Booked a consultation and I have had it
◯ Booked a consultation and I am waiting to have it
◯ Didn't book a consultation because I was too busy
◯ Didn't book because I don't think I need a consultation
◯ Didn't book because I go to a different GP now
◯ Didn't book because of the cost of seeing a GP
◯ Didn't book for another reason (optional to state the reason):______

What is your age group?

◯ 18-29 years
◯ 30-39 years
◯ 40-49 years
◯ 50-59 years
◯ 60-69 years
◯ 70+
◯ I prefer not to say

Do you speak a language other than English at home?

◯ Yes
◯ No
◯ Prefer not to say

You can write any extra comments here:

The researchers invite you to be interviewed to provide more information.

- If you agree to be contacted by the researchers, please leave your name and contact details after you submit the survey.
- The brief interview will take 15-20 minute via phone and you will be provided with a $30 gift card to reimburse you for your time.
- Your personal details will NOT be stored with your questionnaire which is anonymous.

Optional Contact Details

Please leave your name and contact details if you would like the researchers to contact you:

- so you can be interviewed about your experiences after gestational diabetes, OR
- so you can get more information.

The brief interview will take 15-20 minute via phone and you will be provided with a $30 gift card to thank you for your time.

Your personal details will NOT be stored with your questionnaire which is anonymous. And your personal details will be deleted when no longer needed.

You can also find more information here: https://mchri.org.au/good4mum/

Thank you!

You can get more information by downloading the Information Statement.

[Attachment: "G4M_WP7_PLS-Consumers.pdf"]

Please choose as many as apply:
(You can just click on Submit if you don't want to respond to this section.)

☐ Please contact me for a 15-20 minute interview (discussion with a researcher)
☐ Please send me more information about the research or the interview process
☐ Please send me a summary of results from the overall study when available
☐ I don't want to be contacted

Name:

____

Email:

____

Phone:

____
