# Supplementary material for: Improving Proactive Primary Health Care After Gestational Diabetes: Protocol for Implementation and Evaluation of a Quality Improvement Collaborative Program in General Practice (GooD4Mum) and Baseline Practice Characteristics
Source: JMIR Res Protoc. 2026 Aug 28;15:e89207. doi: 10.2196/89207 (PMC13524220; doi:10.2196/89207)
Supplement: Supplementary file 4 [file resprot-v15-e89207-s004.pdf]

Multimedia Appendix 4. Summary of GooD4Mum change principles and change ideas

1. Build and maintain the team

i. Engage, communicate with and develop the team.
ii. Set realistic goals.
iii. Reflect on and review activities and progress.

2. Understand your GDM population

iv. Understand gestational diabetes and how it is defined in the Future Health Today (FHT) registers.[1]
v. Establish systems to review and maintain the GDM registers.
vi. Develop systems to improve and maintain data quality and data privacy in the practice, with specific focus on screening for diabetes.

3. Provide systematic and proactive care

vii. Establish an appropriate care pathway, using the evidence-based guidelines, to implement agreed risk reduction strategies for women with a history of gestational diabetes.
viii. Utilise a multidisciplinary team to facilitate reliable care delivery.
ix. Establish and engage with proactive electronic/digital recall and reminder arrangements.
x. Use digital clinical support systems and protocols to support consistent care delivery.

4. Implement self-management principles and provide support

xi. Establish clear definitions of self-management and what 'self-management support' involves.
xii. Draw on tools to implement self-management support towards behaviour change to empower women to better understand and manage their risk of developing diabetes.
xiii. Organise internal and external resources to provide patient-centred self-management support.

5. Provide coordinated and integrated care

xiv. Analyse the patient journey and from their perspective, redesign (if necessary) care pathways and referral process
xv. Identify and engage local organisations and community to support diabetes prevention.
xvi. Communicate with providers, as appropriate, e.g. using My Health Record, with the person's consent.

1. Future Health Today - Clinical decision support for primary care. Melbourne: The University of Melbourne; 2026 [cited 2026 11 May]; Available from: https://medicine.unimelb.edu.au/school-structure/general-practice-and-primary-care/research/research-groups/data-driven-quality-improvement2/themes/future-health-today.
