# Supplementary material for: Improving Proactive Primary Health Care After Gestational Diabetes: Protocol for Implementation and Evaluation of a Quality Improvement Collaborative Program in General Practice (GooD4Mum) and Baseline Practice Characteristics
Source: JMIR Res Protoc. 2026 Aug 28;15:e89207. doi: 10.2196/89207 (PMC13524220; doi:10.2196/89207)
Supplement: Supplementary file 5 [file resprot-v15-e89207-s005.pdf]

THE UNIVERSITY OF MELBOURNE

Multimedia Appendix 5

Example learning workshop evaluation survey

Default Question Block

GOOD4MUM
DIABETES SCREENING AND PREVENTION

GooD4Mum - Learning Workshop 2 - Evaluation Survey

RACGP Activity No. 591984

Your clinic name (optional)

Your name (optional)

Position title (optional)

RACGP Number (optional)

For each of the learning outcomes for this workshop, please rate to what degree they were met:

	Entirely met	Partially met	Not met
To know the impact of GDM, current screening and prevention recommendations.	○	○	○
To identify what you can do in general practice to prevent the development of type 2 diabetes after GDM.	○	○	○
To understand the Life! After GDM program and how to refer to it.	○	○	○

Did you find the workshop relevant to your practice?

	Entirely relevant	Partially relevant	Not relevant
It was...	○	○	○

Please rate to what degree this CPD activity met your expectations about:

	Entirely met	Partially met	Not met
CONTENT: current, contemporary, evidence-based and relevant to general practice (You may add comments)	○	○	○
DELIVERY: engaging / interactive, with opportunity to ask questions and feedback.	○	○	○
PRESENTERS / FACILITATORS: Engaging, qualified / skilled and knowledgeable	○	○	○

Are you likely to recommend GooD4Mum quality improvement activities to a colleague?

○ Yes

○ No

Are you likely to change anything in your practice as a result of today's workshop?

(You can tell us about this in the text box)

- ○ [ ] Yes
- ○ [ ] No

Do you think that your practice's GooD4Mum activities, in the past few months, have contributed to addressing diabetes risk in patients with a history of GDM?

(You can tell us in what way in the text box)

- ○ [ ] Yes
- ○ [ ] No

Were there any barriers or challenges to implementing GooD4Mum-related quality improvement activities this quarter?

- ○ Yes
- ○ No

If there were barriers or challenges, what were they and how did you address them?

(Optional) Approximately how long did you spend this quarter on GooD4Mum related activities?

(Please indicate hours and/or minutes for each activity)

	I did not do this
Reviewing or working with your GDM register(s) in FHT	☐
Reviewing information (not the recall registers)	☐
Planning or doing PDSA activities	☐
Actively recalling women with GDM	☐
Recording audit data	☐
Recording activities for CPD	☐

I did not do this

Other (please state) ☐

If you wish, you can leave other comments or feedback here:

Thank you!

Please submit by clicking on the blue arrow.

Powered by Qualtrics
