# Supplementary material for: Improving Proactive Primary Health Care After Gestational Diabetes: Protocol for Implementation and Evaluation of a Quality Improvement Collaborative Program in General Practice (GooD4Mum) and Baseline Practice Characteristics
Source: JMIR Res Protoc. 2026 Aug 28;15:e89207. doi: 10.2196/89207 (PMC13524220; doi:10.2196/89207)
Supplement: Supplementary file 6 [file resprot-v15-e89207-s006.pdf]

Multimedia Appendix 6

Evaluation outcome measures: RE-AIM Framework

Dimension	Outcome measures	Assessed using…
**R**each	The number of: invited practices that engage in the trial, staff engaged, target population patients screened for diabetes, stakeholders engaged.	Audit data, recruitment notes and stakeholder interviews
**E**ffectiveness	As related to meeting the primary outcome measures (glycaemic screening, recording of BMI, referral to the *Life!* program)	Primary outcome measures and stakeholder interviews.
**A**doption	As related to modifications made by practices / staff to adopt GooD4Mum into practice workflow.	Qualitatively assessed using interview data and using Future Health Today usage data.<br>This outcome will enable understanding of GooD4Mum uptake and ongoing feasibility.
**I**mplementation	Fidelity to GooD4Mum process (recall register creation, recall, quality improvement activities [PDSA cycles], learning workshops) and to evidence-based best practice.	Assessed using mixed methods:<br>▪ observation,<br>▪ workshop recordings and evaluation,<br>▪ interviews,<br>▪ Future Health Today usage data.<br>Review use of the practice register, conduct of diabetes prevention consultations or referrals.
**M**aintenance	Sustained changes retained in the practice.	Assessed at the practice and participant level via:<br>▪ recording processes,<br>▪ administrative records and<br>▪ diabetes prevention consultation attendances, and<br>▪ qualitative data from practice interviews, questionnaires<br>▪ potentially EMR data.
