# Supplementary material for: Improving Proactive Primary Health Care After Gestational Diabetes: Protocol for Implementation and Evaluation of a Quality Improvement Collaborative Program in General Practice (GooD4Mum) and Baseline Practice Characteristics
Source: JMIR Res Protoc. 2026 Aug 28;15:e89207. doi: 10.2196/89207 (PMC13524220; doi:10.2196/89207)
Supplement: Supplementary file 7 [file resprot-v15-e89207-s007.pdf]

The checklist

SPIRIT

STANDARD PROTOCOL ITEMS: RECOMMENDATIONS FOR INTERVENTIONAL TRIALS

SPIRIT

SPIRIT 2013 Checklist: Recommended items to address in a clinical trial protocol and related documents*

Section/item	Item No	Description
**Administrative information**		

GOOD4MUM

DIABETES SCREENING AND PREVENTION

GooD4Mum comprises a suite of eight work packages (WPs). This protocol document focuses on WP5, the general practice-based implementation trial of a type 2 diabetes prevention program for women after gestational diabetes. Other WPs dovetail with WP5 and so are outlined as necessary to provide contextual insight for the GooD4Mum program.

Section/item	Item No	Description
Title	1	***Descriptive title identifying the study design, population, interventions, and, if applicable, trial acronym***<br><br>**Long descriptive title:** GooD4Mum: A pragmatic 18-month implementation trial of quality improvement collaborative activities in general practice to limit onset of type 2 diabetes among women with a history of gestational diabetes versus care as usual<br><br>**Public title:** GooD4Mum: Toward reducing onset of type 2 diabetes among women with previous gestational diabetes, a general practice-based trial of a quality improvement collaborative program<br><br>**Short title:** The GooD4Mum study
Trial registration	2a	***Trial identifier and registry name. If not yet registered, name of intended registry***<br><br>Not required.
	2b	
Protocol version	3	Issue date: 18 August 2023<br>Authors:<br>Rachel Canaway<br>Vincent Versace<br>Douglas Boyle<br>Helena Teede<br>Sharleen O'Reilly

Siew Lim
Emily Callander
Jane Speight
Mark Morgan

Revision chronology: N/A

Funding	4	***Sources and types of financial, material, and other support***

The main funding is via a National Health and Medical Research Council (NHMRC) Partnership Project Grant (APP1191307 – Grant recipient, Monash University). There is also cash and in-kind support from the **Partner Organisations**:
Diabetes Australia funds will be used to support development of practice-based recall registers to be used by trial intervention practices. In kind support for quarterly meeting attendance and representative engagement, project management for data linkage activities and updating Diabetes guidelines based on trial findings.
Royal Australian College of General Practitioners (RACGP), in kind support for marketing and communication support and project meeting attendance.
Diabetes Victoria: In kind support for to fund 100 places on the Life! Program, quarterly meeting attendance and advice.

Note: Australian Government Department of Health funds supported a senior researcher as part of GooD4Mum WP3 and WP4 (supported by NHMRC top up funds) to develop and then disseminate key messages to enhance diabetes screening uptake. Multiple avenues for uptake are being explored including integration of resources into the Life! Program.

Roles and responsibilities	5a	***Names, affiliations, and roles of protocol contributors***

Chief Investigators
Prof Helena Teede (HT), Monash Centre for Health Research Implementation (MCHRI), School of Public Health and Monash Partners Academic Health Sciences Centre, Monash University, Clayton, VIC, 3168, Australia.
Prof Douglas Boyle (DB), Director, HaBIC Research Information Technology Unit (HaBIC R^2), Department of General Practice and Primary Care, The University of Melbourne
Prof Vincent Versace (VV), Deakin Rural Health, School of Medicine, Deakin University
Prof Jane Speight (JS), Australian Centre for Behavioural Research in Diabetes, Deakin University
Dr Siew Lim (SL), Health Systems and Equity, Eastern Health Clinical School, Monash University.
Prof Mark Morgan (MM), Bond University
A/Prof Sharleen O'Reilly (SOR), University College Dublin
Prof Emily Callander (EC), University Technology Sydney / Monash University

Associate Investigators
A/Prof Kevin Mc Namara: School of Medicine, Deakin University
Dr Melinda Morrison (MeM), Diabetes Australia
Justine Cain: Diabetes Australia
Glen Noonan: Diabetes Australia Victoria
Edward Hubby: Australian Government Department of Health

Others
Dr Rachel Canaway (RC), Department of General Practice and Primary Care, The University of Melbourne

Wendy Shepherdley (WS), Department of General Practice and Primary Care, The University of Melbourne
Ms Christine Chidgey (CC), Department of General Practice and Primary Care, The University of Melbourne
Dr Samantha Kozica-Olenski (SK), Implementation Researcher, Monash University

Authors' contributions
HT, SOR, DB, VV and James Dunbar conceived the study. HT, SOR, DB, VV, JS, SL, DF, MM and James Dunbar contributed to the study design. DB, RC, MeM, CC, HT, VV, JS, SL, MM, DF, SOR, will enable implementation. HT, DB, VV, JS, SL, MM, SOR, DF are grant holders. VV provided statistical expertise in clinical trial design and VV will lead the primary statistical analysis. EC will lead the health economic analysis.

5b **Trial Sponsor:** The study appears not to meet the UoM Clinical Trials Policy as it does not prescribe interventions to patients and does not evaluate the effects of the intervention on the health outcomes of the patients but rather evaluates the process change via referral numbers and GP record metrics after providing education and suggested practice changes to the clinics.

5c ***Role of study sponsor and funders, if any, in study design; collection, management, analysis, and interpretation of data; writing of the report; and the decision to submit the report for publication, including whether they will have ultimate authority over any of these activities***

The main funder (NHMRC) does not have a direct role in study design, data collection, analysis, interpretation, writing of reports, presentations or publications and has no authority over these activities. Diabetes Australia have provided funding for the data linkage and recall register development (Work Package 2), is facilitating Work Package 2 linkage, and is advising on appropriate resources and materials for general practices to use around diabetes prevention but does not have authority over the research activities. Diabetes Victoria's Life! program staff have provided advice.

NHMRC: This funding source has no role in the design of this study and will not have any role during its execution, analyses, interpretation of the data, or decision to submit results.

Partner Organisation representatives form a Project Steering Committee. Most of these representatives are named investigators or study associates and have roles in the study execution, analyses, interpretation of the data, or decisions to disseminate results.

5d ***Composition, roles, and responsibilities of the coordinating centre, steering committee, endpoint adjudication committee, data management team, and other individuals or groups overseeing the trial, if applicable (see Item 21a for data monitoring committee)***

GooD4Mum comprises a suite of eight work packages (WPs). This protocol document focuses on WP5, the general practice-based implementation trial of a quality improvement collaborative program for general practice so that GPs can better support women with history of gestational diabetes and thus slow or prevent onset of type 2 diabetes. The health outcomes of women are not assessed. Other WPs dovetail with WP5, for example: WP6 – Economic Appraisal of the intervention and WP7 – evaluation of the intervention. Both WP6 and 7 include

data collection from participating general practices, which are outlined in this protocol. Information about all WPs is included as necessary to provide context.

Study Principal. Chief Investigator A: Prof Helena Teede
Chair of Project Steering Committee. Lead of WP1, WP7 and WP8.
Overall responsibility for:
Management, design and conduct of Good4Mum.
Convening of Steering Committee meetings.
Partnership engagement.
Approval of protocol and revisions.
Approval of study clinic liaison collateral and data collection and reporting forms.
Scientific rigor of the study implementation and evaluation process.
Responsible for publication of study reports, finding translation (implementation toolkit) and dissemination.
Design and capture implementation learnings from the trial based on Consolidated Framework for Implementation Research (CFIR), Normalization Process Theory with applied RE-AIM Framework (Reach, Effectiveness, Adoption, Implementation fidelity and Maintenance) using quantitative and qualitative data collection tools.

Chief Investigator B: Prof James Dunbar – now retired from project
Provided advice on overall study design and implementation of WP5.

Chief Investigator C: Prof Douglas Boyle
Lead of WP2 and WP5, contributing to WP8, and Data Custodian (see below).
WP2 includes stewardship and curation of procured general practice electronic medical record (EMR) and National Diabetes Register data to establish data linkage for analysis.
Oversight of WP5 development and implementation.

Chief Investigator D: A/Prof Vincent Versace
Contribute to WP2, WP5, WP7, WP8.
Sample size, power calculations and statistical analyses.

Chief Investigator E: Prof Jane Speight
Lead of WP3, contribute to WP5 and WP8.
Development of tools and resources to improve risk awareness and screening in women after GDM. Resources handed over to Diabetes Australia who will provide key messaging for use in WP5 resources, based on WP3 findings.

Chief Investigator F: Dr Siew Lim
Contribute to WP7, WP8.
Design and capture implementation learnings from the WP5 implementation trial based on Normalization Process Theory with applied RE-AIM Framework (Reach, Effectiveness, Adoption, Implementation fidelity and Maintenance) using quantitative and qualitative data collection tools.

Chief Investigator G: Prof Mark Morgan
Contribute to WP5, WP8.
Advice on incentivising participation through alignment to GPs' Continuing Professional Development and Quality Improvement schemes.
Influence update of RACGP/Diabetes Australia's national type 2 guidelines based on GooD4Mum findings.
Link GooD4Mum to national primary care and prevention policy with the view to support system level change to introduce new PIP QI (Federally funded Practice Incentive Payments for Quality Improvement).

Chief Investigator H: A/Prof Sharleen O'Reilly
Contribute to WP5, WP7, WP8.

Provide advice on study design and implementation of the intervention based on pilot study experience. Contribute to design and capture of implementation learnings from the implementation trial based on Consolidated Framework for Implementation Research (CFIR), Normalization Process Theory with applied RE-AIM Framework using quantitative and qualitative data collection tools.
A member of the expert Reference Panel (as below)

Chief Investigator I: Dr Dale Ford
Contribute to WP5, WP8.
Principal Clinical Advisor on Quality Improvement Collaborative methodology.
A member of the expert Reference Panel (as below)

Chief Investigator J: A/Prof Emily Callander
Lead of WP6, contribute to WP8.
Design and conduct of economic evaluation of the GooD4Mum program.

Project Steering Committee (quarterly or as required)
Members: All chief investigators, one nominee per partner organisation and RC.
Agreement of final protocol.
Data governance.
Risk management – including direction on COVID-risk management and data management.
Milestone completion.
Budget.
Intra-study data sharing processes and management.
Dissemination, communication and scale up strategy (activities associated with WP8).

Expert Reference Panel for the Quality Improvement Collaborative
Members:
- Quality Improvement Collaborative expert (e.g. Dale Ford or delegate)
- GooD4Mum expert (e.g. Sharleen O'Reilly or delegate)
- Practice Nurse / Credentialled Diabetes Educator (e.g. Wendy Shepherdley)
- Diabetes behaviour and messaging expert (e.g. Jane Speight or delegate)
- Medical specialist who can give insights into hospital practices around GDM management
- Future Health Today expert (e.g. Jo-Anne Manski-Nankervis or delegate)
- RACGP Clinical Guidelines / Quality Care expert (Mark Morgan or delegate)
- GP with interest in GDM and diabetes prevention
- General Practice Manager (from high functioning practice).

Guide content and endorse both the GooD4Mum practice handbook and the topics for the webinar learning workshop (seven planned) to complement intervention practices' Plan-Do-Study-Act (PDSA) cycles.

Custodian of GooD4Mum General Practice EMR and Diabetes Registrant Data: Prof Dougie Boyle
Ensure only approved users access data.
Ensure data are used only for approved purposes.

Project Manager / Implementation Evaluation Manager – (Monash University)
Management of WP 1, 6, 7 and 8.
Coordination across Work Packages, ensure responsibilities of CIA are met.
Secretariat support for Project Steering Committee.
Milestone management
Website and communications

WP2, WP4 and WP5 Project Manager – (University of Melbourne)

Development of recruitment and practice-facing study materials
Obtain required ethics approvals (AIHW and Unimelb),
General practice data governance committee approvals (Patron and Diabetes Register data
Finalisation of study protocol
Provide direction for Data Analysts working on data linkage (WP2)
Coordination between Work Package leads contributing to WP5 (i.e. WP3, 6, 7)
Liaise with FHT team to develop a GDM module appropriate for GooD4Mum
Manage practice recruitment
Provide direction for QI Collaboratives Coordinator
Milestone management
Collection and management of outcomes data

WP4/5 Quality Improvement Collaboratives Coordinator (University of Melbourne)
Responsible for the coordination and delivery of Quality Improvement activities in participating general practices.
Oversee preparation of the GooD4Mum handbook prior to study commencement.
Prepare, coordinate and deliver quarterly learning workshops (online) over the period of the study.
Obtain RACGP Continuing Professional Development (CPD) recognition for the intervention and ensure that CPD requirements are met by participants.
Assist with practice recruitment and liaison.

Participating site Project Champion
In each participating general practice, a GooD4Mum Champion will be identified to be the main contact person for the study team. The Champion will ensure there is continuity within the site around participation in quality improvement activities and feedback sessions and will be responsible for allowing collection of data for audit and evaluative analysis. The Champion may delegate some of this role.

Work Package 5 Team – implementation of the GooD4Mum program (Weekly or as required)
Members: DL, SOR, DB, VV, SL, JS, MM, RC, Collaboratives Coordinator, implementation staff and others with expertise as required.

- GooD4Mum implementation planning, including integration of appropriate outputs from WP2, WP3 and WP4, as necessary, into WP5.
- Gaining Human Research Ethics Committee approval, including for WP6, WP7, WP8.
- Gaining Patron Data Governance Committee approval.
- Drive development of a gestational diabetes module in the quality improvement decisions support software platform Future Health Today (FHT).
- Convene the Expert Reference Panel
- Recruitment of general practices and curation of participant compliance documents (consent forms, MOUs, legal agreements / licences).
- Ongoing liaison with participating practices and self-nominated practice champions.
- Implementation of the GooD4Mum program in recruited general practices.
- Management of trial documentation and master file.

Team meetings for Work Packages 2, 3, 4, 6, 7 and 8 will occur as required. Each team will work to its own objectives and representatives will report to the Project Steering Committee as required.

Work Package 2 – Data linkage
Members: DB, RC, CC, VV and others as required (e.g. Diabetes Australia rep).

Work Package 3 – Key messaging
Members: JS, AL, Diabetes Australia (MeM) and others with expertise as required.
Includes the provision of key messages into WP5 GooD4Mum resources to improve risk awareness and screening in women after GDM.

Work Package 4 – GooD4Mum trial resources
Members: DB, WS, RC, CC with other expertise as required.
Includes the development and finalisation of the GooD4Mum Handbook for general practice and the Future Health Today gestational diabetes module.

Work Package 6 – Economic evaluation
Members: EC with others with expertise as required.
Determine the costs of implementing GooD4Mum with relevance to scale up.

Work Package 7 – Study evaluation / implementation learnings
Members: HT, SL, SOR, VV implementation staff and others with expertise as required.
Application of normalization theory and the RE-AIM Framework (Reach, Effectiveness, Adoption, Implementation fidelity and Maintenance) for study evaluation.
Design data collection methodology, tools and interview schedules.
Capture implementation learnings from the GooD4Mum trial using quantitative and qualitative data collection tools.
Collate implementation learnings and develop an implementation toolkit.

Work Package 8 – Dissemination and scale up
Members: All relevant Cis, led by HT.
Development of a Dissemination and Communication Plan to be iteratively informed by WP7.
GooD4Mum project branding, website, newsletter and annual events.
Recommendations on presentation of endpoint data / findings.
Co-design of program maintenance and scale up plan with partners and key stakeholders.
Communication and program scale up planning.
Communication and dissemination of results.
Implementation of scale up strategies.

Introduction

Background and rationale	6a	***Description of research question and justification for undertaking the trial, including summary of relevant studies (published and unpublished) examining benefits and harms for each intervention.***

Gestational Diabetes Mellitus (GDM) is an increasing health problem affecting up to 18% of pregnancies worldwide. It is one of the strongest predictors of type 2 diabetes mellitus (T2DM).[1,2] Without intervention, weight gained during pregnancy can be retained, increasing the maternal risk of obesity, T2DM, and heart disease; these long-term risks can extend to offspring.[3] The health and socio-economic burden of long-term population management of T2DM is high, so slowing or reducing progression to T2DM can mean improved population health and lower healthcare costs.[2] GooD4Mum seeks to prevent onset of T2DM among women with previous GDM through recall and follow up in general practice.

Existing knowledge providing rationale: Women with a history of GDM have a 10-fold increased risk of developing T2DM compared with those without GDM,

conversion to T2DM is highest within the first five years of giving birth[4] and a third will develop T2DM within 15 years.[5] Lifestyle interventions that include healthy eating and physical activity are known to prevent T2DM in this higher-risk population [6,7]. Diabetes prevention programs have demonstrated efficacy in the general population with significant reductions in T2DM;[8,9] there are, however, key implementation barriers.[10] Reach and engagement in prevention programs is more limited in postpartum women who experience life stage-specific barriers including limited time, infant care needs, tiredness and sleep deprivation, financial constraints from changed work and different priorities.[3,11]

Quality Improvement Collaborative (QIC) methods informed by the Boston Institute for Healthcare Improvement[12-14] have been used to activate behavioural changes at individual, team or system levels.[15] They encourage development of best practices around areas of care with high variation and have the potential to improve outcomes and reduce costs, with the goal of improving patient care at the population level.[16] QICs have been found to be effective in primary and secondary care, able to significantly improve clinical processes and outcomes,[17] including the quality of T2DM care in Australian general practice.[18] The GOAL project in Finland demonstrated feasibility for T2DM prevention using a QIC informed lifestyle intervention in primary care showing positive outcomes sustained for up to three years.[19,20] Our parallel study in Australia, the Greater Green Triangle Diabetes Prevention Study, demonstrated feasibility of a QIC informed intervention in Australian primary care;[21] this was subsequently scaled-up across Victoria (Australian state) as the Diabetes Victoria Life! Program.[22] Our evaluation on the effectiveness of Life! showed sustained T2DM and cardiovascular risk reduction at 12 months.[23] Most participants in Life!, however, were middle to older-aged and the group sessions, which capitalised on peer support, were not appealing to postpartum mothers after gestational diabetes who faced different challenges during their life stage. To address this, our Mothers After Gestational Diabetes in Australia (MAGDA) was developed as a tailored intervention to meet the needs of postpartum mothers with a history of gestational diabetes.[24-26]

While there are many barriers to postpartum diabetes screening and generating lifestyle change for mothers and general practitioners (GPs),[3,27-30] regular diabetes screening helps identify those at higher risk of developing diabetes to enable their earlier engagement and intervention.[31] Both GPs and women with a history of gestational diabetes recognise the role of general practice in providing continuity of care and a coordinating team care approach including education, coaching, diagnosis, monitoring and referrals.[28] We conducted the 12 month GooD4Mum pilot study to introduced QIC methods in general practice specifically aimed to prompt practices to make changes to ensure women with previous GDM were appropriately followed up and supported. This resulted in a significant increase in diabetes screening (43% to 60%), but barriers remained relating to implementing practice change and ensuring T2DM screening and uptake of healthy lifestyle promotion programs among women with past GDM.[15,32] A longer trial and control group were needed to test whether more lasting practice change could be generated. Alongside this we partnered with the Australian Government Department of Health and Diabetes Australia to implement a world first National Gestational Diabetes Register (NGDR) to increase recall, screening and diabetes prevention program uptake via regular reminders sent to women, yet evaluation indicated that screening rates and uptake of lifestyle change prevention programs remained low.[33]

Trial mechanisms: The GooD4Mum program builds on existing research and tested interventions to improve identification, recall and diabetes screening of at-risk women in general practice. The trial is nested within interrelated work packages. The trial introduces Quality Improvement Collaborative (QIC) activities into general

practices, including webinar-based learning sessions and Plan-Do-Study-Act (PDSA) cycles over the 18 month trial, to increase provider capacity to support women with past GDM. While these activities are aimed at preventing or slowing women's progression to T2DM, the measurement of individual health outcomes is not a focus of this study.
The quality improvement and decision support software platform Future Health Today (FHT) will be utilised by intervention practices to create recall registers and facilitate PDSA quality improvement (QI) activities. Outcome measures of intervention practices will be compared to data from comparable control practices that do not receive any intervention but provide care as usual.
As part of the QI activities, audit data from participating practices will be shared during the learning sessions. Practice electronic medical record data with person identifiers removed, from both intervention and control practices, will be used to measure outcomes, also, pending approval, data from Diabetes Victoria's Life! program (to track referrals to Life!). Evaluative data collection will extend for three months after cessation of the 18-month trial. Evaluation of the trial's implementation (WP7), including key facilitators and barriers, and economic appraisal (WP6), will generate real-world knowledge at system and person-levels, to inform guideline-practice gaps and resources for scale-up of the program (WP8).[15]

6b ***Explanation for choice of comparators***

Comparator: 'Real world' de-identified general practice electronic medical record (EMR) data sourced from the Patron data repository (which comprises regular data extraction from over 120 general practices in Victoria) matched to the intervention practices will be used as the 'care as usual' comparator (www.gp.unimelb.edu.au/datafordecisions). Toward the end of the trial the 'control' practices will be invited to participate in WP7 (i.e., qualitative input for 'care as usual' comparison for evaluation).

Objectives 7 ***Specific objectives or hypotheses***

The study aims to assess the effectiveness of audit and quality improvement collaborative activities (implementation strategy), relative to usual practice (implementation comparison) for improving general practice clinicians' (implementation population) provision (implementation outcome, and target of the implementation strategy) of follow up, screening and risk reduction (clinical care) of patients with history of gestational diabetes to ultimately support prevention of onset of type 2 diabetes (intent of the intervention).
(Aim format taken from Wolfenden et al 2021[34].)

The study aims (in brief) to assess the effectiveness of audit and quality improvement collaborative activities, relative to usual practice, for improving general practice clinicians' provision of follow up, screening and risk reduction of patients with a history of gestational diabetes, to ultimately support prevention of type 2 diabetes.

Research hypotheses – WP5
The GooD4Mum QIC intervention practices, compared to control practices will:

- have significantly increased rates of glycaemic screening among women with history of gestational diabetes compared to baseline;
- increase the exposure of women with history of gestational diabetes to type 2 diabetes prevention activities (e.g., Life! program);

- significantly increase the capture of diabetes risk factors and clinical measurements of women with gestational diabetes.

Study objectives

Primary objectives
Comparing GooD4Mum intervention to control general practices, determine whether intervention practice staff / clinician:

- access to a practice-based gestational diabetes patient register facilitates patient identification and improves recall rates of women with history of gestational diabetes.
- engagement with quality improvement activities can improve follow-up of women who have had gestational diabetes, determined through comparisons of rates of glycaemic screening, recording of women's chronic disease risk factors in the patient electronic medical record, and engagement in healthy lifestyle/diabetes prevention activities (measured through referrals to Life! program), and so promote chronic illness risk reduction.

Secondary objectives
In GooD4Mum intervention practices:

- build capacity around Quality Improvement Collaborative methods in primary care and steward best practice management of diabetes prevention for women previously diagnosed with gestational diabetes (WP5).
- determine the costs of implementing the GooD4Mum program, and the most efficient way to provide it if it were implemented at other sites across Australia. (WP6)
- assess reach, effectiveness, adoption, implementation and maintenance (RE-AIM framework) of T2DM screening and prevention activities resulting from GooD4Mum. (WP7).
- identify program implementation enablers and barriers (using NPT and CFIR frameworks) for women, GPs and supporting organisations to inform program scale up (WP7).

Overall, to:

- generate real-world implementation knowledge to inform scale up of the GooD4Mum quality improvement approach for diabetes prevention (based on RE-AIM framework, focused on practice level - WP7).
- develop a co-designed GooD4Mum implementation toolkit to support program scale up (WP7).
- determine guideline-practice gaps for early detection and prevention of Type 2 diabetes among women with previous gestational diabetes and develop resources to address these gaps (e.g., revised RACGP and Diabetes Australia clinical guidelines) (WP8).

Trial design 8 ***Description of trial design including type of trial (eg, parallel group, crossover, factorial, single group), allocation ratio, and framework (eg, superiority, equivalence, noninferiority, exploratory)***

A pragmatic, effectiveness-implementation hybrid study[34] at the general practice level with passive 'data only' control practices allocated in a 1:1 ratio. The study will be conducted at the practice level and individual patient consent is not required except in instances where optional evaluative feedback is sought.

The intervention is based on Quality Improvement Collaborative (QIC) methods informed by the Boston Institute for Healthcare Improvement[12,14] and draws directly from the GooD4Mum pilot,[15] with updates to incorporate technology and processes that were unavailable during the pilot. Quality improvement collaboratives collect data and use that data to provide feedback to clinicians and practice staff, and then encourage implementation of processes to improve patient outcomes.[16] Practices will be provided with the general practice quality improvement technology platform 'Future Health Today' which will have a custom made gestational diabetes module incorporating audit, recall and clinical decision support capabilities and an area for capturing GooD4Mum related QI / Plan-Do-Study-Act activities.[35]

Implementation processes are complex and dynamic reflecting the translation of strategic intentions into everyday practices. Our process and outcome/ implementation evaluation will use GooD4Mum audit data to identify practice and GP engagement with guideline-led postpartum diabetes prevention activities. Practice staff, GPs and a select group of patients from those practices will be interviewed on a regular basis throughout the intervention.

Evaluation of GooD4Mum implementation will be based on Consolidated Framework for Implementation Research (CFIR),[36-38] Normalization Process Theory (NPT)[39,40] and application of the RE-AIM framework (Reach (R), Efficacy (E), Adoption (A), Implementation (I) and Maintenance (M)),[41-44] and will include economic appraisal. Data will be collected by different methods including from general practice electronic medical records (EMR),[45] the Life! program, surveys,[46] Future Health Today usage data, learning workshop (webinar) recordings and/or related notes, Plan-Do-Study-Act outputs and audit data, interviews and focus groups. Results will be triangulated across data sources with data collection occurring at different timepoints for different data sources between baseline and 21 months, comparing intervention and control practices where data from controls are available.

The study will determine changes in practice behaviours, effectiveness of the intervention including rates of patient follow up, recording of patient observations and modifiable risk factors, costs, and barriers and facilitators of participation and practice change. Stakeholders contributing to the evaluation include GPs, practice managers, practice nurses including diabetes educators (if employed) and eligible patients at participating practices. For understanding the contextual backdrop of the trial of GooD4Mum, representatives from Diabetes Australia, Diabetes Victoria, Australian Government Department of Health, Royal Australian College of General Practitioners and others as relevant. Interviews will be undertaken until thematic saturation is reached.

Methods: Participants, interventions, and outcomes

Study setting 9 ***Description of study settings (eg, community clinic, academic hospital) and list of countries where data will be collected. Reference to where list of study sites can be obtained***

The intervention will take place across 10-12 eligible general practices in Australia. The research will be carried out in the state of Victoria, the same environment as the GooD4Mum pilot, and the home state of the Victorian general practice-based research and education network, VicREN (overseen by the University of Melbourne's Department of General Practice and Primary Care), through which recruitment of practices will be undertaken.

Eligibility criteria 10 ***Inclusion and exclusion criteria for participants. If applicable, eligibility criteria for study centres and individuals who will perform the interventions (eg, surgeons, psychotherapists)***

Note, as part of the trial evaluation (WP7), which is separate to its implementation (WP5), eligible clinicians, administrative and management staff and women at participating intervention and control general practices, and representatives from other stakeholder groups, will be invited to participate in qualitative interviews, focus group or feedback surveys.

Eligibility of General Practices

General practices will be eligible for inclusion in the study if they meet all of the inclusion criteria and none of the exclusion criteria.

General Practice Inclusion Criteria – Intervention arm:

- Practice located in Victoria, Australia
- Employs a practice nurse
- At least one GP willing to be actively involved in GooD4Mum
- The practice contributes data, or is willing to contribute data, to the Patron repository of de-identified primary care data via GRHANITE® data extraction software.
- The practice is willing to have Future Health Today (FHT) software installed on practice computers.
- Willing to voluntarily participate in the quality improvement collaborative program including undertaking quality improvement activities related to diabetes prevention among mothers.
- Willing to have a representative sign all necessary Legal Agreements to confirm their commitment to the project and to formalise incentive payment.
- Willing to nominate a GooD4Mum Practice Champion.

Technical eligibility:

- Has a PC with Windows 10 on which GRHANITE® (research data extraction tool) can be installed. This PC cannot be the practice management system server.
- Uses Medical Director (not Helix) or Best Practice clinical electronic medical record (EMR) software (GRHANITE® and FHT interfaces with these software systems).
- Does not share a server with another practice (because of the complexity of isolating data from a single practice within a server shared across multiple practices).

General Practice Exclusion Criteria – Intervention arm

- Located outside of Victoria, Australia
- Practice concurrently involved in another trial or program focused on improving care for women with previous gestational diabetes.
- Practice does not meet eligibility criteria.

Technical ineligibility (related to installation of GRHANITE® and FHT)

- Apple Mac only workstations.
- Ineligible clinical practice software including cloud-based (i.e. uses software other than Medical Director or Best Practice).
- Practice intends to change their clinical software during the time of the trial.
- Ineligible PC specifications (e.g. not running Windows 10).
- Shares a server with another practice.

General Practice Inclusion Criteria – Control arm:

- Located in Victoria, Australia

- Is a partner in the Data for Decisions www.gp.unimelb.edu.au/datafordecisions, an e-research network (and therefore meets all of the technical eligibility criteria as per intervention arm practices) i.e., has GRHANITE® installed and provides up to date data to The University of Melbourne for research purposes.
- Does not share a server with another practice.
- Matched to intervention arm practices as per criteria at 16a.

Note: previous participation in the GooD4Mum pilot or other diabetes prevention programs does not preclude involvement or intervention or control practices. The pilot ceased in December 2015, which is considered a sufficient wash out period.

Staff from control practices will be invited to contribute to the GooD4Mum evaluation (WP7). Their participation is voluntary.

Eligibility of General Practitioners (GPs), practice nurses (PNs), Diabetes Educators

Inclusion criteria

- Willing to sign a Consent Form;

Exclusion criteria

- There are no specific exclusion criteria.

Eligibility of Practice Managers (PMs)

Practice Manager inclusion criteria

We will include Managers who:

- Sign a consent form if taking part in interviews and/or focus groups or are the nominated Practice Champion at intervention practices.

Practice Manager exclusion criteria

- Practice managers who are also participating GPs will not be further recruited to participate as a practice manager.

Any staff member at **intervention** arm practices may be the GooD4Mum **Practice Champion** (often the Practice Manager). The Practice Champion will have a standalone consent form to complete.

Eligibility of Participant Women

As part of the trial evaluation (WP7), eligible women at participating general practices will be invited to complete a questionnaire and participate in qualitative interviews. This is separate to women being recalled for general practice consultations as a part of routine care.

Women's inclusion criteria

- Women with a past history of or suspected history of Gestational Diabetes Mellitus (GDM)

Women's exclusion criteria

- Women/girls under 18 years of age.
- Difficulty communicating in English.
- Established Type 1 or Type 2 diabetes.

Eligibility of other stakeholders / partners

As part of the trial evaluation (WP7), representatives from 'outer settings' Primary Health Networks (PHNs), Diabetes Australia, Diabetes Victoria, Australian Government Department of Health, Royal Australian College of General Practitioners (RACGP) and other relevant organisations will be interviewed.

Inclusion:

- Will sign a consent form.

Exclusion:

- Individuals new to the stakeholder organisation unable to provide sufficient contextual insight into the impact (potential or actual) of GooD4Mum.

EMR data inclusions/exclusion criteria:

- Where data (extracted using GRHANITE®) are used for outcome measures, de-identified EMR data from intervention and matched control practices will be accessed from the University of Melbourne's Patron primary care data repository.

- EMR data minimisation principles are in place. The researchers access only data relevant to the study as approved by Human Research Ethics and Patron Data Governance committees.

Inclusion:

- Study data period: 1st January 2016 to date at time of data extraction.
- Includes only females aged 12 to 60 years old during the study data period, plus historical data pertaining to the cohort preceding 1 January 2016 on diagnosis, pathology results, pregnancy and other fields that can impact participant eligibility for recall.

Exclusion:

- Data pertaining to men.
- Women outside of the included age range.

Note: There is a waiver of patient consent in place for collection of de-identified patient data at participating practices within WP5 as part of the trial and its evaluation (WP6 and 7).

Interventions 11a ***Interventions for each group with sufficient detail to allow replication, including how and when they will be administered***

See Table. Schedule of data collection activities (interaction with intervention and control practices from recruitment to trial end)

Control practices

- After recruitment of intervention practices, control practices will be selected by matching characteristics as per 16a.
- Control practices provide care as usual.
- At the end of the first year control practices will be invited to participate in *optional* evaluative activities related to WP6 and WP7.
- Control practices remain de-identified to the researchers until such time as they consent to identification. Liaison with the practices before consent is via the Data for Decisions / VicREN Coordinators who have authority to liaise with the practices as part of the Data for Decisions program of work. If a matched control practice agrees to be contacted their details will be passed to the GooD4Mum team for follow-up and formal consent.

Intervention practices

Through participation in GooD4Mum activities, GPs and their participating clinic colleagues will work to:

1. Build and maintain the team

- Engage, communicate with and develop the team.
- Set realistic goals.

- Reflect on and review activities / progress.

2. **Understand the GDM population**
    - Develop a register of women with gestational diabetes (using a GDM module in Future Health Today the platform [35])
    - Develop systems to review and maintain their gestational diabetes register.
    - Develop systems to improve and maintain data quality in their practice, with specific focus on screening for diabetes.

3. **Provide systematic and proactive care**
    - Establish an appropriate care pathway, using evidence-based guidelines, to implement agreed risk reduction strategies for women with a history of gestational diabetes.
    - Utilise a multidisciplinary team to facilitate reliable care delivery.
    - Establish and engage with proactive recall and reminder arrangements.
    - Use digital clinical support systems for consistent care delivery.

4. **Implement self-management principles and support women with a history of gestational diabetes**
    - Establish clear definitions of self-management and what 'self-management support' involves.
    - Organise resources and referrals to enable woman-centred self-management support.
    - Implement a strategy for self-management support to empower women with a history of gestational diabetes to better understand and manage their risk.

5. **Ensure women with a history of GDM receive coordinated and integrated care**
    - Analyse the woman's journey and redesigning where necessary.
    - Engage with relevant stakeholders to ensure appropriate referral pathways and to support development of local diabetes prevention services.
    - Provide integrated care by improving the relationship between primary, secondary and tertiary providers.
    - Communicate with local providers concerning the shared health summary.

Pre-trial (during recruitment)

- **Relationship building:** Gain agreement, communicate importance and strategic vision, build a sense of collaborative community.
- **Practice Champion self-nomination:** Each practice will nominate a GooD4Mum Practice Champion. The Champion will be the primary point of contact for the research team and will drive GooD4Mum activities within the practice.
- **Compliance paperwork:** Authorised person(s) from each practice will complete compliance documents including necessary Legal Agreements and software licences and complete any other necessary compliance documents.

Pre-trial (after recruitment)

- **Installation of GRHANITE® and Future Health Today (FHT):** Practices that do not already have GRHANITE® research data extraction software or FHT

software will have these remotely installed on a practice computer (completed by University of Melbourne HaBIC R^2 development/operations staff). The PC with GRHANITE® installed should be left switched on to allow data extraction to occur overnight. The PC with FHT installed should be left switched on overnight to allow FHT to update by interacting with the clinical management software (Best Practice or Medical Director). EMR data used by FHT does not leave the practice.
- **Data extraction:** Via GRAHNITE®, de-identified data is passively extracted from the practice EMR by University of Melbourne. All data are held in the secure Patron data enclave with approved data being provided to the researchers in line with an approved schedule. From the general practice perspective, once GRHANITE® is installed this is a passive activity not requiring practice staff input (providing that they PC is left switched on and clinical software package is not changed.).

During the trial

- **GooD4Mum Project Handbook**: Participating staff will be provided with the GooD4Mum Handbook (endorsed by the Expert Reference Panel [see 5d]). The Handbook is a resource for practices to guide their quality improvement initiatives and Plan-Do-Study-Act cycles; it will augment information outlined during the Learning Workshops.
- **Audit of practice data:** Practices have access to audit data, mainly via their Future Health Today dashboard and will be asked to track their progress on an Audit data run sheet which will assist in Plan-Do-Study-Act (PDSA) cycles. Prior to each learning workshop, participants will be expected to have reviewed information available to them on audit measures within Future Health Today and any other material provided them, and provided a copy of their audit results to the GooD4Mum team.
    - The audit measures, which are also to be used as outcome measures, are outlined at #12.
- **Learning Workshops (online)**: The GooD4Mum Project Champion or their delegate and any other interested staff or clinicians attend a series of 60 minute learning workshops, the frequency of which will be quarterly.
    - The learning workshops are facilitated by the Collaboratives Coordinator / Clinical Liaison and may include invited experts.
    - Each workshop will have a topic with a specific aim which will be endorsed by the Expert Reference Panel.
    - The learning workshops are designed to be motivational and structured to include:
        - an education session on a pertinent topic,
        - group sharing of Plan-Do-Study-Act (PDSA) cycle experiences.
        - discussion on:
            - differences in aggregated audit data related to core GooD4Mum measures (see Outcomes #12),
            - goals and steps to reach for during each Plan-Do-Study-Act quality improvement cycle,
            - overcoming obstacles to improvement.
        - Additionally, Practice Champions (or their delegate) with outstanding audit measures and PDSA cycles will be coached on their implementation strategies.
    - With permission the learning workshops will be recorded and notes taken to be used for trial evaluation.
    - **Evaluative feedback** from the learning sessions will be shared with the WP6 and WP7 teams for implementation and economic evaluation as well as being used by the Collaboratives Coordinator/WP5 team for immediate program improvements.

- **Recall of women with history of GDM:** Practices will be instructed in how to create and maintain a GDM recall register from their EMR data within Future Health Today.
  - Practice staff may elect to undertake case finding of women for the register using non-automated means such as text searching of medical notes. Relevant women can be added to the recall register within Future Health Today if they are not already listed.
  - Practice staff will be expected to recall women on their recall register, as relevant to offer glycaemic screening, diabetes prevention information and/or referral to the diabetes prevention Life! program (run by Diabetes Victoria).
- **Plan-Do-Study-Act (PDSA) cycles:** In between each quarterly workshop, practice participants undertake and submit to the Collaboratives Coordinator evidence of their GooD4Mum-related PDSA activities. The evidence will be forwarded to the relevant professional association for clinician continuing professional development (CPD) hours. The PDSA activities can be captured in a template in the Future Health Today software or they may be captured in another manner agreed between practice staff and researchers.
  - Activities are at the practice level.
  - Suggestions for the activity cycles will be in the GooD4Mum Handbook and made explicit as part of the learning workshops.
  - The GooD4Mum project will use 'stretch' goals to provide a focus for the PDSA activity each practice undertakes. Stretch goals will not be easy to achieve so will require significant improvement work to be met. However, they are aspirational targets with **any steps towards meeting the goal considered an improvement.** Goals are used to guide and motivate improvement. The Expert Reference Panel will refine / endorse the project goals and they will be provided in the GooD4Mum Handbook. The stretch goals might be:
  - Within 21 months of trial commencement, 100% of active patients with history of GDM are recalled for blood glucose screening (if clinically due for screening)
  - Within 21 months of trial commencement, 100% of active patients with history of GDM are recalled for a consultation discussing diabetes prevention (includes referral to Life!).
    - i.e. 21 months allows for a 3 month data collection period after trial cessation – this data can be captured by Patron data rather than through additional GP audit.

As part of the quality improvement methodology, it is up to the practice to determine how they implement use of the recall register, learnings from the webinars, what their activity periods (PDSA cycles) will cover and how many activities they will undertake per PDSA cycle.

For trial evaluation activities that will include interaction with practice staff, patients and other stakeholders, for WP 5, 6, 7, see Data collection methods (18a)

11b *Criteria for discontinuing or modifying allocated interventions for a given trial participant (e.g., drug dose change in response to harms, participant request, or improving/worsening disease)*

In accordance with national and international ethical research guidelines, a participant (general practice, contributing GP, practice nurse, practice manager, patient or other stakeholder) has the right to withdraw their consent without explanation at any time upon their request or the request of their legally

acceptable representative. When a general practice withdraws from the study, they are requested to do so in writing. The reasons (if known) for withdrawal shall be recorded by the investigators.

A study investigator can withdraw a general practice from the study if the practice:
- is in violation of the protocol;
- requires early discontinuation for any reason.

Withdrawing completely from the study may depend on the timing of the request. The general practice participant compliance paperwork (e.g. practice legal agreement, participant information and consent forms) explain the right of practices or individuals to withdraw and how this can occur. If practice or individual participants' data or other information have already been used for the purposes of the GooD4Mum trial, or if the results have been published at the time of withdrawal, then it will not be possible to rescind this participant's data from completed analyses.

Notices at general practices will alert patients that they can withdraw from having the de-identified data from their EMR from being extracted by GHRANITE®. This withdrawal is subject to the limitation of data already extracted and used in the research. Clinical software

11c ***Strategies to improve adherence to intervention protocols, and any procedures for monitoring adherence (e.g., drug tablet return, laboratory tests)***

Control arm practices do not adhere to an intervention.

GooD4Mum research or practice liaison staff will be in regular interaction with intervention arm practices providing support. QIC activities are designed to be motivational and to encourage adherence. Practices will be provided with a GooD4Mum Project Handbook, which is a reference for all participating clinicians and other staff. The Handbook aims to support practice staff to undertake Quality Improvement activities to support women with history of GDM. The Handbook will address challenges in the delivery of improvements for patients. It will include evidence-based guidance, resources / references, practical examples and guiding questions.
The regular learning webinars, facilitated by the GooD4Mum team, are designed to be motivational.
The intervention is designed to activate behavioural changes at individual and team levels to improve practice's clinical processes and outcomes.

The QI activities contribute to CPD points for participating practitioners, if not completed, partial allocation of CPD points or hours can be obtained.

Practices that have not shared their PDSA cycle activity will be given some reminders. If a practice seeks additional information from the research team around completion of an activity, that information/support will be provided.

Practice staff / clinicians can seek additional support from the Future Health Today team on the use of Future Health Today software or additional support from the GooD4Mum team.

Adherence assessments: Attendance at learning workshops will be monitored, and GooD4Mum Practice Champions will be reminded to implement their Plan-Do-Study-Act cycles.

11d ***Relevant concomitant care and interventions that are permitted or prohibited during the trial.***

The practice-based gestational diabetes registers will exclude women who have already progressed to a diagnosis of type 2 diabetes. As a pragmatic trial, with regard to women on the gestational diabetes registers, there are no prohibitions on concomitant care nor engagement in other interventions.
With regard to participating intervention and control arm practices, their recruitment is contingent on them meeting the eligibility criteria (#10).

Outcomes 12 ***Primary, secondary, and other outcomes, including the specific measurement variable (eg, systolic blood pressure), analysis metric (eg, change from baseline, final value, time to event), method of aggregation (eg, median, proportion), and time point for each outcome. Explanation of the clinical relevance of chosen efficacy and harm outcomes is strongly recommended.***

Timepoints for measurement (EMR data extraction):

1. Timepoint zero (T0) = Baseline
2. T1 to T6 = quarterly over the 18 month trial
3. Follow-up (F1) = data capture at 3 months post trial cessation (21 months).

Primary outcome measure (WP5):

Proportion/number of **women with history of GDM** in **intervention** and **control practices** who:

1. have recorded T2DM-screening test (oral glucose tolerance test (OGTT) and/or fasting blood glucose (FBG) and/or HbA1c) in the EMR undertaken within the 21-month timeframe of the data collection (total number of screens). And also, the number of women who are tested:
      i. within x months post-partum (e.g 3,6,9,12).
      ii. within x years of their last known blood glucose test when pregnancy was >12 months ago (e.g. 1,2,3).
      iii. with no information about their last known blood glucose test.
   b. Data fields – from Patron data:
      i. Diagnosis and/or medication and/or pathology results consistent with gestational diabetes
      ii. Presence of investigation results for any relevant glycaemic screening, including OGTT, FBG, HbA1c
      iii. Date of delivery, estimated date of delivery, MBS item 16407 (Postnatal professional attendance by an obstetrician or general practitioner between 4 and 8 weeks after the birth); other relevant MBS item indicating pregnancy

2. have measurement of weight/height or Body Mass Index (BMI) captured in the EMR:
   a. measured at each data collection timepoint and cumulatively.
   b. Data fields:
      i. Height (to enable BMI calculation) – not required at all timepoints, however, in mothers under 18 years of age, height should be captured yearly to calculate BMI.
      ii. Weight (to enable BMI calculation) – preferably at regular intervals, e.g. 6 monthly.

3. were referred to the Diabetes Victoria Life! Program (includes Life! GDM and regular Life! program).
    a. at each data collection timepoint and cumulatively.
    b. Data fields:
        i. Number of referrals to Life! program. Data to be obtained from Life! program coordinators (pending approval from Victorian Government Department of Health).
        ii. Referral data from Patron where Life! referral is known only if written in referral 'Subject' field (less accurate).

Explanation of the primary measures:
Abnormal blood glucose is a risk factor for onset of type 2 diabetes and cardiovascular disease. Guidelines recommend OGTT at six weeks after delivery and at least every 2-3 years after diagnosis of GDM. It is also recommended before planning a subsequent pregnancy.

Overweight and obesity are risk factors in onset of T2DM and other chronic conditions. BMI measurement was identified in the GooD4Mum pilot as a critical measure to identify high-risk women within practices' GDM recall registers. The routine recording of weight during general practice consultations also instils in women a sense of importance of weight maintenance and can be used as a conversation starter in health coaching.

We would like to capture the provision of diabetes prevention information by practice staff to women with history of GDM, but it is difficult to quantitatively measure this. Referral to Life!, which can be measured in both intervention and control practices (pending data custodian approval) is a quantifiable proxy for the provision of preventive information. The names of control practices will be de-identified unless the practice provides permission for Life! program administrators to provide GooD4Mum researchers with referral data referral data from Life! to be identified. No identifiable patient-level data will be collected.

Secondary outcome measures (WP5):
For **intervention and control practices**, de-identified EMR (Patron) data on **women with a history of GDM.** Changes in:

1. requests for T2DM-screening (OGTT and/or FBG and/or HbA1c) within the 21-month timeframe of the data collection at each data collection timepoint and cumulatively.
    a. Explanation: There may be more requests than results.
2. number of women newly diagnosed with pre-diabetes or type 2 diabetes.
    a. Explanation: Prevention of type 2 diabetes is the aim of GooD4Mum, but if pre=diabetes or diabetes are diagnosed sooner because the blood glucose monitoring is improved, then the woman's outcome can be better.
3. recording of modifiable lifestyle risk factors:
    a. blood pressure,
    b. cholesterol,
    c. waist circumference,
    d. smoking,
    e. physical activity.
        i. Explanation: Routine recording of modifiable risk factors during general practice consultations instils in women a sense of their importance and can be used as a conversation starter in health coaching / diabetes prevention.
4. Distribution of BMI among women on individual general practice GDM registers. This will include review of change in BMI measures calculated as

average percentage change over time, from initial to final measurement within the 21-month data collection period.

a. We do not anticipate reduction in BMI due to GooD4Mum, but we want to understand the distribution of BMI and check whether there is any change.

Intervention practice audit measures (for reporting with study outcomes)
Documented by practice staff by looking at Future Health Today portal, with aggregated de-identified information also available from Patron data via the researchers.

Timepoints for measurement:

- Timepoint zero (T0) = Baseline
- T1 to T6 = quarterly over the 18 month trial
- Follow-up (F1) = data capture at 3 months post trial cessation (21 months).

1. The number of women on the GDM recall register (can include women with suspected GDM based on pathology results or medication taken during pregnancy).

The number of women on each intervention practice GDM recall register who:

2. have a coded diagnosis of GDM in the EMR.
3. have been newly recorded as having type 2 diabetes.
4. have had an OGTT/FBG/HbA1c measurement recorded since the last count date.
5. receive diabetes prevention information or referral (e.g. to Life!, diabetes prevention action plan)
6. gave birth within the previous 12 months.
7. gave birth within the previous 12 months AND have an OGTT or HbA1c measurement recorded within 3 months of delivery.
8. have a new pregnancy recorded.
9. Have recorded weight and height OR BMI.
10. have BMI < 25 *(Note: This is not about achieving this BMI rather showing the distribution of healthy weight and overweight/obesity category women within practices on audit).*
11. Have recorded values of waist circumference and blood pressure.
12. Have recorded values of cholesterol and smoking.
13. Have recorded physical activity.

Descriptive measures – intervention and control practices (WP5):
Patron data.
To describe and give context to the intervention and control cohorts.
Exclude archived / inactive patient records.
Data at timepoints as relevant from baseline (T0) to follow-up (F1)

1. Number of female patients 16-60 years old
2. Number of women per SEIFA (Socio-Economic Indexes for Areas) decile
3. Number of women with one or more pregnancies recorded
4. Age at last pregnancy
    a. (Age and Last Delivery Date or other field indicating pregnancy)
5. The number of women with a coded diagnosis of GDM
6. The number of women who have a coded or free text diagnosis of GDM
7. The number of women with suspected GDM (pathology or medication during pregnancy, but no coded diagnosis)
8. Age when GDM recorded
9. Of those with history of GDM at baseline, ‘Last visit date’ (aggregated by quarter and year).
10. Of those with history of GDM at baseline, visit count 24 months preceding trial and visit counts during trial.

11. Number of women with T2DM recorded (not limited to women with history of GDM)
12. Age when T2DM recorded
13. Number of women, not limited to those with GDM or T2DM diagnosis, with height and weight OR BMI recorded (at baseline and T6)
14. Number of women with history of GDM diagnosis with height and weight OR BMI recorded
15. Number of women flagged as RACGP-Active (three visits to the GP in the last two years) to compare to above counts (1-14)*

Note that descriptive statistic measures may vary, drawing on the approved Patron data fields.

*** Relevance of RACGP Active**
'RACGP active' refers to the RACGP criterion where an active patient is one who has attended the practice three or more times in the past two years.[47] It is not uncommon that research studies, including the GooD4Mum pilot, limit patient participants to those classed as RACGP active. By contrasting the descriptive measures between all patients with active records and those flagged as RACGP active, the impact of the difference in women who may be recalled is made explicit.

Outcomes for economic appraisal
Comparative costs for the general practice - Intervention compared to control practices:

1. GP, nurse and other practice staff time providing health services to women with history of GDM
2. Costs associated with recall of women with history of GDM (e.g. purchase and maintenance of practice based GDM patient register, a proportion of cost of patient recall system),
3. staff time identifying, contacting and following up patients on the recall register.

Comparative costs and outcomes of the implementation process (Intervention practices only).

4. practice staff/clinician time participating in GooD4Mum activities (e.g. learning webinars, PDSA cycles, seeking support to implement GooD4Mum)
5. consumable costs associated with GooD4Mum activities.

Implementation evaluation – WP7
Assessed against the RE-AIM framework. RE-AIM framework (Reach (R), Efficacy (E), Adoption (A), Implementation (I) and Maintenance (M))[41,42]
Capture data at the practice and participant level.

1. **Reach.** Number/ proportion of:
    a. invited practices that engage in the trial
    b. number of staff engaged
    c. number of women identified as at risk and percentage screened
    d. number of stakeholders engaged.

2. **Effectiveness.** As related to meeting the primary outcomes measures (see #12).

3. **Adoption.** As related to modifications made by practices / staff to adopt GooD4Mum into practice workflow. Qualitatively assessed and Future Health Today usage data. Will enable understanding of GooD4Mum uptake and ongoing feasibility.

4. **Implementation.** Fidelity to GooD4Mum process (recall register creation, recall, QI activities [PDSA cycles], learning webinars), fidelity to evidence-based best practice.
    a. Assessed using mixed methods - observation, webinar recording and evaluation, interviews, surveys and Future Health Today usage data. Review use of the practice register, conduct of diabetes prevention consultations or referrals, FHT usage data.

5. **Maintenance.** Sustained changes retained in the practice. Assessed at the practice and participant level via recording processes, administrative records and diabetes prevention consultation attendances, and gathering qualitative data from practice interviews, questionnaires and potentially EMR data.

Participant timeline 13 ***Time schedule of enrolment, interventions (including any run-ins and washouts), assessments, and visits for participants. A schematic diagram is highly recommended***

See Table. *Schedule of data collection activities (interaction with intervention and control practices from recruitment to trial end)*

See Figure. *GooD4Mum: Flow of practice recruitment, contact and data collection*

Sample size 14 ***Estimated number of participants needed to achieve study objectives and how it was determined, including clinical and statistical assumptions supporting any sample size calculations***

Twelve practices will be recruited to the trial with these practices matched, 1:1 with Patron data-based control practices. We expect that each practice will have a variable number of active patients with history of gestational diabetes. We have allowed for the potential loss of 2-3 practices by 18 months (e.g., practice closure, practice withdrawals).

Sample size calculation basis: The primary outcome is screening rates in GDM, identified by Bogdanet et al. as a key variable in the post-GDM Core Outcome Set (COS).[48] The study will be powered to detect a minimum difference of 10% in screening rates between the control and intervention practices. This will be achieved by including **24 general practices (across intervention and control arms)** with an average cluster size of 15 women per practice per annum (total of 300 eligible women). The sample size is based upon a standard deviation of 25% of the number of eligible women in each of the GooD4Mum pilot practices,[15] 5% significance level and 90% power, and accounts for the general practice clustering effect (ICC set at 0.001). This number of practices is required to meet the minimum requirement for reliable analysis of a cluster design.
This sample size with a 10% difference was selected to:

a) enable detection of a difference between intervention practices and control practices which still receive some intervention with the practice register;
b) meet the optimal rules for cluster size and analysis;
c) detect anything over a doubling of the 5% who currently participate in a diabetes prevention program in this population;[32] and
d) align with the percentage involved in prevention assessment in the GooD4Mums pilot.

This sample size also allows individual pre and post assessments of anthropometrics, glycaemic control and validated questionnaire-based outcomes in women engaged through the intervention practices.

Recruitment 15 ***Strategies for achieving adequate participant enrolment to reach target sample size.***

Recruitment will be managed by staff at the University of Melbourne Department of General Practice and Primary Care whose recruitment strategies, guided by VicREN (Victorian primary care practice-based Research and Education Network),[45,49] have been successful as evidenced by recruitment of over 400 practices for 8 RCTs (NHMRC APP1059863, 1023738, 1104241, 1121104, 1064121, 1059863, 1007937, Future Health Today RCT funded by Paul Ramsay Foundation), 140+ general practices to be part of the Data for Decisions e-Practice-based research network (ePBRN), and recruitment of many other general practices to participate in other research or quality improvement projects.

Figure: *GooD4Mum: Flow of practice recruitment, contact and data collection* outlines the recruitment strategy.

Figure: *GooD4Mum, Decision pathways for practice participation* guides recruitment staff to assess practice eligibility for participation

Practices targeted for recruitment will be sent an Expression of Interest, they will be phoned, emailed and/or visited in person.

- Any general practice that meets eligibility criteria and completes and returns all required compliance paperwork, will be enrolled.

The practices targeted may be:

- A current partner in the Data for Decisions / Patron program (www.gp.unimelb.edu.au/datafordecisions) and so are already providing de-identified practice data for research.
    - **Note**: *Existing Data for Decisions practices have already completed the required consent process and legal agreement to cover the provision of data that will otherwise be required in addition to consent and agreement to participate in GooD4Mum.*
- A practice that already has Future Health Today software installed.
- A member of the VicREN primary care practice-based Research and Education Network (www.gp.unimelb.edu.au/vicren).
- Any eligible practice listed in the National Health Service Directory.

Strategies for recruitment:

- Use VicREN's general practice recruitment database and filter for practices with practice nurse.
- Utilise researchers' existing networks.
- Draw on peer-to-peer practice recruitment, which has been shown to be a highly successful strategy for recruiting general practices.[50]
- Medium sized practices will be targeted to maximise efficiency of on the ground engagement.
- Target practices in areas with higher prevalence of GDM as per NDSS Diabetes Map (https://map.ndss.com.au).

Remuneration: It is anticipated that $1000 reimbursement will be paid, in instalments, to intervention practices – as follows:

- $500 at trial establishment, i.e. installation of GRHANITE® and FHT any, completion of compliance documents [consent form, legal agreement(s))
- $500 on trial completion where all deliverables and other requirements have been met to the researchers' reasonable satisfaction.

Thank you payment to Practice Champion

- $200 gift card

- To encourage participation in **WP7** interviews or focus groups, we will seek ethics approval to provide gift cards to participants to thank them for their time. For example, $30 gift card as a thank you for participation. The amount will be determined by the Good4Mum Project Steering Committee depending on available budget.

To encourage retention of GooD4Mum practice liaison staff will be in regular contact with participating practices, troubleshooting and providing support as required and will record all liaison with practices (see Data Management).

Recruitment of other stakeholders (WP7): Implementation research in WP7 will include evaluation of the trial will include collection of quantitative and qualitative data from stakeholders in addition to the participating general practice clinicians and staff to provide a contextual backdrop to the intervention's evaluation. Some of the 'other stakeholders', such as RACGP, Diabetes Victoria, Diabetes Australia are partners in GooD4Mum. The affiliated associate investigators from these organisations will facilitate contact with the appropriate persons in their organisation to provide evaluative feedback for the trial. Other representative stakeholders, such as at Primary Health Networks, will be identified and approached via email invitation and follow up phone call.

Patient feedback (WP7) on their experience of recall and diabetes prevention consultation will be sought via survey with link to the survey supplied by the practice by QR code at the consultation and/or via text message. Women wishing to be contacted to provide additional feedback will be given the option to nominate to do so as one of the survey questions and GooD4Mum staff will contact them using the contact details provided by the woman.
The QR code / survey link will be different for each practice so that the practice of patient origin can be known.

Methods: Assignment of interventions (for controlled trials)

Allocation:

Sequence generation | 16a | ***Method of generating the allocation sequence (eg, computer-generated random numbers), and list of any factors for stratification. To reduce predictability of a random sequence, details of any planned restriction (eg, blocking) should be provided in a separate document that is unavailable to those who enrol participants or assign interventions.***

General practices will be recruited to the intervention arm with a 1:1 allocation to a control practice that is part of the Data for Decisions network of general practice that contributes de-identified EMR data to the Patron data repository. Matching will occur based upon control and intervention practice proximity, size/activity, and depending on feasibility, socioeconomic status as described by SEIFA code and MMM classification (see below). Flexibility in matching will be retained to account for interventions occurring simultaneously in the geographical footprint of the GooD4Mum trial (i.e. interventions that could conceivably contribute to contamination).

Practice matching will draw on the following fields from Patron EMR data:

- Postcode of practice
- MMM classification (Modified Monash Model for measuring rurality)
- SEIFA code for the practice (Socio-Economic Indexes for Areas)
- Information on whether a practice crosses a state or territory boundary
- Active patient count
- Active Gender Female count
- Active Gender Not Recorded (for quality control – checking)
- Active Worker Type (multiple fields – PMs, PNs, GPs, others)
- Primary Health Network (PHN) code

Allocation concealment mechanism	16b	***Mechanism of implementing the allocation sequence (eg, central telephone; sequentially numbered, opaque, sealed envelopes), describing any steps to conceal the sequence until interventions are assigned.***<br><br>Allocation to intervention or control arms will not be concealed to practices or researchers.
Implementation	16c	***Who will generate the allocation sequence, who will enrol participants, and who will assign participants to interventions***<br><br>All recruited practices that fulfil the inclusion criteria, consent to participate (return of compliance paperwork), and provide practice information for randomisation, will be included in the intervention.<br><br>These practices will be matched by the statistician with Data for Decisions practices to act as controls. The actively engaged general practices, when recruited, will know that they are intervention practices, there will be no randomised allocation against the Patron data control practices.
Blinding (masking)	17a	***Who will be blinded after assignment to interventions (e.g., trial participants, care providers, outcome assessors, data analysts), and how.***<br><br>The trial is unblinded but participating practices will be de-identified in data provided to the GooD4Mum statistician, with a unique practice ID as allocated in the Patron dataset, being used in lieu of practice name.
	17b	***If blinded, circumstances under which unblinding is permissible, and procedure for revealing a participant's allocated intervention during the trial.***<br><br>Not applicable.

Methods: Data collection, management, and analysis

Data collection methods	18a	***Plans for assessment and collection of outcome, baseline, and other trial data, including any related processes to promote data quality (eg, duplicate measurements, training of assessors) and a description of study instruments (eg, questionnaires, laboratory tests) along with their reliability and validity, if known. Reference to where data collection forms can be found, if not in the protocol.***<br><br>Sources of data:<br>See *Table. Schedule of data collection activities (interaction with intervention and control practices from recruitment to trial end).* Data sources and their collection:

A number of data collection methods will be used for understanding contextual factors, assess outcomes and evaluating implementation.

Quantitative data collection

- **De-identified EMR data** from participating general practice, Intervention (I) and Control (C).
    - Collected via GRHANITE® at baseline and quarterly until timepoint F1 = 3 months after cessation of trial.
    - Used to measure or describe:
        - Primary outcomes (1, 2 and part of 3) (I/C)
        - Secondary outcomes (I/C)
        - Audit measures – to triangulate with data provided by practice staff (I)
        - General practice population (descriptive measures (I/C)
        - WP6 Economic appraisal (I/C)
        - WP7 RE-AIM framework: Effectiveness, Maintenance (follow up for check of sustained changes) (I/C)
- **Audit data (I)**
    - Generated and collected quarterly by general practice participants during PDSA cycles, drawing from FHT and their EMR – provided to Good4Mum team to be used as outcome data.

Qualitative / Implementation evaluation data collection

All qualitative data collection relates to the Consolidated Framework for Implementation Research (CFIR) which has five domains across i) outer settings (PHN, policy, RACGP, PHN and other partners/ stakeholders at the start and end of the interventions) ii) inner settings (practices) iii) end user/ workforce fit (practice and PHN staff) and iv) evidence-based interventions (guideline driven Good4mums pilot informed practice level intervention) and v) the implementation process.
In outcome/implementation evaluation we apply the Reach, Efficacy, Adoption, Implementation and Maintenance (RE-AIM) framework.

- **Online learning workshop evaluation forms** (I)
    - for WP6 & WP7 – economic appraisal and implementation evaluation.
- **Online learning workshop recordings and/or notes** (I)
    - Recordings and notes of the webinar discussion with permission of participant.
- **PDSA cycle activity logs** (I)
    - Submitted by practice staff for GooD4Mum team to check and submit to professional associations as evidence of task completion for continuing professional development points.
    - Used by GooD4Mum team as data to assess practice adoption and implementation of GooD4Mum program (as part of RE-AIM framework)
- **Future Health Today usage data** (I)
    - For evaluation of adoption, implementation and maintenance.
    - Aggregate usage data that shows:
        - aggregate age and gender data for each practice.
        - how the software has been interacted with, for example, how many times the point of care tool activated on a given day,
        - data relating to recommendations, including total count of eligible patients with an active recommendation and summary counts for any cohort (recall register) created – this will triangulate with some audit measures.
- **Normalization Process Theory (NoMAD) survey** is a validated, structured instrument.[39,51] (I)

- Completed by GPs, PNs or PMs at intervention practices.
- Collection will depend on the capacity of the individuals at each practice to contribute. At minimum, GooD4Mum Practice Champion and one other staff participant should complete.
- To be deployed 4 times between baseline and end of trial, but there may be some variation to accommodate the practice staff.

- **Questionnaires – women 18 years and over on the recall list** (I)
  - A link to an anonymous e-questionnaire to be sent by participating practice staff to women on their recall register after recall or appointment without recall. Sending the survey will need to fit with practice workflow and should be sent every 3-6 months.
  - To determine perspectives on the recall and satisfaction with support provided if the woman had an appointment, or reason for not booking a consultation after recall.
  - Questionnaire respondents can elect to be contacted to provide additional information by phone or virtual interview (online).
  - A de-identified summary of findings will be provided back to the practice for quality improvement purposes.
- **Semi-structured interview or focus group data (I/C)**
  - *All interviews/focus groups will be conducted and analysed by the Monash Implementation research lead trained in qualitative interview and implementation science research, who has no prior relationship with participants.*
  - **Interview data from patients (I/C)**
    - **Intervention practice:** A sub-sample of questionnaire respondents who volunteer to be interviewed. Consistent questions to extend on questionnaire data about satisfaction with recall and support provided. Interviews can occur any time after questionnaire completion, approximately 10-20 minutes.
    - **Consenting control practices**: Invitation sent by practice staff to women at the practice with diagnosed GDM. Collect data on women's perceptions of their support received around GDM. Interviews can occur any time after control practice has been onboarded.
  - **Interviews / focus groups with practice staff / clinicians** (I / C - optional)
    - Using consistent questions to look at factors involved in the Good4Mum implementation process, barriers and enablers, needs and program sustainability.
    - Collection will depend on the capacity of individuals at practices to contribute.
      - baseline (I)
      - in the last second half of the trial from timepoints T4-F1 (I/C)
      - other timepoints should practice staff wish to (optional) (I)
      - upon a practice withdrawing early from the trial (I)
    - ***Note** that Control practices will not be invited to contribute until late in Timepoint 3 (approaching 12 months. Only consenting control practices will be invited to contribute data.*
  - **Interview or focus group data from other stakeholders** (e.g. PHNs, AGDoH, Diabetes Australia)
    - Using consistent questions, similar to those used for the practice staff, to look at factors involved in the

Good4Mum implementation process, barriers and enablers, needs and program sustainability related to GooD4Mum.

- Collection is planned at baseline, mid and end points of the trial.

See below for additional information about data sources and data fields.

EMR data, GRHANITE® and the Patron repository

The University of Melbourne developed GRHANITE® research data collection tool will be installed on a practice computer at each participating practice and will be used to extract de-identified administrative and patient data for compiling gestational diabetes recall registers for intervention practices, assessing the impacts of the intervention compared to care as usual (as per the outcome measures) and for quarterly audit data review.

Data extracted by GRHANITE® used for GooD4Mum are stored in the Patron de-identified primary care data repository within a secure University of Melbourne environment. Good4Mum researchers apply to the Patron Data Government Committee to access Patron data.

The University of Melbourne's ethics approved **Data for Decisions / Patron program** (www.gp.unimelb.edu.au/datafordecisions) provides an established and tested legal, ethical, governance, data curation and security framework under which GooD4Mum intervention and control practices share their EMR data (University of Melbourne Human Research Ethics Committee approval ID 1647396). There are currently over 140 general practices mostly in Victoria, contributing de-identified data, via GRHANITE®, to the Patron data repository. These practices comprise the e-practice-based research network that will be engaged for GooD4Mum practice recruitment and control practice matching.

Confidentiality: GRHANITE® facilitates privacy protecting data capture and secure transmission of encrypted data from participating general practices in compliance with Australian privacy legislation. The instance of GRHANITE® installed at a particular general practice computer is programmed so that all fields normally containing patient **or** practitioner identifying information are removed prior to transmission to the Patron data repository. **None** of the following fields are extracted: names; addresses; full dates of birth; practitioner provider, prescriber or other identifiable numbers; Medicare numbers; and most free text fields with over 30 characters (e.g. clinical notes).

Data extracted is of the type routinely collected during the GP-patient encounter, there is no requirement for GPs to collect any new or additional information. The extraction process is designed to ensure usual clinical workflow is not disrupted. Extraction occurs on a weekly basis, normally overnight. University of Melbourne, Department of General Practice (HaBIC R^2) staff remotely **install** GRHANITE®, **manage** (e.g. software updates / upgrades and configurations), **operate** (e.g. perform data extracts), **monitor** and **control** data extraction from the practices in compliance with defined Standard Operating Procedures. GRHANITE® data are de-identified before leaving the practice, but mappings to the identity of patients and practice staff are retained to allow re-identification by practice staff if required. A re-identification key is held by the general practice where the data were captured. The reidentification key is unique to the instance of GRHANITE® that is installed on that specific general practice computer. Neither researchers nor the Patron data technical team have ability to re-identify patients or practitioners / staff without the active involvement of the practice where the data was originally captured.

De-identified patient level data fields from the EMR / Patron

Requested data fields: Practice unique identifier; Patient ID; Patient details (Year and month of birth, Gender, Residential postcode, ethnicity, ATSI); Patient clinical

information (Observations and measurements – e.g. weight, height, BMI, BP, waist measurement); Family History (e.g. information about family history of diabetes); Medical History (Condition name / description - diagnosis (looking for indicators of gestational or Type 2 diabetes, pregnancy) flag of active or inactive condition, Age at diagnosis, Diagnosis date); Pregnancies; Encounter characteristics (Visit date, Visit type, Reason code, Reason for visit, visit start time, visit end time); Medications (looking for indications of gestational or Type 2 diabetes – current and past medications - Date first prescribed, Date last prescribed, Medication prescribed details, Medication name, strength, quantity, dose, frequency, Reason for prescription, Long term or short term prescription, Age at prescription; Investigations / pathology results (looking for indicators of gestational or Type 2 diabetes, pregnancy); Test name (looking especially for blood glucose, HbA1c, Oral Glucose Tolerance Test, Glucose Challenge Test, cholesterol) - Request date, Collected date, Report date, Result value; MBS items numbers including any specifically related to diabetes, gyclaemic screening, antenatal and postnatal visits, 40-49 and 45-49 year old health checks, etc); Modifiable risk factors (smoking status, exercise, cholesterol); Documents out (referrals) – noting that contents of documents are not normally visible in the Patron dataset, but subject is (e.g. Referral to Life!).

Data for practice matching: Once all intervention practices have GRHANITE® installed data can be used to match them to 'data only' control practices. Data fields used to match practices is outlined at 16a.

Data for economic appraisal – WP6

- From Good4Mum team observations and files: staff type and time length of attendance at QIC activities (implementation staff and GP practice staff), QIC consumable costs, purchase and maintenance costs of recall register, number of women in eligible population per practice. (I)
- Webinar (learning workshop) evaluation form: practice staff time associated with recall register (monitoring, contacting, booking patients) (I)
- Audit data run sheet question (I)
- Interview/focus group, questionnaire data. (I/C)
- From de-identified EMR patient data: all MBS item numbers of women in eligible population, encounter characteristics (visit date, visit type, visit length) (I/C)

18b ***Plans to promote participant retention and complete follow-up, including list of any outcome data to be collected for participants who discontinue or deviate from intervention protocols.***

Once a practice is recruited, the GooD4Mum team will make every reasonable effort to retain that practice within the study. They will respond to queries arising from the practice staff/clinicians in a timely manner and provide support when requested. GooD4Mum practice liaison staff will develop relationship with GooD4Mum Practice Champions and support them to support their practice to engage in the study.
Practices can seek additional support from the Future Health Today team on the use of Future Health Today software or additional support from the GooD4Mum team.

If a practice or individual chooses to withdraw, they may do so without giving reasons and without prejudice. As per the Consent form, the GooD4Mum team will ask for a reason for withdrawal for their records, but participants are under no obligation to provide a reason if they do not wish to.
Collected data pertaining to a withdrawing practice or individuals, that has been processed by the GooD4Mum researchers, may be retained if it is not possible to extract it (because of de-identification and aggregation).

Data management 19 ***Plans for data entry, coding, security, and storage, including any related processes to promote data quality (e.g., double data entry; range checks for data values). Reference to where details of data management procedures can be found, if not in the protocol***

The investigators responsible for the various Work Packages (ie. recruitment, development and delivery of learning workshops, generating practice recall registers and reporting audit data, economic appraisal, implementation evaluation) are responsible for ensuring the accuracy, completeness, legibility, and timeliness of the data reported, and, where required, will maintain adequate records of study participant information and consent.

Data Security and Backup
At the University of Melbourne, mechanisms for maintaining data security follow and adhere to the internal:

- Management of Research Data and Records Policy (MPF1242) augmented by resources on research Data Management (Procedures and Guidelines for Management of Research Records and Data), and
- Information Security Policy (MPF1270).

These procedures and guidelines in-turn reference and conform to National and Victorian State regulatory and policy frameworks for privacy and security of data and to other University of Melbourne research regulations.

The various tranches of study data are bounded by governance, ethics and legal agreement on the terms and security measures under which they can be accessed. Data security and backup are discussed alongside each dataset below.

MS Dynamics - Recruitment and practice liaison data
University of Melbourne, Department of General Practice Microsoft Dynamics Client Management Software, especially tailored for general practice research and teaching recruitment placements, will be used to record GooD4Mum related contacts with general practices. A GooD4Mum module will be in use during recruitment, onboarding, and practice liaison throughout the trial. This module will be used to record or captures practice status (invited /in negotiation / confirmed / declined / withdrawn); practice staff names and positions; primary contact; exchanged emails, phone calls and visit contacts/memos; meeting of eligibility criteria; receipt of incoming compliance documents (e.g. return of MOU or legal agreement, consent forms); reasons given for non-participation; general notes / observation; and flags date for follow-up. Access to this database is by licence, is password protected and is tightly controlled, only for VicREN recruiters. The ID of the staff member recording information is recorded. Dynamics is run from a University of Melbourne managed server, the entire system is backed up nightly.

Qualtrics recruitment EOIs: Expressions of interest for recruitment will be collected via the University of Melbourne Qualtrics Research Core within the Learning Management System. Access to data within Qualtrics is user specific and requires entry of unique username and password. Information about the EOI will be transferred into MS Dynamics GooD4Mum project module.

General study, reporting, survey, interview and focus group data from the economic appraisal and implementation evaluation will be stored in restricted access folders on University secure research environments accessible on password protected computers (utilising multi factor authentication) and will be accessible only by the project team. Any paper files will be stored in a locked filing cabinet at the institution of the lead CI responsible for its collection.

A naming convention will be encouraged for storing files associated with the trial/research to ensure ease of location by practice, content and date: Trial_WP#_PracticeID_ParticipantID_Status_Type_Title_Date_Author_Version#

____

Trial: G4M

WP#: Work Package 1 to 8

PracticeID (use if relevant): i.e. Site code = agreed abbreviation of site – including for other stakeholders (e.g. Diabetes Victoria (DA), AGDoH (Australian Government Department of Health)

Participant ID (use if relevant): GP (General Practitioner), PN (Practice Nurse), PM (Practice Manager), Pt (Patient), SH (other Stakeholder)

Status: DRA (Draft), FLD (Filled form), Int (internal only), Rx (Redacted), SS (Superseded), TC (Tracked Changes), X (Obsolete)

Type: Agd (Agenda), Br (Brochure), Frm (Form), GdL (Guideline), Cor (Letter / Email / Other correspondence), Min (Minutes), SOP (Standard Operating Procedure), Tem (Template), WF (Workflow), or other agreed abbreviation for collected data or document type.

Title: Concisely describes the content and function, using abbreviations where possible.

Date: Date that the document was distributed / published, formatted as YYYYMMDD

Version#: Increase in increments of "1" for major updates, and "0.1" for minor alterations.

____

EMR data warehousing: Practice EMR data in the Patron primary care data repository are warehoused entirely within a University of Melbourne controlled environment, on the Melbourne node of the national NeCTAR Cloud e-Research infrastructure facilities, of which The University of Melbourne is the lead agent. The repository is physically located at the University of Melbourne node of NeCTAR. The Patron primary care data repository is actively managed by the University of Melbourne, Department of General Practice HaBIC R^2 team in conjunction with University of Melbourne IT Services. Data storage and data security processes are in line with the long-term and comprehensive experience of the HaBIC R^2 team who have developed and implemented GRHANITE® projects nationally since 2007.
Good4Mum researchers with ethics and Patron Data Governance Committee approval access the GooD4Mum tranche of EMR data via a secure virtual machine (two factor authentication) that is locked to data export. Refer to the *Patron Data Risk and Management Plan*.
EMR data security: Practice EMR data have security processes actively managed by the University of Melbourne, Department of General Practice HaBIC R^2 team, with oversight from the Patron Data Custodian (Head, Department of General practice) and the Patron Data Steward (Professor Dougie Boyle). Mechanisms for maintaining data security follow and adhere to The University of Melbourne Management of Research Data and Records Policy (MPF1242) and the Procedures and Guidelines for Management of Research Records and Data, and The University of Melbourne's Information Security Policy (MPF1270). These procedures and

guidelines in-turn reference and conform to National and Victorian State regulatory and policy frameworks for privacy and security of data and to other University of Melbourne research regulations.
The GooD4Mum VM is protected within firewalls applied to the NeCTAR allocation and as managed by the HaBCI R^2 technical team. Before the Patron technical team (HaBIC R^2) release approved data fields to the GooD4Mum team, they scrutinise the data to ensure de-identification and that the data are in concordance with GooD4Mum ethics approval. Only approved users can access the data and they do so through institutional user account authentication via Australian Access Federation (AFF) authentication. Two-factor authentication is then required to login to the GooD4Mum repository utilising DUO MFA.

Study status reports
Each Work Package Team will, on a regular basis, collate information on missing reporting, forms, issues and seek to resolve them. Issues that cannot be resolved within the team will be escalated, as appropriate, to GooD4Mum CIs and advisors. Each Work Package Team will report to the Project Steering Committee as required.

Statistical methods 20a ***Statistical methods for analysing primary and secondary outcomes. Reference to where other details of the statistical analysis plan can be found, if not in the protocol***

Analysis of baseline and quarterly audit measures
Simple descriptive statistics to report the measures as listed at Outcomes (#12). Denominator = number of women on the practice based gestational diabetes recall register.

Analysis of baseline variance, primary and secondary outcomes
Baseline demographic characteristics of study arms and clusters will be assessed using t-tests and χ2 tests as appropriate, with Practice audit data run charts used to report changes in screening rates in intervention and control practices. Repeated measures analysis of variance will be used to analyse primary and secondary endpoints. Cluster specific parametric analysis of summary measures estimated for each practice and individual level parametric analysis adjusted for clustering at practice level, will be performed.[52] Individual-level univariate analysis will use χ2 test as well as t-tests, adjusted to account for clustering. Multivariate analyses of follow-up in the study arms will be adjusted for practice-level clustering, with relevant covariates using generalized estimating equations. Intention to treat will be applied via a per-protocol set for a sensitivity analysis.

Implementation research – WP7 will be framed by Normalization Process Theory[39] and application of the RE-AIM framework (Reach (R), Efficacy (E), Adoption (A), Implementation (I) and Maintenance (M)).[41,42]
Semi-structured Interviews among each cohort group will be undertaken until thematic saturation is reached.[53,54] Interviews will be recorded and thematic analysis of de-identified data undertaken using NVivo software.[55]
De-identified respondent IDs will be generated. Survey data from practitioners and patients will be analysed using an appropriate statistical software package to generate descriptive statistics. The general practice of respondents' origin will be traceable by researchers, but data pertaining to any specific practice will be kept anonymised in any reporting of results. Patient and practitioner/staff anonymity will be maintained with all recordings deidentified.
Results will be triangulated across data sources.

Economic analysis – WP6

A detailed economic analysis protocol will be published separately, detailing analysis approach. Briefly, a **cost consequence analysis** will be conducted. This will identify the costs and outcomes of the implementation process (all costs of implementation during the trial time period, and outcomes of implementation, aligned with RE-AIM); and the costs and outcomes of the intervention in the real-world to GP services (all costs to services associated with GooD4Mums for services during the trial time period, outcomes aligned with primary outcomes of the trial). The differences in costs and outcomes between the intervention and control sites will be identified. Costs will be separated in fixed and variable costs. An implementation cost calculator will be created to estimate the implementation and intervention costs for GooD4Mums based upon size of target population to inform other site's participation in the implementation scale up across Australia.

20b ***Methods for any additional analyses (e.g., subgroup and adjusted analyses)***

20c ***Definition of analysis population relating to protocol non-adherence (eg, as randomised analysis), and any statistical methods to handle missing data (eg, multiple imputation)***

Withdrawal of practices
We will report any reasons for practice withdrawal for each group and compare the reasons qualitatively.
In the event that some subjects have missing assessments at T3, a mixed model analysis will be applied using the method of residual (or restricted) maximum likelihood (REML). The only difference being that the reported means are no longer equivalent to simple arithmetic means but are predicted means that are recovered from the mixed model that has been fitted to the data.
Withdrawal of general practice clinicians, staff and patients from providing qualitative feedback
We will report any reasons for individual participant withdrawal for each group and compare the reasons qualitatively. If their feedback has not already been processed and anonymised as part of the evaluation, their data can be extracted and removed from inclusion in any analyses.
The effect that any missing data might have on results will be assessed via Baseline and evaluative data from individual participants who withdraw consent for participation.

Withdrawal of patients from de-identified data extraction via GRHANITE®
Any women with history of GDM who withdraw permission from having their de-identified data extracted via GRHANITE® will not be included on practice's Gestational Diabetes Registers.

Methods: Monitoring

Data monitoring 21a ***Composition of data monitoring committee (DMC); summary of its role and reporting structure; statement of whether it is independent from the sponsor and competing interests; and reference to where further details about its charter can be found, if not in the protocol. Alternatively, an explanation of why a DMC is not needed***

A Data Monitoring Committee is not required as a sub-set of the Project Steering Committee will fulfil the role.

21b ***Description of any interim analyses and stopping guidelines, including who will have access to these interim results and make the final decision to terminate the trial***

Data collection and its analysis will be occurring regularly throughout the trial. The hybrid
of the implementation trial does not necessitate interim-analysis on the primary outcomes mid-way through the trial as a means to assess whether trial termination is required.

Harms 22 ***Plans for collecting, assessing, reporting, and managing solicited and spontaneously reported adverse events and other unintended effects of trial interventions or trial conduct***

Risks for general practice participants
Based on the pilot study, we do not anticipate any risks to general practices, GPs, PNs, PMs or other staff participating in the learning workshop webinars, quality improvement activities, or evaluative surveys, interviews and/or focus groups. Clinicians and staff at general practices could feel uncomfortable about discussing their practice, particularly if providing negative details about the intervention, the take up of the intervention in the practice, or their relationship with other clinicians involved in the study. To manage these discomforts, we will undertake strategies outlined below (Risk Mitigation). We will ensure all data are de-identified for analysis, ensuring no identifying information is presented in study outputs.
There are five MDOs covering general practitioners. Current Medical Indemnity Insurance Policies for Doctors provide automatic coverage for work doctors undertake as investigators or co-investigators as part of any Medical Research or Clinical Trial. This cover is subject to the research trial being approval by a properly constituted human research ethics committee registered by the NHMRC; conducted in accordance with the requirements of that ethics committee; and within the Category for which the doctor is insured and for which the doctor is qualified. Despite 'automatic coverage', we will send the Good4Mum protocol to the MDOs for their explicit approval and thereby minimise legal risk to participating doctors.

Risk for women with gestational diabetes
There is potential that a woman without a diagnosis of gestational diabetes recorded in the EMR may be recalled for diabetes testing because they have past pathology test results in the EMR that indicate previous gestational diabetes (e.g. OGTT levels recorded during pregnancy). It may be that some of these women have not informed their GP about this, either not having thought to or not wishing their GP to know. GPs will triage the lists of women on the gestational diabetes recall register to check appropriateness of women being recalled. Recall letters to women will explain that their recall is either because of their earlier GDM diagnosis or because of other indications in their medical record that the recall is warranted (e.g. result of a past blood glucose tolerance test and other risk factors.

Risks for researchers .
We do not anticipate any safety concerns to our researchers / staff. All trial activities, aside from office-based work, will be conducted at participating GP practices, however, much of this will be facilitated via Zoom or other tele/videoconferencing medium. Nonetheless, we will have risk mitigation strategies in place (see below).

Risks related to GRHANITE® & GooD4Mum EMR / NDSS linked data

GRHANITE® design incorporates many privacy-protecting and data security measures (see Appendix 5, Introduction to GRHANITE) to protect both general practices, their staff and patients represented in the data. No identifying information are stored as part of the data extraction. The privacy protection measures in place for the general public also reduces risk to general practices. Combined, these measures significantly reduce risks to general practices in their contribution of practice EMR data to the Patron data repository.
Data extraction associated with the Data for Decisions / Patron program of work has been granted a waiver of patient consent (University of Melbourne Human Research Ethics Committee approval ID 1647396), in accordance with the NHMRC Statement on Ethical Conduct in Human Research.[56] The absence of person identifying information in the Patron data repository means the risk of harm to individuals whose data are captured is very low. Patients at individual general practices also have the option to withdraw their data from being extracted in the first place.
Data extraction associated with the Diabetes Australia NDSS NDR/NGDR has also been granted a waiver of patient consent, see below.
The tightly controlled GooD4Mum VM, management and access environments contribute to lowering any risk to the public and practices.

Risk mitigation for participants (GPs, PNs, PMs)
While we do not anticipate any major risks to participants in this study, general practice participants may feel uncomfortable or frustrated about discussing their practice, particularly if providing negative details about the intervention, the take up of the intervention in the practice, or their relationship with other clinicians involved in the study. To reduce the potential for distress the researchers will provide, during recruitment and before any learning workshops or data collection activities, clear explanations about why the activities are being conducted, how the information will be used, and the kinds of evaluative questions that will be asked. Researchers will emphasize that:

- All information shared is confidential (unless we are legally compelled to disclose) and information about individuals will not be made available to any other agency;
- Responding to questions is entirely voluntary; if a participant prefers not to answer a specific question they do not have to;
- Interviews can be stopped at any point and learning sessions can be left at any point, if required.

Despite attempts to reduce the risk distress, there may be times a participant feels upset during an interview or activity. The researcher will acknowledge the distress, give the participant opportunity to talk about how they are feeling, and listen supportively and empathically. A break or postponement may be taken, or the participant is free to cease the interview entirely.

Risk mitigation for participants (patients)
While we do not anticipate any major risks to patients providing feedback, participants may feel uncomfortable or frustrated about discussing their experience, particularly if providing negative details about the practice, their recall or consultations, or their relationship with the clinicians. To reduce the potential for distress the researchers will provide, prior to interview commencement, a plain language statement with clear explanations about the purpose of the study and the interview, how the information will be used, and the kinds of evaluative questions that will be asked. Researchers will emphasize that:

- All information shared is confidential (unless we are legally compelled to disclose) and information about individuals will not be made available to the practice or other agency;
- Responding to questions is entirely voluntary; if a participant prefers not to answer a specific question they do not have to;

- Interviews can be stopped at any point and learning sessions can be left at any point, if required.

Despite attempts to reduce the risk distress, there may be times a participant feels upset during an interview or activity. The researcher will acknowledge the distress, give the participant opportunity to talk about how they are feeling, and listen supportively and empathically. A break or postponement may be taken, or the participant is free to cease the interview entirely.

Risk mitigation for researchers

Any clinic liaison staff will be trained on confidentiality and safety, and on how to appropriately respond to GPs, PNs and staff. Training will include the skills required to facilitate putting participants at ease, ensuring that they are not put under any pressure to answer any questions that they do not wish to, and that they do not feel judged or blamed as a result of any response that they give.
The GooD4Mum protocol includes data handling procedures for researchers, which provide process for mitigating risks associated with data collection, management and use.

Risk mitigation – use of EMR data

Australian Medical Defence Organisations (MDOs), indemnity insurers for general practices, have approved the Data for Decisions program that frames EMR data collection and storage in the Patron repository; their approval reduces risk to general practices insured by the MDOs.
Only Ethics Committee and Patron Data Governance Committee approved GooD4Mum researchers will be able to access EMR data. Before doing so the approved researchers must complete online Patron compliance training and sign a *Data Access Acknowledgement for Individuals.* The EMR data cannot be shared with anyone other than people named in the ethics and data access applications and whom have undertaken the required training.
Only approved researchers will be given log in access to the locked down GooD4Mum VM that houses the GooD4Mum tranche of Patron data. Two-factor authentication is required to login to the GooD4Mum EMR data repository. The GooD4Mum VM includes software applications for data analysis (e.g. Stata, SQL Server Management Studio, SPSS). No raw data can be exported outside of the VM. Any data available outside of the secure VM will be aggregated for results reporting.
Recipients of Patron EMR data are obliged to notify the Patron Data Steward immediately upon becoming aware of any actual or suspected data breach occurring with relation to any EMR derived data held, such as noticing person identifying data in any field or loss of data through a security breach. This obligation is outlined and reiterated in the *Data Access Agreement, Data Access Acknowledgement for Individuals* and in the Patron Compliance training.

____

Future proofing the study: SARS-CoV-2

SARS-CoV-2 (COVID-19) was declared a pandemic on 11 March 2020. The pandemic has significantly impacted on healthcare needs and provision of healthcare services. Primary care workflows have changed due to engagements in telehealth, COVID-19 testing and COVID-19 vaccinations. On 10th January 2022, children aged 5-11 years were able to receive COVID-19 vaccinations and many are doing so via a local general practice. In June 2022 influenza vaccination became free to the community, again placing high demand on general practices. Illness and burnout among general practice staff has been high, with high rates of staff absence and turnover. These additional workloads have placed stress of the general practice community.
During 2020-2022, VicREN recruiters have met with great resistance from staff at general practices who reported being unwilling to consider additional responsibilities such as participation in research activities, and recruitment for a

majority of studies was paused or modified; this has caused additional pressure on general practice recruitment as paused projects are now joining newly funded projects to recruit practices.
Recruitment for GooD4Mum is to take place within this environment and we have the following strategies in place to minimise risks and to future-proof the trial.

Extension to grant period

A 12-month extension to the NHMRC Partnership grant has been sought and approved, this provides some leeway for recruitment and trial commencement timeline.

Modification of research design

Adopting pragmatic modifications of research design, as required, to enable rigorous completion of the study.

Maximise existing networks and recruitment opportunities

Recruitment via VicREN maximises recruitment potential, with recruiters able to review notes on past contact with practices to gain understanding of their recent reactions to recruitment requests. By focusing initial recruitment on practices that are already within the Patron/Data for Decisions network (140+ practices) and whom therefore already have GRHANITE® installed, this decreases the need to engage general practices that may be unwilling to consider consenting to activities that include the additional step of software installation.

Contactless visits, consent, meetings and data collection

GooD4Mum recruitment will largely be without physical practice visits, making use of VicREN processes for remote recruitment and eConsent.
Meetings between researchers and stakeholders will, as appropriate, take place remotely. Where possible, data collection will also be undertaken remotely. When physical visits are required, staff will follow University approved SOPs for travelling to off-site locations (see next).

Training and processes for practice liaison staff – Face-to-face visits

GooD4Mum practice liaison staff will have all required vaccinations and have completed all necessary training on processes and protocols prior to undertaking practice visits. Staff will be familiar with the SOP for travelling to off-site locations that are informed by government directives and data on the spread of COVID-19. The SOPs seek to mitigate risks by ensuring that measures will be taken to decrease risk of exposure to and transmission of the virus. This includes undertaking risk assessment, pre-requisite training, use of personal protective equipment (PPE), emergency procedures, incident management, and travel checklists.

eResources for practices

All resources for practices will be available as soft-copy and web-based resources to limit need for paper printouts.

Auditing | 23 | ***Frequency and procedures for auditing trial conduct, if any, and whether the process will be independent from investigators and the sponsor***

As an implementation trial of QIC methods to prompt practice change, there is no prescribed protocol for participating practices, staff or patients to adhere to, other than practice representative attendance at quarterly learning workshops (webinars) and completing four PDSA cycles to prompt clinic team improvements in management and follow up of women with past history of gestational diabetes.

Good4Mum practice liaison staff accessing the GooD4Mum project Dynamics (client management module) have the ability to audit contact and milestone achievement with practices. These staff are responsible for ensuring correct completion of compliance documents by practices, their return to the research team, input into the Dynamics practice liaison management system, filing of electronic and any hard copies, and following up practices that have not submitted all relevant documents.

Good4Mum practice liaison staff will also keep a record of practice participation in trial activities and make arrangements for logged continuing professional development (CPD) points / hours to be converted in the certificates for participants.
If an audit makes apparent a problem of process, communication or documentation, related to the larger trial or to a single participating site, the practice liaison staff, in consultation with the practice and with input from chief investigators if required, will resolve the issue.

Ethics and dissemination

Research ethics approval	24	***Plans for seeking research ethics committee/institutional review board (REC/IRB) approval***<br><br>This protocol and the template informed consent forms contained in Appendix 2 will be reviewed and approved by the sponsor and the University of Melbourne Human Research Ethics Committee (HREC) with respect to scientific content and compliance with applicable research and human subject regulations.<br>The protocol, informed consent forms, plain language information statements and recruitment materials, and other requested documents – and any subsequent modifications –will also be reviewed and approved by HREC. Annual reports will be submitted to HREC.
Protocol amendments	25	***Plans for communicating important protocol modifications (eg, changes to eligibility criteria, outcomes, analyses) to relevant parties (eg, investigators, REC/IRBs, trial participants, trial registries, journals, regulators)***<br><br>This study will be conducted in compliance with the protocol. Any change to the protocol document or Informed Consent Form that affects the scientific intent, study design, participant safety, or may affect a participants willingness to continue participation in the study is considered an amendment, and therefore will be written and filed as an amendment to this protocol and/or informed consent form. All amendments will be agreed by the GooD4Mum Steering Committee) and submitted to the HREC for approval prior to becoming effective.<br>Administrative changes of the protocol, i.e. minor corrections and/or clarifications that have no effect on the way the study is to be conducted, will be agreed by the Work Package Lead and Project Lead and documented. HREC will be notified of administrative changes when required.
Consent or assent	26a	***Who will obtain informed consent or assent from potential trial participants or authorised surrogates, and how (see Item 32)***<br><br>Trained GooD4Mum recruitment officers or Practice Liaison staff associated with VicREN will introduce the trial to the practices, normally this is via the Practice Manager, but it may be through another contact such as a GP member of the VicREN research network. If representatives from an eligible practice are interested after receiving a recruitment notice / flyer, they will be provided with an information pack that includes a GooD4Mum plain language information statement and consent form, and plain language information about Data for Decisions, and will also be given opportunity to have a face-to-face or Zoom (or equivalent) meeting where they may be shown a short PowerPoint presentation and will have opportunity to have an informed discussion and ask questions.<br>The GooD4Mum liaison staff will obtain a signed MOU/Legal Agreement (and procure countersignature by the University of Melbourne project lead), and signed written consent from the nominated GooD4Mum practice champion.

Additionally, informed consent of individual GPs, practice nurses, practice managers or other staff, other stakeholders and patients will be sought by researchers undertaking the implementation evaluation (WP6 and 7) prior to conducting surveys or performing feedback interviews which may be at any time from baseline to after completion of the trial.

26b ***Additional consent provisions for collection and use of participant data and biological specimens in ancillary studies, if applicable***

Consent documents will include information about potential reuse of aggregate and outcomes data generated as part of the GooD4Mum trial and its evaluation. It may be re-used for a longitudinal GooD4Mum continuation study and we will request participation in HeSANDA (Health Studies National Data Asset program, Australian Research Data Commons, a nationally distributed network of health data sharing infrastructure with a node at the University of Melbourne through the Methods and Implementation Support for Clinical and Health research hub (MISCH)). "HeSANDA seeks to increase research impact and integrity by supporting further research, meta-analysis, and clinical guideline development. By creating a national asset from the data outputs of health research projects, HeSANDA hopes to maximise the investment into health research by schemes such as the NHMRC Clinical Trials and Cohort Studies (CTCS) program."
https://ardc.edu.au/collaborations/strategic-activities/national-data-assets/health-studies-national-data-asset-program/

A waiver of consent is in place for data extraction associated with the Data for Decisions / Patron program of work (University of Melbourne Human Research Ethics Committee approval ID 1647396 in accordance with the NHMRC Statement on Ethical Conduct in Human Research[56]).
A waiver of consent is also in place for data extraction and linkage associated with WP2 use of National Diabetes Register / National Gestational Diabetes Register (University of Melbourne Human Research Ethics Committee approval ID# 2021-22457-21786-3, in accordance with the NHMRC Statement on Ethical Conduct in Human Research[56]).

Confidentiality 27 ***How personal information about potential and enrolled participants will be collected, shared, and maintained in order to protect confidentiality before, during, and after the trial***

Participant confidentiality will be strictly held in trust by the participating investigators, research staff, and the sponsoring institution and their agents. The study protocol, documentation, data and all other information generated will be held in strict confidence. No information concerning the study, or the data will be released to any unauthorised third party, without prior approval by the participant and written approval of the sponsoring institution. Refer also to the Confidentiality clause in the MOU/Legal Agreement between participating practices and the GooD4Mum team (Appendix 1).

Survey, interview and focus group data will be de-identified for analysis. Any personal information revealed in interviews will be anonymised. Any quotes attributed to individual participants in published form will be anonymised. **eConsent forms** will be stored separate to interview data but in the same restricted access folder.

De-identified EMR data in the Patron repository
The Data for Decisions/Patron data governance and ethical framework allows for de-identified patient data to be extracted via GRHANITE® from participating

GooD4Mum practices without need for explicit patient consent due to the approved waiver of patient consent (compliant with NHMRC guidelines). The Data for Decisions **Information for Patients** flyer includes information about withdrawing from the Program and a form to action withdrawal. Participating general practices are given instructions explaining the steps to take to withdraw an individual patient from having their data collected in the GRHANITE® data extraction. Data that has previously been extracted related to an individual patient can be removed from the Patron primary care repository, but it cannot be removed from extracted Patron datasets already in use by researchers.
Explicit approval from the independent Patron Data Governance Committee is required prior to access data in the repository unless the general practice has provided explicit, informed consent to share their data for the GooD4Mum trial. General practices that are not existing Data for Decisions (ePBRN) practices but are onboarding to Data for Decisions as part of the GooD4Mum trial, will have option to share their data only for the GooD4Mum trial or to allow their data to be used for other research projects approved by the Patron Data Governance Committee.
Good4Mum researchers can access practices EMR data only after:

1. obtaining explicit approval by the Patron Data Governance Committee,
2. executing a Data Access Agreement (for Patron data access by persons not part of The University of Melbourne) and a Memorandum of Understanding (MOU for researchers internal to the University of Melbourne). The Data Access Agreement/MOU specifies contract details, licence term and specifications around the tranche of data allowed to be accessed by the GooD4Mum team.
3. acknowledging the Contractual Project Plan – a high-level plan that is referenced by the legal agreement/MOU that provides specific details to complement the legal agreement/MOU (if agreed by both parties, the project plan can be altered without requirement to amend the executed legal agreement).
4. individual researchers who will access the data sign a Data Access Acknowledgement and complete online Compliance Training.

Declaration of interests | 28 | ***Financial and other competing interests for principal investigators for the overall trial and each study site***

Investigator CIA (Prof Helena Teede) declares no real or perceived financial or other competing interests for the overall study nor relating to any study site.

Investigator CIB – Retired, no longer involved (James Dunbar).

Investigator CIC - Prof Douglas Boyle is the Data Steward for the Patron primary care data repository, part of the University of Melbourne's Data for Decisions research initiative which provides the data governance and security framework for the gathering and curation of the de-identified practice EMR data that will be used during the GooD4Mum trial. He is also the developer of the GRHANITE® data extraction tool that will be installed in each practice, but he gains no financial interest for use of this software.

Investigator CID A/Prof Vincent Versace

Investigator CIE Prof Jane Speight

Investigator CIF Dr Siew Lim

Investigator CIG Prof Mark Morgan

Investigator CIH A/Prof Sharleen O'Reilly declares no real or perceived financial or other competing interests for the overall study nor relating to any study site.

Investigator CII Dr Dale Ford – deceased (23/5/2024)

Investigator CIJ A/Prof Emily Callander

Research Fellow: Dr Rachel Canaway was a member of the team that established Data for Decisions and the Patron program of research. Prof Boyle and Dr Canaway receive no financial gain from GRHANITE® and Patron data being used in the GooD4Mum study.

Access to data	29	***Statement of who will have access to the final trial dataset, and disclosure of contractual agreements that limit such access for investigators***

The GooD4Mum Project Steering Committee will have responsibility for intra-study data sharing processes and management.
As previously outlined, only ethics and Patron data governance committee approved researchers will have access to EMR data unless aggregate data or report tables are exported from the secure VM (a process which is facilitated by the HaBIC R2 technical team whom are independent of GooD4Mum).
Good4Mum WP5 data and documents will be housed in the GooD4Mum folder on the University of Melbourne managed SharePoint drive and will be accessible only to those with set permissions.

Work Package Principal Investigators will have direct access to their own data sets and will have access to other sets of data on request. To ensure confidentiality, data dispersed to project team members will be deidentified at the level of the individual.

Ancillary and post-trial care	30	***Provisions, if any, for ancillary and post-trial care, and for compensation to those who suffer harm from trial participation***

As outlined above (Harms #22), risk of harm through participation in the GooD4Mum trial is minimal and support of MDOs will be explicitly sought to cover participating doctors.
Support to practices will be provided by the GooD4Mum team prior to and subsequent to the trial.

Dissemination policy	31a	***Plans for investigators and sponsor to communicate trial results to participants, healthcare professionals, the public, and other relevant groups (eg, via publication, reporting in results databases, or other data sharing arrangements), including any publication restrictions***

Data analysis and release of results
The study data will be analysed and reported. All presentations and publications are expected to protect the integrity of the primary objectives of the study. Recommendations as to the timing of presentation of endpoint data and the gatherings at which they might be presented will be given by the GooD4Mum Project Steering Committee and will be guided by the recommendations of the **Work Package 8** Team (responsible for dissemination and scale up planning and implementation of dissemination and scale up strategies).

As part of Work Package 8, a GooD4Mum maintenance and scale-up plan will be co-designed with partners and key stakeholders to assist and enable implementation and scale of this project. The *GooD4Mum Dissemination and Communications Plan* will be developed iteratively informed by Work Packages 1 to 7. The *Dissemination and Communications Plan* will include a branding strategy and plans for:

- newsletters and website content;
- implementing our co-designed consumer messages on risk, screening and lifestyle via routine National Gestational Diabetes Register alerts to women on the register, embedding these into the Victorian Government Department of Health and Human Services funded Life! Program and making them available to practices as a resource for women;
- prompting scale-up of our T2DM screening and prevention plan implementation toolkit and resources within national Primary Health Networks (PHNs) and
- how best to influence update of the RACGP and Diabetes Australia's national T2DM guidelines,
- reporting of study results to participating practices, partners and other stakeholders;
- print, online and social media content for disseminating project findings to the broader community;
- research translation workshops with partners, health professional organisations and the Federal Department of Health;

GooD4Mum Protocol Paper
The protocol paper will be drafted and authorship granted on the basis of contributors meeting the authorship criteria outlined at 31b. The journal for submission of this paper will be agreed by the Chief Investigators.

Primary outcome papers
Manuscript(s) for presenting primary outcome data. Determination of whether particular analysis represents a primary outcome will be made by the Project Steering Committee based on a clear planning template. Authorship will be granted on the basis of contributors meeting the authorship criteria outlined at 31b.

Other study papers, abstracts and presentations
Each Work Package Team will take responsibility for coordination of presentations or publications where reporting is limited to specific aspects of their Work Package, for example, a publication/presentation reporting on findings of data exploration from Work Package 2, or on messaging development undertaken by the Work Package 3 Team. Authorship is granted on the basis of contributors meeting the authorship criteria outlined at 31b.

31b *Authorship eligibility guidelines and any intended use of professional writers*

Topics suggested for presentation or publication will be circulated to the Chief Investigators. If the topic originates from activities coordinated by a defined Work Package Team, the team is requested to suggest and justify names for authors. The agreed group of co-authors will determine their contributions and the order of authorship.

Authorship will be granted on the basis of meeting the criteria of the International Committee of Medical Journal Editors (ICMJE). All designated authors

should meet all four criteria for authorship, and all who meet the four criteria should be identified as authors:[57]

1. Substantial contributions to the conception or design of the work; or the acquisition, analysis, or interpretation of data for the work; AND
2. Drafting the work or revising it critically for important intellectual content; AND
3. Final approval of the version to be published; AND
4. Agreement to be accountable for all aspects of the work in ensuring that questions related to the accuracy or integrity of any part of the work are appropriately investigated and resolved.

"An author should be able to identify which co-authors are responsible for specific other parts of the work. In addition, authors should have confidence in the integrity of the contributions of their co-authors. Those who do not meet all four criteria should be acknowledged".[57]

Examples of activities that alone (without other contributions) do not qualify authorship are: acquisition of funding; general supervision of a research group or general administrative support; and writing assistance, technical editing, language editing, and proofreading.

Acknowledgement when contributions do not justify authorship:
Acknowledgement may be of an individual or a group under a single heading[57] (e.g. GooD4Mum investigators or GooD4Mum research group). Acknowledged persons contributions should be specified (e.g. served on the Expert Reference Panel, critically reviewed the study proposal, collected data, provided training to study participants, participated in writing or technical editing of the manuscript). Acknowledgment may imply endorsement by acknowledged individuals of a study's data and conclusions, therefore authors should ensure that written permission to acknowledge is received from all acknowledged individuals.[57]

When a member of a team leaves the research group – and that member has contributed substantially to the conception or design of the work or to the acquisition, analysis, or interpretation of data – then that member should be contacted, if possible, and asked if they wish to be a co-author via active contribution to criteria 2, 3 and 4 above. No remuneration will be offered in exchange for their effort.

Disputes regarding authorship will be settled by the lead of the relevant Work Package team or escalated to the Study Principal if required.

31c ***Plans, if any, for granting public access to the full protocol, participant-level dataset, and statistical code***

Appendices

Informed consent materials 32 ***Model consent form and other related documentation given to participants and authorised surrogates***

Good4Mum plain language information statement for:

- intervention general practice staff,
- Intervention general practice GooD4Mum Practice Champion
- Consumer participants (intervention practices)
- Stakeholders-partners

- Control practices (Data for decision practice, for late invite to participate in interviews/focus groups).

Consent forms for:

- Intervention general practice staff participants,
- consumer participants (intervention practices)
- stakeholder-partners,
- control practices,
- control practice staff.
- Verbal consent messaging

Recruitment messaging

1 page recruitment flyer, intervention practices

1 page recruitment flyer, control practices

Biological specimens	33	***Plans for collection, laboratory evaluation, and storage of biological specimens for genetic or molecular analysis in the current trial and for future use in ancillary studies, if applicable*** Not applicable.
Recruitment flowcharts		*Flow of practice recruitment, contact and data collection* *GooD4Mum, Decision pathways for practice participation*
Other documents		Project governance framework Methods, data collection, analysis and evaluation plan (WP5,6,7) Schedule of data collection activities (interaction with intervention and control practices from recruitment to trial end) Study Timeline Legal Agreement between consenting practices and the University of Melbourne Data risk and management plan Introduction to GRHANITE® flyer Information about Data for Decisions for ethics committees Information about Future Health Today for ethics committees

*It is strongly recommended that this checklist be read in conjunction with the SPIRIT 2013 Explanation & Elaboration for important clarification on the items. Amendments to the protocol should be tracked and dated. The SPIRIT checklist is copyrighted by the SPIRIT Group under the Creative Commons "Attribution-NonCommercial-NoDerivs 3.0 Unported" license.

References

1. Li Z, Cheng Y, Wang D, Chen H, Chen H, Ming WK, et al. Incidence Rate of Type 2 Diabetes Mellitus after Gestational Diabetes Mellitus: A Systematic Review and Meta-Analysis of 170,139 Women. Journal of Diabetes Research. 2020;2020:3076463. doi:10.1155/2020/3076463
2. Evans M, Morgan AR, Patel D, Dhatariya K, Greenwood S, Newland-Jones P, et al. Risk Prediction of the Diabetes Missing Million: Identifying Individuals at High Risk of Diabetes and Related Complications. Diabetes Ther. 2021;12(1):87-105. doi:10.1007/s13300-020-00963-2
3. Nielsen KK, Kapur A, Damm P, de Courten M, Bygbjerg IC. From screening to postpartum follow-up - the determinants and barriers for gestational diabetes mellitus (GDM) services, a systematic review. BMC Pregnancy Childbirth. 2014;14:41. doi:10.1186/1471-2393-14-41

4. Vounzoulaki E, Khunti K, Abner SC, Tan BK, Davies MJ, Gillies CL. Progression to type 2 diabetes in women with a known history of gestational diabetes: systematic review and meta-analysis. Bmj. 2020;369:m1361. doi:10.1136/bmj.m1361
5. Dennison RA, Chen ES, Green ME, Legard C, Kotecha D, Farmer G, et al. The absolute and relative risk of type 2 diabetes after gestational diabetes: A systematic review and meta-analysis of 129 studies. Diabetes Research and Clinical Practice. 2021;171:108625. doi:10.1016/j.diabres.2020.108625
6. Knowler WC, Barrett-Connor E, Fowler SE, Hamman RF, Lachin JM, Walker EA, et al. Reduction in the incidence of type 2 diabetes with lifestyle intervention or metformin. N Engl J Med. 2002;346(6):393-403. doi:10.1056/NEJMoa012512
7. Hedeager Momsen AM, Høtoft D, Ørtenblad L, Friis Lauszus F, Krogh RHA, Lynggaard V, et al. Diabetes prevention interventions for women after gestational diabetes mellitus: an overview of reviews. Endocrinology, Diabetes and Metabolism. 2021. doi:10.1002/edm2.230
8. Lindström J, Ilanne-Parikka P, Peltonen M, Aunola S, Eriksson JG, Hemiö K, et al. Sustained reduction in the incidence of type 2 diabetes by lifestyle intervention: follow-up of the Finnish Diabetes Prevention Study. Lancet. 2006;368(9548):1673-9. doi:10.1016/s0140-6736(06)69701-8
9. Voerman E, Santos S, Patro Golab B, Amiano P, Ballester F, Barros H, et al. Maternal body mass index, gestational weight gain, and the risk of overweight and obesity across childhood: An individual participant data meta-analysis. PLoS Med. 2019;16(2):e1002744. doi:10.1371/journal.pmed.1002744
10. National Institute for Health and Care Excellence. Diabetes in pregnancy: management from preconception to the postnatal period. NICE Guideline. [Internet]. 2015 [updated 2020] 12 Nov 2021 [cited 2021 12 November]:[40 p.]. Available from: https://www.nice.org.uk/guidance/ng3.
11. O'Reilly SL, Versace VL, Skinner TC, Dunbar JA, the MSG. Women's engagement with diabetes prevention activities and the influence of contact by the Australian national gestational diabetes register. Practical Diabetes. 2021;38(3):14-9b. doi:https://doi.org/10.1002/pdi.2336
12. IHI. The Breakthrough Series: IHI's Model for Achieving Breakthrough Improvement 2003 [cited 2021 17 November]:[16 p.]. Available from: http://www.ihi.org/resources/Pages/IHIWhitePapers/TheBreakthroughSeriesIHIsCollaborativeModelforAchievingBreakthroughImprovement.aspx.
13. de la Perrelle L, Cations M, Barbery G, Radisic G, Kaambwa B, Crotty M, et al. How, why and under what circumstances does a quality improvement collaborative build knowledge and skills in clinicians working with people with dementia? A realist informed process evaluation. BMJ Open Quality. 2021;10(2):e001147. doi:10.1136/bmjoq-2020-001147
14. Nadeem E, Olin SS, Hill LC, Hoagwood KE, Horwitz SM. Understanding the Components of Quality Improvement Collaboratives: A Systematic Literature Review. The Milbank Quarterly. 2013;91(2):354-94. doi:https://doi.org/10.1111/milq.12016
15. O'Reilly SL, Dunbar JA, Best JD, Versace V, Ford D, Young D, et al. GooD4Mum: A general practice-based quality improvement collaborative for diabetes prevention in women with previous gestational diabetes. Primary Care Diabetes. 2019;13(2):134-41. doi:https://doi.org/10.1016/j.pcd.2018.10.006
16. Luckenbaugh AN, Miller DC, Ghani KR. Collaborative quality improvement. Curr Opin Urol. 2017;27(4):395-401. doi:10.1097/mou.0000000000000404
17. Wells S, Tamir O, Gray J, Naidoo D, Bekhit M, Goldmann D. Are quality improvement collaboratives effective? A systematic review. BMJ Qual Saf. 2018;27(3):226-40. doi:10.1136/bmjqs-2017-006926
18. Knight AW, Ford D, Audehm R, Colagiuri S, Best J. The Australian Primary Care Collaboratives Program: improving diabetes care. BMJ Quality & Safety. 2012;21(11):956. doi:10.1136/bmjqs-2011-000460
19. Absetz P, Oldenburg B, Hankonen N, Valve R, Heinonen H, Nissinen A, et al. Type 2 diabetes prevention in the real world: three-year results of the GOAL lifestyle implementation trial. Diabetes care. 2009;32(8):1418-20. doi:10.2337/dc09-0039
20. Absetz P, Valve R, Oldenburg B, Heinonen H, Nissinen A, Fogelholm M, et al. Type 2 diabetes prevention in the "real world": one-year results of the GOAL Implementation Trial. Diabetes Care. 2007;30(10):2465-70. doi:10.2337/dc07-0171
21. Laatikainen T, Dunbar JA, Chapman A, Kilkkinen A, Vartiainen E, Heistaro S, et al. Prevention of Type 2 Diabetes by lifestyle intervention in an Australian primary health care setting: Greater Green Triangle (GGT) Diabetes Prevention Project. BMC Public Health. 2007;7(1):249. doi:10.1186/1471-2458-7-249
22. Oldenburg B, Absetz P, Dunbar JA, Reddy P, O'Neil A. The spread and uptake of diabetes prevention programs around the world: a case study from Finland and Australia. Transl Behav Med. 2011;1(2):270-82. doi:10.1007/s13142-011-0046-y
23. Dunbar JA, Hernan AL, Janus ED, Vartiainen E, Laatikainen T, Versace VL, et al. Challenges of diabetes prevention in the real world: results and lessons from the Melbourne Diabetes Prevention Study. BMJ Open Diabetes Res Care. 2015;3(1):e000131-e. doi:10.1136/bmjdrc-2015-000131

24. O'Reilly SL, Dunbar JA, Versace V, Janus E, Best JD, Carter R, et al. Mothers after Gestational Diabetes in Australia (MAGDA): a randomised controlled trial of a postnatal diabetes prevention program. PLoS medicine. 2016;13(7):e1002092.
25. Lim S, Versace VL, O'Reilly S, Janus E, Dunbar J. Weight change and Cardiometabolic outcomes in postpartum women with history of gestational diabetes. Nutrients. 2019;11(4):922.
26. Shih ST, Davis-Lameloise N, Janus ED, Wildey C, Versace VL, Hagger V, et al. Mothers After Gestational Diabetes in Australia Diabetes Prevention Program (MAGDA-DPP) post-natal intervention: an update to the study protocol for a randomized controlled trial. Trials. 2014;15(1):1-2.
27. Wilkinson SA, Lim SS, Upham S, Pennington A, O'Reilly SL, Asproloupos D, et al. Who's responsible for the care of women during and after a pregnancy affected by gestational diabetes? Med J Aust. 2014;201(3 Suppl):S78-81. doi:10.5694/mja14.00251
28. Pennington AV, O'Reilly SL, Young D, Dunbar JA. Improving follow-up care for women with a history of gestational diabetes: perspectives of GPs and patients. Australian Journal of Primary Health. 2017;23(1):66-74.
29. Keely E. An opportunity not to be missed--how do we improve postpartum screening rates for women with gestational diabetes? Diabetes Metab Res Rev. 2012;28(4):312-6. doi:10.1002/dmrr.2274
30. Keely E, Clark H, Karovitch A, Graham I. Screening for type 2 diabetes following gestational diabetes: family physician and patient perspectives. Canadian Family Physician. 2010;56(6):558-63.
31. Ratner RE, Christophi CA, Metzger BE, Dabelea D, Bennett PH, Pi-Sunyer X, et al. Prevention of Diabetes in Women with a History of Gestational Diabetes: Effects of Metformin and Lifestyle Interventions. The Journal of Clinical Endocrinology & Metabolism. 2008;93(12):4774-9. doi:10.1210/jc.2008-0772
32. Lim S, Hill B, Teede HJ, Moran LJ, O'Reilly S. An evaluation of the impact of lifestyle interventions on body weight in postpartum women: A systematic review and meta-analysis. Obesity Reviews. 2020;21(4):e12990. doi:https://doi.org/10.1111/obr.12990
33. Boyle DI, Versace VL, Dunbar JA, Scheil W, Janus E, Oats JJ, et al. Results of the first recorded evaluation of a national gestational diabetes mellitus register: Challenges in screening, registration, and follow-up for diabetes risk. PloS one. 2018;13(8):e0200832. doi:10.1371/journal.pone.0200832
34. Wolfenden L, Foy R, Presseau J, Grimshaw JM, Ivers NM, Powell BJ, et al. Designing and undertaking randomised implementation trials: guide for researchers. BMJ. 2021;372:m3721. doi:10.1136/bmj.m3721
35. Future Health Today - Clinical decision support for primary care Melbourne: The University of Melbourne; 2023 [cited 2023 13 September]. Available from: https://futurehealthtoday.com.au/.
36. Ilott I, Gerrish K, Booth A, Field B. Testing the Consolidated Framework for Implementation Research on health care innovations from South Yorkshire. Journal of evaluation in clinical practice. 2013;19(5):915-24. doi:10.1111/j.1365-2753.2012.01876.x
37. Damschroder LJ, Lowery JC. Evaluation of a large-scale weight management program using the consolidated framework for implementation research (CFIR). Implementation Science. 2013;8(1):51. doi:10.1186/1748-5908-8-51
38. Safaeinili N, Brown-Johnson C, Shaw JG, Mahoney M, Winget M. CFIR simplified: Pragmatic application of and adaptations to the Consolidated Framework for Implementation Research (CFIR) for evaluation of a patient-centered care transformation within a learning health system. Learning Health Systems. 2020;4(1):e10201. doi:https://doi.org/10.1002/lrh2.10201
39. May C, Rapley T, Mair F, Treweek S, Murray E, Ballini L, et al. Normalization Process Theory On-line Users' Manual, Toolkit and NoMAD instrument 2015 [cited 2021 19 December]. Available from: http://www.normalizationprocess.org.
40. Murray E, Treweek S, Pope C, MacFarlane A, Ballini L, Dowrick C, et al. Normalisation process theory: a framework for developing, evaluating and implementing complex interventions. BMC Medicine. 2010;8(1):63. doi:10.1186/1741-7015-8-63
41. Glasgow RE, Vogt TM, Boles SM. Evaluating the public health impact of health promotion interventions: the RE-AIM framework. Am J Public Health. 1999;89(9):1322-7. doi:10.2105/ajph.89.9.1322
42. Glasgow RE, Harden SM, Gaglio B, Rabin B, Smith ML, Porter GC, et al. RE-AIM Planning and Evaluation Framework: Adapting to New Science and Practice With a 20-Year Review. Frontiers in Public Health. 2019;7(64). doi:10.3389/fpubh.2019.00064
43. Gaglio B, Shoup JA, Glasgow RE. The RE-AIM framework: a systematic review of use over time. Am J Public Health. 2013;103(6):e38-46. doi:10.2105/ajph.2013.301299
44. Brunisholz KD, Kim J, Savitz LA, Hashibe M, Gren LH, Hamilton S, et al. A Formative Evaluation of a Diabetes Prevention Program Using the RE-AIM Framework in a Learning Health Care System, Utah, 2013-2015. Preventing chronic disease. 2017;14:E58. doi:10.5888/pcd14.160556
45. University of Melbourne Department of General Practice and Primary Care. Data for Decisions and the Patron program of resarch Melbourne: The University of Melbourne; ND [cited 2023 4 August]. Available from: www.gp.unimelb.edu.au/datafordecisions.

46. Finch TL, Girling M, May CR, Mair FS, Murray E, Treweek S, et al. Nomad: Implementation measure based on Normalization Process Theory [Measurement Instrument] 2015. Available from: http://www.normalizationprocess.org.
47. RACGP. Standards for general practices, 5th edition. East Melbourne: The Royal Australian College of General Practitioners; 2023 [cited 24 July 2023]. Available from: https://www.racgp.org.au/running-a-practice/practice-standards/standards-5th-edition/.
48. Bogdanet D, Reddin C, Macken E, Griffin TP, Fhelelboom N, Biesty L, et al. Follow-up at 1 year and beyond of women with gestational diabetes treated with insulin and/or oral glucose-lowering agents: a core outcome set using a Delphi survey. Diabetologia. 2019;62(11):2007-16. doi:10.1007/s00125-019-4935-9
49. Soos M, Temple-Smith M, Gunn J, Johnston-Ata'Ata K, Pirotta M. Establishing the Victorian primary care practice based research network. Australian Family Physician. 2010;39(11):857.
50. Shah S, Roydhouse JK, Toelle BG, Mellis CM, Jenkins CR, Edwards P, et al. Recruiting and retaining general practitioners to a primary care asthma-intervention study in Australia. Aust J Prim Health. 2014;20(1):98-102. doi:10.1071/py12093
51. Finch TL, Girling M, May CR, Mair FS, Murray E, Treweek S, et al. Improving the normalization of complex interventions: part 2 - validation of the NoMAD instrument for assessing implementation work based on normalization process theory (NPT). BMC Medical Research Methodology. 2018;18(1):135. doi:10.1186/s12874-018-0591-x
52. Zwar NA, Richmond RL, Halcomb EJ, Furler JS, Smith JP, Hermiz O, et al. Quit in general practice: a cluster randomized trial of enhanced in-practice support for smoking cessation. Family Practice. 2015;32(2):173-80. doi:10.1093/fampra/cmu089
53. Guest G, Bunce A, Johnson L. How Many Interviews Are Enough?: An Experiment with Data Saturation and Variability. Field Methods. 2006;18(1):59-82. doi:10.1177/1525822X05279903
54. Saunders B, Sim J, Kingstone T, Baker S, Waterfield J, Bartlam B, et al. Saturation in qualitative research: exploring its conceptualization and operationalization. Qual Quant. 2018;52(4):1893-907. doi:10.1007/s11135-017-0574-8
55. QSR International. NVivo Qualitative Data Analysis Software [NVivo 12 Plus]. Melbourne2020.
56. Australian Government, National Health and Medical Research Council (NHMRC). National statement on ethical conduct in human research. Canberra: Australian Government, National Health and Medical Research Council; 2007 (Updated 2018) [cited 2021 9 December]. Available from: https://www.nhmrc.gov.au/guidelines-publications/e72.
57. ICMJE. Defining the role of authors and contributors: International Committee of Medical Journal Editors; 2021 [cited 2021 15 December]. Available from: http://www.icmje.org/recommendations/browse/roles-and-responsibilities/defining-the-role-of-authors-and-contributors.html.
